# Factors Associated With Child Stunting, Wasting, and Underweight in 35 Low- and Middle-Income Countries

**DOI:** 10.1001/jamanetworkopen.2020.3386

**Published:** 2020-04-22

**Authors:** Zhihui Li, Rockli Kim, Sebastian Vollmer, S. V. Subramanian

**Affiliations:** 1 Department of Social and Behavioral Sciences, Harvard T.H. Chan School of Public Health, Boston, Massachusetts; 2 Division of Health Policy and Management, College of Health Sciences, Korea University, Seoul, South Korea; 3 Department of Public Health Sciences, Graduate School, Korea University, Seoul, South Korea; 4 Harvard Center for Population and Development Studies, Cambridge, Massachusetts; 5 Department of Economics and Centre for Modern Indian Studies, University of Goettingen, Germany

## Abstract

**Importance:**

Evidence on the relative importance of various factors associated with child anthropometric failures (ie, stunting, underweight, and wasting) and their heterogeneity across countries can inform global and national health agendas.

**Objective:**

To assess the relative significance of factors associated with child anthropometric failures in 35 low- and middle-income countries (LMICs).

**Design, Setting, and Participants:**

This cross-sectional study of 299 353 children who were born singleton and aged 12 to 59 months with nonpregnant mothers and valid anthropometric measures assessed the strengths of associations of 26 factors with child stunting, underweight, and wasting, using Demographic and Health Surveys (2007-2018) from 35 LMICs. Data analysis was conducted from July 2019 to February 2020.

**Exposures:**

A total of 9 direct factors (ie, dietary diversity score; breastfeeding initiation; vitamin A supplements; use of iodized salt; infectious disease in past 2 weeks; oral rehydration therapy for children with diarrhea; care seeking for suspected pneumonia; full vaccination; and indoor pollution) and 17 indirect factors (household wealth; maternal and paternal education; maternal and paternal height and body mass index; maternal autonomy for health care, movement, and money; water source; sanitation facility; stool disposal; antenatal care; skilled birth attendant at delivery; family planning needs; and maternal marriage age) were assessed.

**Main Outcomes and Measures:**

Three anthropometric failure outcomes were constructed based on the 2006 World Health Organization child growth standards: stunting (height-for-age *z* score less than −2 standard deviations [SDs]), underweight (weight-for-age *z* score less than −2 SDs), and wasting (weight-for-height *z* score less than −2 SDs).

**Results:**

Among the 299 353 children aged 12 to 59 months included in the analysis, 38.8% (95% CI, 38.6%-38.9%) had stunting, 27.5% (95% CI, 27.3%-27.6%) had underweight, and 12.9% (95% CI, 12.8%-13.0%) had wasting. In the pooled sample, short maternal height was the strongest factor associated with child stunting (odds ratio [OR], 4.7; 95% CI, 4.5-5.0; *P* < .001), followed by lack of maternal education (OR, 1.9; 95% CI, 1.8-2.0; *P* < .001), poorest household wealth (OR, 1.7; 95% CI,1.6-1.8; *P* < .001), and low maternal body mass index (OR, 1.6; 95% CI, 1.6-1.7; *P* < .001). Short paternal height was also significantly associated with higher odds of stunting (OR, 1.9; 95% CI, 1.7-2.2; *P* < .001). Consistent results were found for underweight (eg, short maternal height: OR, 3.5; 95% CI, 3.3-3.7; *P* < .001; lack of maternal education: OR, 1.8; 95% CI, 1.7-2.0; *P* < .001) and wasting (eg, low maternal body mass index: OR, 2.3; 95% CI, 2.1-2,4; *P* < .001; poorest household wealth: OR, 1.2; 95% CI, 1.1-1.3; *P* < .001). Parental nutritional status and household socioeconomic conditions ranked the strongest (1st to 4th) for most countries, with a few exceptions (eg, lack of maternal education ranked 18th-20th in 8 countries for child wasting). Other factors were not associated with anthropometric failures in pooled analysis and had large country-level heterogeneity; for example, unsafe water was not associated with child underweight in the pooled analysis (OR, 0.97; 95% CI, 0.95-1.00; *P* < .001), and it ranked from 4th to 20th across countries.

**Conclusions and Relevance:**

In this study, socioeconomic conditions and parental nutritional status were the strongest factors associated with child anthropometric failures. Poverty reduction, women’s education, and nutrition programs for households could be important strategies for reducing child undernutrition; however, country-specific contexts should be considered in national policy discussions.

## Key Points

**Question:**

What are the most important factors associated with child undernutrition, and how do they vary across countries?

**Findings:**

In this cross-sectional study of 299 353 children aged 12 to 59 months in 35 low- and middle-income countries, household socioeconomic status and parental nutritional status were the leading factors associated with child undernutrition in pooled analyses and in most country-specific analyses. Environmental conditions, health behaviors, disease prevalence, and maternal reproductive care were less frequently associated with child undernutrition, with substantial heterogeneity among countries.

**Meaning:**

The findings of this study suggest that interventions to improve socioeconomic status and parental nutritional status (eg, education for women and poverty reduction) should accompany food and nutrition programs, but the potential benefits of investing in specific conditions are highly dependent on the context.

## Teaser

This cross-sectional study assesses the relative significance of factors associated with child anthropometric failures for 35 low- and middle-income countries.

## Introduction

The global burden of child undernutrition remains high by all measures of child anthropometric failures (including stunting, underweight, and wasting).^1,2^ In 2018, 21.9% of children (ie, 149 million children) were estimated to have stunting.^1^ Immediate actions are needed to meet Sustainable Development Goal 2, ie, to end all forms of malnutrition by 2030,^3^ which in turn can contribute to other targets associated with child survival, educational achievements, and overall well-being. Several conceptual models have been developed to understand the causes of child undernutrition, most of which adopt multifactorial framework.^4,5^ The United Nations Children’s Fund (UNICEF) framework outlines socioeconomic conditions and national and global contexts as the fundamental factors affecting food security, care for children, and healthy household environment, all of which in turn further shape dietary intake, disease occurrence and, consequently, children’s nutritional status and growth.^5^ However, the UNICEF framework does not explicitly account for the role of parental nutritional status (eg, height and body mass index [BMI], calculated as weight in kilograms divided by height in meters squared), which may have intergenerational associations via biologic (eg, genetic disposition) and psychosocial (eg, poor living conditions) channels.^6^

Randomized clinical trials on child undernutrition tend to focus on a single factor or a small subset of them, making it difficult to infer their importance relative to other known factors.^7,8^ Some observational studies have attempted to simultaneously assess the association of multiple factors with child anthropometric failures in India,^9,10^ Rwanda,^11^ Bhutan,^12^ Bangladesh,^13^ and Nigeria,^14^ but the results are not directly comparable across countries given the different sets of factors considered in each study. There are only 2 multicountry studies, both focused on South Asia,^15,16^ which means the cross-country heterogeneity of the relative significance of factors associated with child undernutrition has been underexplored in other regions. While a 2017 multicountry meta-analysis^17^ identified fetal growth restriction and unimproved sanitation as the leading risk factors for child stunting, this study did not fully account for socioeconomic factors, such as household wealth and parental education.

Evidence regarding the relative strengths of factors associated with child anthropometric failures and their variation across countries is critical for understanding the underlying mechanisms of child undernutrition and potential context-specific interactions. Using the most recent data from the Demographic and Health Survey (DHS), we selected a comprehensive set of factors associated with child anthropometric failures and conducted a systematic analysis to assess their relative significance in 35 low- and middle-income countries (LMICs). In addition to pooled analyses, we present country-specific findings to inform the core intervention components needed to reduce child undernutrition in each country.

## Methods

### Data Source

We drew the most recent data for LMICs from DHSs conducted between 2007 and 2018. Demographic and Household Surveys are nationally representative household surveys that collect detailed nutrition and health information on children, their parents, and households^18^ using a multistage, stratified sampling design. The first stage involves the division of each country in geographic areas. Within these subnational regions, populations are stratified by urban or rural area. These primary sampling units or clusters are selected with probability proportional to the contribution of that cluster’s population to the total population. In the second stage of sampling, all households within the cluster are listed, and an average of 25 houses are randomly selected for an interview by equal-probability systematic sampling.^18^ We excluded earlier survey rounds to avoid inconsistencies in the measurements, collection, and reporting of data required for this study. The study was reviewed by the Harvard T.H. Chan School of Public Health institutional review board and was considered exempt from full review because it was based on an anonymous, public-use data set with no identifiable information on study participants. Our study followed the Strengthening the Reporting of Observational Studies in Epidemiology (STROBE) reporting guideline.

### Study Population and Sampling Size

A total of 35 LMICs had collected data on child anthropometric measures and the factors of interest. The eligibility criteria for our analytic sample were as follows: children (1) who born singleton, (2) who were aged 12 to 59 months and alive at the time of the survey, (3) with a mother who was not pregnant at the time of survey, and (4) with valid measures on child stunting, underweight, and wasting. We identified 299 353 children from 35 LMICs in the final analytic sample for our primary analysis (eFigure 1 in the Supplement).

### Outcomes

The following 3 anthropometric failure outcomes were constructed based on the 2006 World Health Organization child growth standards: stunting, underweight, and wasting.^19^ Height-for-age *z* score, weight-for-age *z* score, and weight-for-height *z* score were calculated by comparing the child’s measurements with the median value in the reference population of the National Center for Health Statistics International Growth Reference.^20^ Stunting was defined as a height-for-age *z *score less than −2 standard deviations (SDs) of the median, underweight as weight-for-age *z *score of less than −2 SDs, and wasting as weight-for-height *z *score less than −2 SDs.^19^

### Exposures

Based on the UNICEF framework,^5^ its adaption in the *Lancet* Maternal and Child Nutrition Series,^4^ and previous practices,^9,10,16^ we selected 20 factors for our primary analysis and 6 additional factors on paternal characteristics and maternal autonomy for supplementary analyses. We classified these 26 factors associated with child anthropometric failures either directly or via intermediary causes. A total of 9 direct factors were identified, including child nutrition (dietary diversity score, breastfeeding initiation, vitamin A supplements, and use of iodized salt), disease occurrence (infectious disease in past 2 weeks), health behaviors (oral rehydration therapy for diarrhea, care seeking for suspected pneumonia, full vaccination), and living conditions (indoor pollution). The association between each of these direct factors and child anthropometric failures has been documented previously.^17,21,22^ The remaining 17 indirect factors included household socioeconomic status (household wealth, maternal and paternal education), parents’ nutritional status (maternal and paternal height and BMI), maternal autonomy (for health care, movement, and money), environmental conditions (water source, sanitation facility, and stool disposal), maternal reproductive care (antenatal care, skilled birth attendant at delivery, family planning needs), and maternal marriage age. Prior studies have indicated that household wealth, maternal characteristics, and household environment are strongly associated with child anthropometric failures.^8,23,24^ Although only a few studies have investigated the role of paternal nutritional status, we included it in the supplementary analysis owing to potential biological and psychosocial channels between fathers and their offspring.^6,25^ We also included maternal reproductive care variables that represent the care mothers received during pregnancy,^26^ the risk the child faced during birth,^27^ and the families’ desired birth spacing and their capacity to reach it.^28^ A detailed list and definitions of these factors are presented in Table 1.^9,29,30,31,32,33,34,35,36^

**Table 1.** Definition of 26 Direct and Indirect Factors Associated With Child Anthropometric Failures Identified From a Comprehensive Review of Conceptual Framework and Prior Studies

Factor	Definition	Reference category	Self-reported
Dietary diversity score^9^	In quintiles, based on a score ranging from 0 to 8, with a point assigned for consuming grains, roots and tubers, legumes and nuts, dairy products (ie, milk, yogurt, cheese), flesh foods (ie, meat, fish, poultry, and liver or organ meats), eggs, vitamin-A rich fruits, and vegetables as per the 24-hour recall of food intake in the DHS; 1 indicates poor dietary diversity; 2, relatively poor; 3, middle; 4, relatively rich; 5, rich dietary diversity	Rich dietary diversity	Yes
Breastfeeding initiation^9^	In the 2 following categories: (1) initiation of breastfeeding within 1 h of birth and (2) initiation of breastfeeding >1 h of birth, defined as delayed breastfeeding	Breastfeeding initiation <1 h of birth	Yes
Full vaccination^29^	Yes if the child received a Bacillus Calmette–Guérin vaccination against tuberculosis; 3 doses of diptheria, pertussis, and tetanus vaccine; ≥3 doses of polio vaccine; and 1 dose of measles vaccine; no otherwise	Fully vaccinated	Mother’s self-report and vaccination cards
Vitamin A supplement^30^	Yes if the child received vitamin A supplementation; no otherwise	Received vitamin A supplement	No
Iodized salt^30^	Yes if iodized salt is used; no otherwise	Used iodized salt	No
Infectious disease in past 2 weeks^9^	Yes if the child had a history infectious disease (diarrhea, cough, or fever) in the 2 weeks before the survey; no otherwise	No infectious disease in the past 2 weeks	Yes
Oral rehydration therapy for children diarrhea^30^	In the 3 following categories: (1) no diarrhea, (2) diarrhea with oral rehydration therapy, and (3) diarrhea with no oral rehydration therapy	No diarrhea	Yes
Care seeking for suspected pneumonia^30^	In the 3 following categories: (1) no suspected pneumonia, (2) suspected pneumonia and sought for care for it, and (3) suspected pneumonia but did not seek for care for it.	No suspected pneumonia	Yes
Indoor pollution^9^	Low if the household used solid fuels for cooking; high otherwise	Low indoor pollution	Yes
Household wealth^31^	In quintiles, constructed by DHS based on a selected set of household assets; 1 indicates poorest household wealth; 2, poorer; 3, middle; 4, richer; 5, richest household wealth	Richest household wealth	No
Maternal education^32^	In the 4 following categories: (1) no schooling, (2) primary education, (3) secondary education, and (4) ≥college; no schooling was defined as lack of maternal education	≥College	Yes
Paternal education^32^	In the 4 following categories: (1) no schooling, (2) primary education, (3) secondary education, and (4) ≥college; no schooling was defined as lack of paternal education	≥College	Yes
Maternal height^33^	In the 5 following categories: (1) <145 cm, (2) 145-149.9 cm, (3) 150-154.9 cm, (4) 155-159.9 cm, and (5) ≥160 cm, with <145 cm defined as short maternal height	≥160 cm	No
Maternal BMI^34^	In the 3 following categories: (1) <18.5, (2) 18.5-24.9, and (3) ≥25, with <18.5 defined as low maternal BMI	≥25	No
Paternal height^9^	In the 5 following categories: (1) <155 cm, (2) 155-159.9 cm, (3) 160-164.9 cm, (4) 165-169.0 cm, and (5) ≥170, with <155 cm defined as short paternal height	≥170 cm	No
Paternal BMI^34^	In the 3 following categories: (1) <18.5, (2) 18.5-24.9, and (3) ≥25, with <18.5 defined as low paternal BMI	≥25	No
Drinking water source^35^	Safe if the household had access to water piped into dwelling, yard, or plot, public tap or standpipe, tube well or borehole, protected well or spring, rain water, and bottled water; unsafe otherwise	Safe water source	Yes
Sanitary facility^35^	Improved if the household had access to flush to piped sewer system, septic tank or pit latrine, ventilated improved pit latrine, pit latrine with slab, and composting toilet; unimproved otherwise	Improved sanitary facility	Yes
Stool disposal^36^	Safe if the child used a toilet or latrine, if fecal matter was put or rinsed into a toilet or latrine or buried; unsafe otherwise	Safe stool disposal	Yes
Antenatal care^30^	In the 3 following categories: (1) ≥8 antenatal care visits, (2) 4-7 antenatal care visits, and (3) <4 antenatal care visits from a skilled provider for the most recent birth	≥8 antenatal care visits	Yes
Skilled birth attendant at delivery^30^	Yes if a woman delivered the child with skilled birth attendant, including physicians, nurses, and midwives; no otherwise	Delivered the child with skilled birth attendant	Yes
Family planning need^30^	Satisfied if the woman, who was fecund and married or in a union, wishes to stop or delay childbearing or is currently using any modern method of contraception; unsatisfied otherwise	Family planning need satisfied	Yes
Maternal marriage age^30^	In the 2 following categories: (1) married at <18 y and (2) married at ≥18 y, with married at <18 y defined as child marriage	Married at <18 y	Yes
Woman has health care autonomy^30^	Yes if the mother was involved independently or jointly in the decision-making; no if the decision was made by the husband or partner or someone else.	The decision was made by the husband or partner or someone else	Yes
Woman has movement autonomy^30^	Yes if the mother was involved independently or jointly in the decision-making; no if the decision was made by the husband or partner or someone else	The decision was made by the husband or partner or someone else	Yes
Woman has money autonomy^30^	Yes if the mother was involved independently or jointly in the decision-making; no if the decision was made by the husband or partner or someone else.	The decision was made by the husband or partner or someone else	Yes

Abbreviation: BMI, body mass index (calculated as weight in kilograms divided by height in meters squared).

### Statistical Analysis

We assessed the association of each factor with child anthropometric outcomes by first pooling data from all countries and then separately for each country. We included sampling weight, clustering, and stratification variables provided by DHS to ensure that the estimates were representative at the national level and in pooled analyses.^16^ We clustered the sample at the level of the primary sampling unit, which allows for interdependence of error terms within clusters and households.^16^ In pooled analyses, we reweighted observations by a country’s population size and included country fixed effects to account for the unobservable country-level factors. For both pooled and country-specific analyses, we developed 2 sets of logistic regression models for each outcome. First, we ran separate models (single-adjusted models) for each factor in which we adjusted for child’s age and sex, birth order, and maternal age at birth. Second, we performed mutually adjusted models (fully adjusted models) in which all factors, as well as child’s age and sex, birth order, maternal age at birth, and place of residence (urban vs rural), were considered simultaneously. Based on these models, we compared and ordered the factors according to their coefficient sizes (odds ratios [ORs]). For all factors, the best-off group was set as the reference category to ensure consistency in interpretation of ORs. For factors with multiple categories (ie, household wealth quintile), only the OR corresponding the worst-off group (ie, the poorest quintile) is presented in our results section.

We performed 6 sets of supplementary analyses. First, we included 3 additional paternal characteristics for a subset of 188 290 children from 12 countries that had collected data on fathers. Second, we stratified children by age (<2 years and ≥2 years) given their different dietary demands.^37^ Third, we performed stratified analyses by urban and rural areas. For the second and third analyses, we followed previous practice^38^ and used Bonferroni correction to deal with the type I error from multiple testing. Fourth, we reestimated the fully adjusted models after removing source of drinking water, sanitation facility, and household air quality because these indicators had been considered in the construction of household wealth index in DHS. Fifth, we reran the models, adding covariates on children’s birth weight and birth interval. As more than half of the children (170 451 of 299 353 [56.9%]) had missing or invalid birth weight or birth interval, only on a subset of 128 902 children was used for this supplementary analysis. Sixth, we added 3 indicators of maternal autonomy for a subset of 142 638 children (47.6%) with available data.

We used Stata version 14.2 (StataCorp) for all analyses. We adopted the MI command for multiple imputations for observations with missing value on 1 or more factors of interest.^39,40^ All statistical tests were 2-tailed, and *P* < .05 was considered statistically significant.

## Results

Of 319 566 children who met the inclusion criteria, 20 213 (9.3%) were excluded because of missing (absent, refused, and missing for other reasons) or implausible anthropometric measures. A total of 299 353 children aged 12 to 59 months from 35 LMICs were included in the primary analysis (eFigure 1 in the Supplement). A total of 154 412 (51.6%) were boys, and 218 006 (72.8%) lived in rural areas. Overall, 38.8% (95% CI, 38.6%-38.9%) of children had stunting, 27.5% (95% CI, 27.3%-27.6%) had underweight, and 12.9% (95% CI, 12.8%-13.0%) had wasting (Table 2). The prevalence of anthropometric failures varied among countries, from 18.8% (95% CI, 17.9%-19.8%) in Peru to 61.1% (95% CI, 59.6%-62.6%) in Burundi for stunting, 2.9% (95% CI, 2.3%-3.5%) in Kyrgyzstan to 37.5% (95% CI, 35.8%-39.2%) in Niger for underweight, and 0.6% (95% CI, 0.4%-0.8%) in Peru to 19.0% (95% CI, 18.8%-19.2%) in India for wasting (eTable 1 in the Supplement). Overall, the burden of child anthropometric failures was higher in poorer households (eg, stunting among children with lowest vs highest wealth quintile, 51.2% [95% CI, 50.8%-51.5%] vs 22.3% [95% CI, 21.9%-22.7%]), and those with mothers who were less educated (eg, underweight among children whose mothers had no schooling vs ≥college education, 35.9% [95% CI, 35.6%-36.2%] vs 14.4% [95% CI, 13.9%-14.8%]), had shorter height (eg, wasting among children whose mothers were <145 cm vs ≥160 cm, 18.3% [95% CI, 17.8%-18.8%] vs 8.8% [95% CI, 8.5%-9.0%]), and had lower BMI (eg, stunting among children with mothers with BMI <18.5 vs ≥25.0, 49.7% [95% CI, 49.3%-50.1%] vs 26.4% [95% CI, 26.1%-26.8%]) (Table 2).

**Table 2.** Distribution of Child Anthropometric Failures by Selected Factors Among Children Aged 12 to 59 Months, Using the Most Recent Demographic Health Surveys Pooled Across 35 or 12 LMICs

Factor	Children observed, No. (%)	Prevalence, % (95% CI)		
		Stunting	Underweight	Wasting
Total sample for primary analysis across 35 LMICs	299 353 (100)	38.8 (38.6 to 38.9)	27.5 (27.3 to 27.6)	12.9 (12.8 to 13.0)
Child’s age, mo				
12-23	76 862 (25.7)	38.0 (37.7 to 38.4)	25.7 (25.4 to 26.0)	15.4 (15.2 to 15.7)
24-35	72 643 (24.3)	41.1 (40.7 to 41.4)	27.7 (27.4 to 28.0)	12.6 (12.4 to 12.9)
36-47	76 226 (25.5)	40.0 (39.6 to 40.3)	28.0 (27.7 to 28.3)	11.6 (11.4 to 11.9)
48-59	73 622 (24.6)	36.0 (35.6 to 36.3)	28.5 (28.2 to 28.8)	11.8 (11.6 to 12.0)
Child’s sex				
Male	154 412 (51.6)	37.6 (37.3 to 37.8)	26.8 (26.5 to 27.0)	12.0 (11.8 to 12.1)
Female	144 941 (48.4)	39.9 (39.6 to 40.1)	28.1 (27.9 to 28.4)	13.8 (13.6 to 13.9)
Type of residence				
Urban	81 347 (27.2)	29.5 (29.1 to 29.8)	20.9 (20.6 to 21.1)	11.7 (11.5 to 11.9)
Rural	218 006 (72.8)	42.6 (42.4 to 42.8)	30.2 (30.0 to 30.4)	13.4 (13.2 to 13.5)
Dietary diversity score, quintile				
1, worst	44 097 (14.7)	44.4 (43.9 to 44.9)	31.6 (31.2 to 32.0)	13.5 (13.1 to 13.8)
2	28 368 (9.5)	42.1 (41.6 to 42.7)	31.3 (30.7 to 31.8)	15.7 (15.3 to 16.1)
3	31 083 (10.4)	42.0 (41.4 to 42.5)	27.5 (27.0 to 28.0)	12.9 (12.5 to 13.3)
4	23 264 (7.8)	38.7 (38.0 to 39.3)	23.4 (22.9 to 24.0)	11.5 (11.1 to 11.9)
5, best	28 068 (9.4)	32.5 (32.0 to 33.1)	18.8 (18.4 to 19.3)	10.1 (9.8 to 10.5)
Missing	144 473 (48.3)	36.9 (36.7 to 37.2)	27.8 (27.6 to 28.0)	12.9 (12.8 to 13.1)
Breastfeeding initiation				
≥1 h of birth	135 569 (45.3)	37.8 (37.6 to 38.1)	25.7 (25.5 to 25.9)	12.1 (12.0 to 12.3)
<1 h of birth	145 896 (48.7)	40.3 (40.0 to 40.6)	30.0 (29.8 to 30.3)	14.0 (13.9 to 14.2)
Missing	17 888 (6.0)	34.2 (33.5 to 34.9)	22.0 (21.4 to 22.6)	10.1 (9.6 to 10.5)
Full vaccination				
No	108 593 (36.3)	37.0 (36.8 to 37.3)	26.4 (26.2 to 26.6)	13.4 (13.2 to 13.5)
Yes	163 836 (54.7)	42.5 (42.2 to 42.8)	31.9 (31.6 to 32.2)	14.5 (14.3 to 14.7)
Missing	26 924 (9.0)	34.6 (34.0 to 35.2)	16.8 (16.3 to 17.2)	4.2 (4.0 to 4.4)
Vitamin A supplement				
No	82 369 (27.5)	38.2 (38.0 to 38.4)	27.7 (27.5 to 27.9)	13.9 (13.7 to 14.0)
Yes	189 228 (63.2)	41.6 (41.3 to 42.0)	30.7 (30.4 to 31.0)	13.6 (13.3 to 13.8)
Missing	27 756 (9.3)	34.7 (34.2 to 35.3)	17.1 (16.7 to 17.6)	4.6 (4.4 to 4.8)
Iodized salt				
Not used	30 308 (10.1)	38.5 (38.4 to 38.7)	27.9 (27.7 to 28.0)	13.2 (13.1 to 13.4)
Used	261 381 (87.3)	40.3 (39.8 to 40.9)	26.2 (25.7 to 26.7)	11.5 (11.1 to 11.9)
Missing	7664 (2.6)	39.7 (38.6 to 40.8)	19.1 (18.2 to 19.9)	7.1 (6.5 to 7.7)
Infectious disease in past 2 wk				
No	210 648 (70.4)	38.7 (38.4 to 39.0)	25.0 (24.7 to 25.2)	11.0 (10.8 to 11.2)
Yes	88 091 (29.4)	38.8 (38.6 to 39.0)	28.5 (28.3 to 28.7)	13.7 (13.6 to 13.9)
Missing	614 (0.2)	41.0 (37.1 to 44.9)	25.6 (22.1 to 29.1)	8.9 (6.7 to 11.2)
ORT for child’s diarrhea				
No diarrhea	265 822 (88.8)	38.4 (38.2 to 38.5)	27.3 (27.2 to 27.5)	12.9 (12.8 to 13.0)
Had diarrhea with ORT	18 486 (6.2)	41.1 (40.3 to 41.8)	27.7 (27.0 to 28.3)	13.4 (12.9 to 13.9)
Had diarrhea without ORT	14 457 (4.8)	43.0 (42.2 to 43.8)	29.5 (28.8 to 30.3)	12.3 (11.8 to 12.9)
Missing	588 (0.2)	40.2 (36.2 to 44.2)	23.2 (19.7 to 26.6)	7.8 (5.6 to 10.0)
Care seeking for suspected pneumonia				
No suspected pneumonia	248 244 (82.9)	39.1 (39.0 to 39.3)	28.4 (28.3 to 28.6)	13.6 (13.4 to 13.7)
Had suspected pneumonia and sought care	24 419 (8.2)	35.2 (34.6 to 35.8)	24.1 (23.6 to 24.7)	11.7 (11.3 to 12.1)
Had suspected pneumonia and did not seek care	13 536 (4.5)	41.2 (40.4 to 42.1)	27.4 (26.6 to 28.1)	10.8 (10.3 to 11.3)
Missing	13 154 (4.4)	35.7 (34.9 to 36.6)	16.0 (15.4 to 16.6)	4.8 (4.5 to 5.2)
Maternal height, cm				
<145	21 278 (7.1)	62.2 (61.6 to 62.9)	49.6 (48.9 to 50.3)	18.3 (17.8 to 18.8)
145-149.9	53 523 (17.9)	49.8 (49.3 to 50.2)	38.9 (38.5 to 39.3)	17.1 (16.8 to 17.5)
150-154.9	80 791 (27.0)	40.3 (40.0 to 40.6)	29.0 (28.7 to 29.3)	14.4 (14.2 to 14.6)
155-159.9	68 457 (22.9)	33.3 (32.9 to 33.6)	21.4 (21.1 to 21.7)	11.5 (11.3 to 11.8)
≥160	58 283 (19.5)	26.9 (26.6 to 27.3)	15.9 (15.6 to 16.2)	8.8 (8.5 to 9.0)
Missing	17 021 (5.7)	31.1 (30.4 to 31.8)	15.5 (15.0 to 16.1)	5.9 (5.6 to 6.3)
Maternal BMI				
<18.5	52 260 (17.5)	49.7 (49.3 to 50.1)	46.2 (45.8 to 46.6)	22.1 (21.7 to 22.5)
18.5-24.9	177 165 (59.2)	40.0 (39.8 to 40.2)	26.8 (26.6 to 27.0)	12.7 (12.5 to 12.9)
≥25.0	52 607 (17.6	26.4 (26.1 to 26.8)	13.4 (13.1 to 13.7)	6.9 (6.6 to 7.1)
Missing	17 321 (5.8)	31.3 (30.6 to 32.0)	15.8 (15.3 to 16.4)	6.1 (5.8 to 6.5)
Indoor pollution				
Low	67 304 (22.5)	42.7 (42.5 to 42.9)	29.3 (29.1 to 29.5)	12.6 (12.5 to 12.8)
High	231 916 (77.5)	27.4 (27.0 to 27.7)	22.1 (21.8 to 22.4)	13.7 (13.4 to 14.0)
Missing	133 (0.0)	25.7 (18.1 to 33.2)	17.2 (10.7 to 23.7)	10.0 (4.8 to 15.2)
Household wealth quintile				
1, poorest	75 911 (25.4)	51.2 (50.8 to 51.5)	37.8 (37.4 to 38.1)	15.4 (15.1 to 15.6)
2	67 205 (22.5)	44.2 (43.8 to 44.5)	31.4 (31.0 to 31.7)	13.6 (13.3 to 13.8)
3	59 487 (19.9)	38.2 (37.8 to 38.6)	26.3 (25.9 to 26.6)	12.3 (12.1 to 12.6)
4	52 816 (17.6)	31.2 (30.8 to 31.6)	21.5 (21.2 to 21.9)	11.5 (11.3 to 11.8)
5, richest	43 934 (14.7)	22.3 (21.9 to 22.7)	15.1 (14.7 to 15.4)	10.6 (10.3 to 10.9)
Maternal education				
No schooling	100 154 (33.5)	47.9 (47.6 to 48.2)	35.9 (35.6 to 36.2)	15.3 (15.1 to 15.5)
Primary	71 447 (23.9)	41.1 (40.8 to 41.5)	23.7 (23.4 to 24.0)	9.6 (9.4 to 9.8)
Secondary	105 930 (35.4)	32.5 (32.2 to 32.8)	23.8 (23.6 to 24.1)	13.4 (13.2 to 13.6)
≥College	21 805 (7.3)	19.7 (19.2 to 20.2)	14.4 (13.9 to 14.8)	11.0 (10.6 to 11.4)
Missing	17 (0.0)	29.4 (5.3 to 53.6)	0.0 (0.0 to 0.0)	0.0 (0.0 to 0.0)
Drinking water source				
Unsafe	63 639 (21.3)	42.0 (41.6 to 42.4)	23.9 (23.5 to 24.2)	9.7 (9.4 to 9.9)
Safe	235 669 (78.7)	38.0 (37.8 to 38.2)	28.3 (28.2 to 28.5)	13.7 (13.5 to 13.8)
Missing	45 (0.0)	45.1 (30.0 to 60.2)	25.5 (12.2 to 38.7)	3.7 (−2.0 to 9.4)
Sanitary facility				
Not improved	162 646 (54.3)	45.5 (45.2 to 45.7)	32.1 (31.9 to 32.3)	13.6 (13.5 to 13.8)
Improved	136 624 (45.6)	30.7 (30.5 to 31.0)	21.9 (21.7 to 22.2)	12.0 (11.8 to 12.2)
Missing	83 (0.0)	34.9 (24.5 to 45.4)	27.2 (17.4 to 37.0)	4.6 (−0.0 to 9.1)
Stool disposal				
Unsafe	151 862 (50.7)	44.8 (44.5 to 45.0)	35.3 (35.1 to 35.5)	16.2 (16.0 to 16.3)
Safe	114 765 (38.3)	32.2 (31.9 to 32.5)	20.6 (20.4 to 20.8)	10.9 (10.8 to 11.1)
Missing	32 726 (10.9)	34.3 (33.8 to 34.9)	15.7 (15.3 to 16.1)	4.8 (4.6 to 5.1)
Antenatal care visits				
<4	98 791 (33.0)	44.5 (44.2 to 44.8)	32.6 (32.3 to 32.9)	15.2 (15.0 to 15.5)
4-7	70 531 (23.6)	32.6 (32.2 to 32.9)	22.1 (21.8 to 22.4)	12.2 (11.9 to 12.4)
≥8	26 923 (9.0)	27.5 (26.9 to 28.0)	21.4 (20.9 to 21.9)	14.4 (14.0 to 14.8)
Missing	103 108 (34.4)	41.3 (41.0 to 41.6)	28.3 (28.1 to 28.6)	10.8 (10.6 to 10.9)
Skilled birth attendant at delivery				
No	105 140 (35.1)	46.3 (46.0 to 46.6)	29.3 (29.0 to 29.6)	11.0 (10.8 to 11.2)
Yes	193 196 (64.5)	35.0 (34.8 to 35.2)	26.5 (26.3 to 26.7)	13.8 (13.7 to 14.0)
Missing	1017 (0.3)	44.7 (41.6 to 47.7)	32.7 (29.8 to 35.6)	8.9 (7.2 to 10.7)
Family planning needs satisfied				
No	82 866 (27.7)	40.8 (40.4 to 41.1)	28.8 (28.5 to 29.1)	12.9 (12.7 to 13.2)
Yes	207 185 (69.2)	38.3 (38.1 to 38.5)	27.5 (27.3 to 27.7)	13.1 (13.0 to 13.3)
Missing	9302 (3.1)	31.0 (30.0 to 31.9)	15.1 (14.4 to 15.9)	6.2 (5.7 to 6.7)
Maternal age at marriage, y				
<18	120 062 (40.1)	43.4 (43.2 to 43.7)	30.5 (30.2 to 30.8)	13.4 (13.2 to 13.5)
≥18	170 781 (57.1)	35.7 (35.5 to 35.9)	25.2 (25.0 to 25.4)	12.8 (12.7 to 13.0)
Missing	8510 (2.8)	36.5 (35.5 to 37.5)	20.3 (19.4 to 21.1)	9.1 (8.5 to 9.7)
Sample for supplementary analysis across 12 LMICs	188 290 (100)	40.5 (40.3-40.8)	34.0 (33.7-34.2)	17.2 (17.0-17.3)
Paternal height, cm				
<155	2524 (0.8)	54.0 (52.0 to 55.9)	46.4 (44.4 to 48.3)	23.7 (22.0 to 25.3)
155-159.9	5186 (1.7)	50.4 (49.1 to 51.8)	40.3 (38.9 to 41.6)	18.4 (17.3 to 19.4)
160-164.9	8627 (2.9)	42.9 (41.9 to 44.0)	31.5 (30.5 to 32.5)	15.0 (14.3 to 15.8)
165-169.9	9071 (3.0)	36.7 (35.7 to 37.6)	25.2 (24.3 to 26.1)	12.5 (11.8 to 13.1)
≥170	9735 (3.3)	27.0 (26.1 to 27.9)	17.0 (16.2 to 17.7)	9.5 (9.0 to 10.1)
Missing	153 147 (51.2)	40.9 (40.6 to 41.1)	34.2 (33.9 to 34.4)	17.7 (17.5 to 17.9)
Paternal BMI				
<18.5	5162 (1.7)	49.8 (48.5 to 51.2)	42.9 (41.6 to 44.3)	19.1 (18.0 to 20.2)
18.5-24.9	24 209 (8.1)	38.9 (38.3 to 39.5)	27.3 (26.8 to 27.9)	13.5 (13.1 to 13.9)
≥25.0	5756 (1.9)	28.4 (27.2 to 29.6)	18.7 (17.7 to 19.8)	11.3 (10.5 to 12.2)
Missing	153 163 (51.2)	40.9 (40.6 to 41.1)	34.2 (33.9 to 34.4)	17.7 (17.5 to 17.9)
Paternal education				
No schooling	8119 (2.7)	48.2 (47.1 to 49.3)	35.6 (34.5 to 36.6)	15.0 (14.3 to 15.8)
Primary	9027 (3.0)	41.5 (40.5 to 42.5)	27.6 (26.7 to 28.5)	12.4 (11.7 to 13.0)
Secondary	17 540 (5.9)	35.7 (35.0 to 36.4)	26.9 (26.3 to 27.6)	14.4 (13.9 to 14.9)
≥College	4127 (1.4)	25.0 (23.6 to 26.3)	18.5 (17.3 to 19.6)	12.6 (11.6 to 13.6)
Missing	149 477 (49.9)	41.0 (40.8 to 41.3)	34.4 (34.1 to 34.6)	17.8 (17.6 to 18.0)

Abbreviations: BMI, body mass index (calculated as weight in kilograms divided by height in meters squared); LMICs, low- and middle-income countries; ORT, oral rehydration therapy.

### Pooled Analyses

#### Stunting

**Figure 1.**
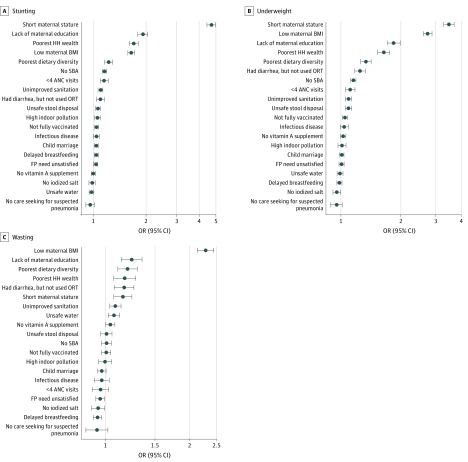
Relative Ranking of 20 Factors Associated With Child Anthropometric Failures From Fully Adjusted Models Short maternal statue indicates maternal height of less than 145 cm; low maternal body mass index (BMI, calculated as weight in kilograms divided by height in meters squared), BMI less than 18.5; child marriage, mother younger than 18 years at marriage; delayed breastfeeding, child was not breastfed within 1 hour of birth; infectious disease, child had infectious disease within 2 weeks before survey. ANC indicates antenatal care; FP, family planning; HH, household; OR, odds ratio; ORT, oral rehydration therapy; SBA, skilled birth attendant.

The full regression results from pooled analyses are presented in eTable 2 in the Supplement. In single-adjusted models, all factors except for lack of vitamin A supplement, history of infectious disease, and no iodized salt use were significantly associated with higher odds of stunting (eFigure 2A in the Supplement). Short maternal height showed the strongest association with child stunting (OR, 4.4; 95% CI, 4.2-4.6; *P* < .001), followed by lack of maternal education (OR, 3.5; 95% CI, 3.3-3.7; *P* < .001) and poorest household wealth (OR, 3.4; 95% CI, 3.2-3.5; *P* < .001). The magnitude of associations substantially attenuated for most factors in the fully adjusted model; however, 15 remained statistically significant (Figure 1A). Conditional on all other factors, short maternal height had the strongest association with child stunting, with an OR of 4.7 (95% CI, 4.5-5.0; *P* < .001), followed by lack of maternal education (OR, 1.9; 95% CI,1.8-2.0; *P* < .001), poorest household wealth (OR, 1.7; 95% CI,1.6-1.8; *P* < .001), and low maternal BMI (OR, 1.6; 95% CI,1.6-1.7; *P* < .001).

#### Underweight

In single-adjusted models, all factors were significantly associated with higher odds of underweight, except for no care seeking for suspected pneumonia, no vitamin A supplement, infectious disease during past 2 weeks, and unsafe water (eFigure 2B in the Supplement). Short maternal height had the strongest association with child underweight (OR, 5.3; 95% CI, 5.0-5.6; *P* < .001), followed by low maternal BMI (OR, 4.8; 95% CI, 4.6-5.0; *P* < .001) and poorest household wealth (OR, 3.4; 95% CI, 3.2-3.6; *P* < .001). In the fully adjusted model, we found 11 factors to be significantly associated with higher odds of underweight (Figure 1B), including short maternal height (OR, 3.5; 95% CI, 3.3-3.7; *P* < .001), low maternal BMI (OR, 2.7; 95% CI, 2.6-2.9; *P* < .001), lack of maternal education (OR, 1.8; 95% CI, 1.7-2.0; *P* < .001), and poorest household wealth (OR, 1.6; 95% CI,1.5-1.8; *P* < .001).

#### Wasting

In single-adjusted models, there were 10 factors significantly associated with higher odds of wasting, with short maternal height (OR, 4.4; 95% CI, 4.2-4.6; *P* < .001), lack of maternal education (OR, 3.5; 95% CI, 3.3-3.7; *P* < .001), and poorest household wealth (OR, 3.4 95% CI, 3.2-3.5; *P* < .001) having the largest magnitudes (eFigure 2C in the Supplement). The fully adjusted model showed consistent results in terms of the factors with the largest magnitudes, such as low maternal BMI (OR, 2.3; 95% CI, 2.1-2.4; *P* < .001), no maternal education (OR, 1.2; 95% CI, 1.1-1.4 ; *P* < .001), poor dietary diversity (OR, 1.2; 95% CI, 1.1-1.3), and poorest household wealth (OR, 1.2; 95% CI, 1.1-1.3; *P* < .001) (Figure 1C).

### Country-Specific Analyses

#### Stunting

**Figure 2.**
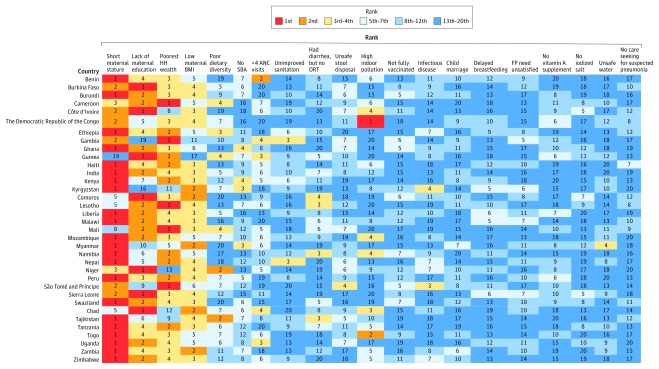
Country-Specific Ranking of 20 Factors Associated with Stunting Short maternal statue indicates maternal height of less than 145 cm; low maternal body mass index (BMI, calculated as weight in kilograms divided by height in meters squared), BMI less than 18.5; child marriage, mother younger than 18 years at marriage; delayed breastfeeding, child was not breastfed within 1 hour of birth; infectious disease, child had infectious disease within 2 weeks before survey. ANC indicates antenatal care; FP, family planning; HH, household; ORT, oral rehydration therapy; SBA, skilled birth attendant.

**Figure 3.**
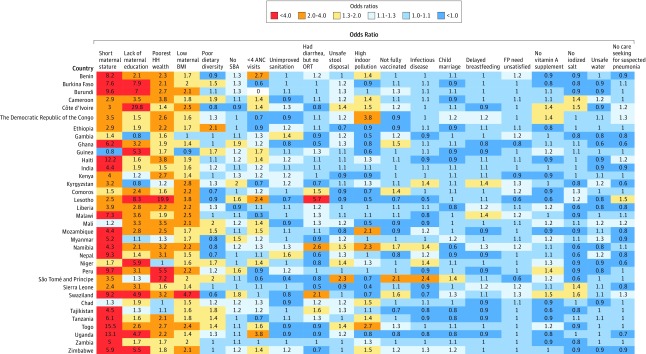
Country-Specific Odds Ratios for 20 Factors Associated With Child Anthropometric Failures From Fully Adjusted Models on Stunting Short maternal statue indicates maternal height of less than 145 cm; low maternal body mass index (BMI, calculated as weight in kilograms divided by height in meters squared), BMI less than 18.5; child marriage, mother younger than 18 years at marriage; delayed breastfeeding, child was not breastfed within 1 hour of birth; infectious disease, child had infectious disease within 2 weeks before survey. ANC indicates antenatal care; FP, family planning; HH, household; ORT, oral rehydration therapy; SBA, skilled birth attendant.

Short maternal height had the strongest association with stunting for all 35 countries, with ORs being ranked first in 22 countries and between second and fifth in 11 countries (Figure 2). Lack of maternal education, low maternal BMI, and poorest household wealth were also strongly associated with stunting for most countries. However, there were several exceptions. For example, lack of maternal education ranked 19th in Gambia. The ranking of other factors, such as unsafe sanitation, no skilled birth attendant at birth, and poor household air quality, varied largely across countries. The magnitudes of ORs for all factors were also heterogeneous (Figure 3). For example, the magnitudes of ORs for short maternal height ranged from 0.8 (95% CI, 0.3-2.4) in Guinea to 15.5 (95% CI, 3.5-97.1) in Togo; the magnitudes of ORs for poor household air quality ranged from 0.4 (95% CI, 0.0-3.5) in Sierra Leone to 3.8 (95% CI, 1.3-11.5) in Democratic Republic of the Congo.

#### Underweight

Short maternal height was most strongly associated with higher odds of underweight (ranked 1st-4th) in 29 countries; however, it ranked 20th in Namibia. Low maternal BMI was also strongly associated with underweight across all 35 countries, ranking between 1st and 6th. The relative rankings for lack of maternal education and poorest household wealth varied largely across countries. For example, poorest household wealth ranked 1st to 4th in 13 of 35 countries, while it ranked 10th or lower for 10 countries (eFigure 3 in the Supplement). All other factors showed great heterogeneity in their relative rankings across countries. We also observed substantial variations in the factors’ magnitudes across countries (eFigure 4 in the Supplement). For example, the magnitudes of ORs for lack of maternal education ranged from 0.7 (95% CI, 0.4-1.3) in Myanmar to 33.3 (95% CI, 2.3-483.9) in Lesotho; the magnitudes of ORs for no care seeking for suspected pneumonia ranged from 0.7 (95% CI, 0.3-1.2) in Comoros to 5.1 (95% CI, 2.0-13.2) in Namibia.

#### Wasting

Low maternal BMI ranked within the top 5 factors associated with wasting in most countries, except Comoros, Namibia, São Tomé and Príncipe, and Zambia. Short maternal height, poorest household wealth, and lack of maternal education were strongly associated with higher odds of child wasting for some countries but were found to have weaker associations in many other countries. For example, lack of maternal education ranked between 1st and 4th in 12 countries but ranked between 18th and 20th in 8 countries (eFigure 5 in the Supplement). The strength of association for each factor and child wasting also showed large variations across countries (eFigure 6 in the Supplement). For example, the magnitudes of ORs for low maternal BMI ranged from 1.2 (95% CI, 0.7-2.0) in Zambia to 40.0 (95% CI, 5.7-279.2) in Swaziland; the magnitudes of ORs for unimproved sanitation ranged from 0.6 (95% CI, 0.2-2.0) in Namibia to 16.8 (95% CI, 3.8-74.0) in Lesotho.

### Supplementary Analyses

In the first supplementary analysis with paternal height, BMI, and education, we found that paternal factors had weaker associations with child anthropometric failures compared with maternal indicators (eFigure 7 in the Supplement). Short paternal height was associated with stunting with an OR of 1.9 (95% CI, 1.7-2.2; *P* < .001) compared with an OR of 4.5 (95% CI, 4.2-4.8; *P* < .001) for short maternal height. Paternal anthropometry had statistically significant associations with all types of child anthropometric failure, although their rankings and magnitudes varied across countries. For example, low paternal BMI was significantly associated with stunting (OR, 1.2; 95%CI, 1.1-1.4; *P* < .001), underweight (OR, 1.5; 95%CI, 1.3-1.7; *P* < .001), and wasting (OR, 1.2; 95%CI, 1.1-1.5; *P* < .001) in pooled analysis; however, the magnitudes of low paternal BMI ranged widely, from 4th in Namibia (OR, 2.5; 95%CI, 0.8-7.8) to 23th in Swaziland (OR, 0.5; 95%CI, 0.1-2.1) for stunting, from 3rd in Zimbabwe (OR, 1.8; 95%CI, 0.8-4.0) to 20th in Sierra Leone (OR, 0.9; 95% CI, 0.5-1.8) for underweight, and from 1st in Nepal (OR, 2.9; 95% CI, 0.9-9.7) to 23rd in Lesotho (OR, 0.1; 95% CI, 0.0-4.9) for wasting. Lack of paternal education ranked low and had small effect sizes in most countries (eFigure 8 and eFigure 9 in the Supplement).

As second and third supplementary analyses, we stratified children by age and by urban and rural residence. We found consistent results for short maternal stature, lack of maternal education, poorest household wealth, and low maternal BMI, but moderate differences were observed for other factors across the stratified groups. For example, no oral rehydration therapy for diarrhea was not associated with child stunting among children younger than 2 years (OR, 1.04; 95% CI, 0.95-1.13), but it was associated with stunting among children aged 2 years and older (OR, 1.2; 95% CI, 1.1-1.3) (eFigure 10 in the Supplement). The results from country-specific stratified analyses are summarized in eFigure 11 to eFigure 15 in the Supplement.

In the fourth supplementary analysis, we excluded source of drinking water, sanitation facility, and household air quality from the fully adjusted models to avoid potential multicollinearity, and the rankings and magnitudes of all factors remained largely the same (eFigure 16, eFigure 17, and eFigure 18 in the Supplement). Moreover, we adopted variance inflation factor (VIF) to check for multicollinearity. For example, for the outcome of child stunting, the regression model including all factors had a VIF of less than 4 for all factors, except for the poorest quintile of household wealth index (VIF, 6.19) and no maternal education (VIF, 5.46). After removing source of drinking water, sanitation facility, and household air quality from the regression model, all VIFs reduced to less than 4, indicating relatively low multicollinearity.

The magnitude of the selected factors remained largely the same after additionally controlling for birth characteristics (ie, birth weight and preceding birth interval) in the fifth supplementary analysis (eTable 3 in the Supplement). Finally, indicators on women’s empowerment ranked low and had nonsignificant ORs for all 3 anthropometric failures (eFigure 19 in the Supplement).

## Discussion

Maternal nutritional status (height and BMI) and poor household socioeconomic conditions (household wealth and maternal education) were the leading factors associated with child anthropometric failures in our pooled analyses. Fathers’ nutritional status also appeared to be associated with child anthropometric status, but paternal education was not. Despite some exceptions, parental nutritional status and poor household socioeconomic conditions were the strongest factors in most countries. The relative significance and absolute magnitude of other factors, such as care-seeking behaviors, reproductive care, and air quality, showed considerable heterogeneity among countries.

A rich volume of observational studies supports our findings regarding maternal height and BMI,^8,16^ but paternal anthropometry remains largely unexplored. The associations between short parental height and child anthropometric status may be attributed to both shared genetic background and common environmental determinants (eg, diet, culture, social class) that first affect parents during their early childhood and subsequently affect the growth of their offspring.^41^ The consistent association between maternal BMI and child anthropometric failures may be attributed to intrauterine intergenerational transmission of low maternal BMI during pregnancy, giving infants a high risk of low birth weight and being small for gestational age, which forms the fetal origins of subsequent childhood undernutrition.^34,42^ While we did not have data on maternal BMI during pregnancy, BMI at the time of the survey is likely to be associated with previous weight. The influence of maternal BMI on child anthropometric status attenuated only moderately after adding paternal BMI.^34^

Our pooled estimates on household wealth and maternal education were comparable with previous multicountry studies.^7,43^ Across countries, household wealth had moderate heterogeneity in associations with child stunting and underweight. The relative importance of maternal education ranged from very high (eg, Côte d’Ivoire, Mali, Ghana) to low (eg, Gambia, Kyrgyzstan, Myanmar). Such heterogeneity may be partially explained by differences in macroeconomic status, health system, and the existence of national and local programs. For example, the relatively weaker association between socioeconomic conditions and child anthropometric failures in Kyrgyzstan may be explained by investments in primary care facilities and hospitals in disadvantaged areas.^44^

Children’s dietary diversity, oral rehydration therapy for diarrhea, and sanitation facilities were associated with all outcomes in the pooled analyses, but the results varied among countries. Country-level heterogeneity in the association between dietary diversity and child anthropometric failures has been documented in previous observational studies and randomized clinical trials, with a protective effect found in Mali^45^ and Bangladesh^46^ but not in Niger^47^ or Kenya.^48^ Different levels of food security and the existence of nutritional supplement programs (eg, Foodlets, Sprinkles, and lipid-based nutrient supplements) in some countries may explain the observed heterogeneity. Inconsistent findings on the association of oral rehydration therapy for diarrhea with outcomes may be because of the differential prevalence of children very close to the anthropometric failure cutoffs given that only they would be substantially affected by the occurrence of diarrhea and oral rehydration treatment.^49,50^ The heterogeneous association between sanitation facility and child undernutrition may be attributed to differences in complementarity of toilet maintenance, including other water and hygiene practices.^51,52^

### Limitations

There are several limitations to this study. First, factors in the fully adjusted models may be associated with each other and serve as confounders or mediators. Multicollinearity can increase the standard errors of the coefficients and weaken the significance levels, but it does not result in biased estimates. Moreover, the low VIF for all factors presented in the supplementary analysis section indicated low multicollinearity. Second, the use of observational data and cross-sectional analysis limit our capacity to make any causal inferences. Third, some factors analyzed in this study, such as breastfeeding history, care-seeking behavior, and disease history, were self-reported and, therefore, are prone to potential measurement errors.

## Conclusions

This systematic investigation of the comparative importance of direct and indirect factors associated with child anthropometric failures suggests the universal importance of improving maternal nutritional status and household socioeconomic circumstances. The relative importance of other factors was weaker and more heterogeneous among countries, suggesting the need for context-specific understanding to inform national policies and programs.

## Notes

Supplement. **eTable 1. **Percentage of Children Aged 12 to 59 Months Classified as Having Stunting, Underweight, or Wasting in 35 Countries **eTable 2. **Full Regression Results From the Pooled, Fully Adjusted Model **eTable 3. **Magnitudes of 20 Factors Associated With Child Anthropometric Failures From Fully Adjusted Models for the Pooled Sample **eFigure 1. **Flow Diagram Showing Exclusions and Final Sample Sizes of the Study Population, Using the Most Recent Pooled Demographic Health Survey Data Since 2010 **eFigure 2. **Relative Ranking of 20 Factors Associated With Child Anthropometric Failures From Single Adjusted Models for the Pooled Sample **eFigure 3. **Country-Specific Ranking of 20 Factors Associated With Child Anthropometric Failures From Fully Adjusted Models on Underweight **eFigure 4. **Country-Specific Odds Ratios for 20 Factors Associated With Child Anthropometric Failures From Fully Adjusted Models on Underweight **eFigure 5. **Country-Specific Ranking of 20 Factors Associated With Child Anthropometric Failures From Fully Adjusted Models on Wasting **eFigure 6. **Country-Specific Odds Ratios for 20 Factors Associated With Child Anthropometric Failures From Fully Adjusted Models on Wasting **eFigure 7. **Relative Ranking of 23 Factors Associated With Child Anthropometric Failures From Supplementary Analysis of Fully Adjusted Models for the Pooled Sample **eFigure 8. **Country-Specific Ranking of 23 Factors Associated With Child Anthropometric Failures From Supplementary Analysis of Fully Adjusted Models on Stunting, Underweight, and Wasting in 12 Countries **eFigure 9. **Country-Specific Odds Ratios for 23 Factors Associated With Child Anthropometric Failures From Supplementary Analysis of Fully Adjusted Models on Stunting, Underweight, and Wasting in 12 Countries **eFigure 10. **Relative Ranking of 20 Factors Associated With Child Anthropometric Failures From Fully Adjusted Models for Pooled Sample, Stratified by Children’s Age **eFigure 11. **Country-Specific Ranking of 20 Factors Associated With Child Anthropometric Failures From Fully Adjusted Models, Stratified by Children’s Age **eFigure 12. **Country-Specific Odds Ratios for 20 Factors Associated With Child Anthropometric Failures From Fully Adjusted Models, Stratified by Children’s Age **eFigure 13. **Relative Ranking of 20 Factors Associated With Child Anthropometric Failures From Fully Adjusted Models for Pooled Sample, Stratified by Place of Residence **eFigure 14. **Country-Specific Ranking of 20 Factors Associated With Child Anthropometric Failures From Fully Adjusted Models, Stratified by Place of Residence **eFigure 15. **Country-Specific Odds Ratios for 20 Factors Associated With Child Anthropometric Failures From Fully Adjusted Models, Stratified by Place of Residence **eFigure 16. **Relative Ranking of 17 Factors Associated With Child Anthropometric Failures From Fully Adjusted Models for the Pooled Sample, Excluding Source of Drinking Water, Sanitation Facility, and Household Air Quality **eFigure 17. **Country-Specific Ranking of 17 Factors Associated With Child Anthropometric Failures From Fully Adjusted Models for the Pooled Sample, Excluding Source of Drinking Water, Sanitation Facility, and Household Air Quality **eFigure 18. **Country-Specific Odds Ratios for 17 Factors Associated With Child Anthropometric Failures From Fully Adjusted Models for the Pooled Sample, Excluding Source of Drinking Water, Sanitation Facility, and Household Air Quality **eFigure 19. **Relative Ranking of 23 Factors Associated With Child Anthropometric Failures From Fully Adjusted Models for the Pooled Sample, Adding Women’s Empowerment Factors
