# Supplementary material for: Factors Associated With Child Stunting, Wasting, and Underweight in 35 Low- and Middle-Income Countries
Source: JAMA Netw Open. 2020 Apr 22;3(4):e203386. doi: 10.1001/jamanetworkopen.2020.3386 (PMC7177203; doi:10.1001/jamanetworkopen.2020.3386)
Supplement: Supplement. — eTable 1. Percentage of Children Aged 12 to 59 Months Classified as Having Stunting, Underweight, or Wasting in 35 Countries eTable 2. Full Regression Results From the Pooled, Fully Adjusted Model eTable 3. Magnitudes of 20 Factors Associated With Child Anthropometric Failures From Fully Adjusted Models for the Pooled Sample eFigure 1. Flow Diagram Showing Exclusions and Final Sample Sizes of the Study Population, Using the Most Recent Pooled Demographic Health Survey Data Since 2010 eFigure 2. Relative Ranking of 20 Factors Associated With Child Anthropometric Failures From Single Adjusted Models for the Pooled Sample eFigure 3. Country-Specific Ranking of 20 Factors Associated With Child Anthropometric Failures From Fully Adjusted Models on Underweight eFigure 4. Country-Specific Odds Ratios for 20 Factors Associated With Child Anthropometric Failures From Fully Adjusted Models on Underweight eFigure 5. Country-Specific Ranking of 20 Factors Associated With Child Anthropometric Failures From Fully Adjusted Models on Wasting eFigure 6. Country-Specific Odds Ratios for 20 Factors Associated With Child Anthropometric Failures From Fully Adjusted Models on Wasting eFigure 7. Relative Ranking of 23 Factors Associated With Child Anthropometric Failures From Supplementary Analysis of Fully Adjusted Models for the Pooled Sample eFigure 8. Country-Specific Ranking of 23 Factors Associated With Child Anthropometric Failures From Supplementary Analysis of Fully Adjusted Models on Stunting, Underweight, and Wasting in 12 Countries eFigure 9. Country-Specific Odds Ratios for 23 Factors Associated With Child Anthropometric Failures From Supplementary Analysis of Fully Adjusted Models on Stunting, Underweight, and Wasting in 12 Countries eFigure 10. Relative Ranking of 20 Factors Associated With Child Anthropometric Failures From Fully Adjusted Models for Pooled Sample, Stratified by Children’s Age eFigure 11. Country-Specific Ranking of 20 Factors Associated With Child Anthropo [file jamanetwopen-3-e203386-s001.pdf]

Supplementary Online Content

Li Z, Kim R, Vollmer S, Subramanian SV. Factors associated with child stunting, wasting, and underweight in 35 low- and middle-income countries. *JAMA Netw Open*. 2020;3(4):e203386. doi:10.1001/jamanetworkopen.2020.3386

eTable 1. Percentage of Children Aged 12 to 59 Months Classified as Having Stunting, Underweight, or Wasting in 35 Countries
eTable 2. Full Regression Results From the Pooled, Fully Adjusted Model
eTable 3. Magnitudes of 20 Factors Associated With Child Anthropometric Failures From Fully Adjusted Models for the Pooled Sample
eFigure 1. Flow Diagram Showing Exclusions and Final Sample Sizes of the Study Population, Using the Most Recent Pooled Demographic Health Survey Data Since 2010
eFigure 2. Relative Ranking of 20 Factors Associated With Child Anthropometric Failures From Single Adjusted Models for the Pooled Sample
eFigure 3. Country-Specific Ranking of 20 Factors Associated With Child Anthropometric Failures From Fully Adjusted Models on Underweight
eFigure 4. Country-Specific Odds Ratios for 20 Factors Associated With Child Anthropometric Failures From Fully Adjusted Models on Underweight
eFigure 5. Country-Specific Ranking of 20 Factors Associated With Child Anthropometric Failures From Fully Adjusted Models on Wasting
eFigure 6. Country-Specific Odds Ratios for 20 Factors Associated With Child Anthropometric Failures From Fully Adjusted Models on Wasting
eFigure 7. Relative Ranking of 23 Factors Associated With Child Anthropometric Failures From Supplementary Analysis of Fully Adjusted Models for the Pooled Sample
eFigure 8. Country-Specific Ranking of 23 Factors Associated With Child Anthropometric Failures From Supplementary Analysis of Fully Adjusted Models on Stunting, Underweight, and Wasting in 12 Countries
eFigure 9. Country-Specific Odds Ratios for 23 Factors Associated With Child Anthropometric Failures From Supplementary Analysis of Fully Adjusted Models on Stunting, Underweight, and Wasting in 12 Countries
eFigure 10. Relative Ranking of 20 Factors Associated With Child Anthropometric Failures From Fully Adjusted Models for Pooled Sample, Stratified by Children's Age
eFigure 11. Country-Specific Ranking of 20 Factors Associated With Child Anthropometric Failures From Fully Adjusted Models, Stratified by Children's Age
eFigure 12. Country-Specific Odds Ratios for 20 Factors Associated With Child Anthropometric Failures From Fully Adjusted Models, Stratified by Children's Age
eFigure 13. Relative Ranking of 20 Factors Associated With Child Anthropometric Failures From Fully Adjusted Models for Pooled Sample, Stratified by Place of Residence
eFigure 14. Country-Specific Ranking of 20 Factors Associated With Child Anthropometric Failures From Fully Adjusted Models, Stratified by Place of Residence

© 2020 Li Z et al. *JAMA Network Open.*

eFigure 15. Country-Specific Odds Ratios for 20 Factors Associated With Child Anthropometric Failures From Fully Adjusted Models, Stratified by Place of Residence
eFigure 16. Relative Ranking of 17 Factors Associated With Child Anthropometric Failures From Fully Adjusted Models for the Pooled Sample, Excluding Source of Drinking Water, Sanitation Facility, and Household Air Quality
eFigure 17. Country-Specific Ranking of 17 Factors Associated With Child Anthropometric Failures From Fully Adjusted Models for the Pooled Sample, Excluding Source of Drinking Water, Sanitation Facility, and Household Air Quality
eFigure 18. Country-Specific Odds Ratios for 17 Factors Associated With Child Anthropometric Failures From Fully Adjusted Models for the Pooled Sample, Excluding Source of Drinking Water, Sanitation Facility, and Household Air Quality
eFigure 19. Relative Ranking of 23 Factors Associated With Child Anthropometric Failures From Fully Adjusted Models for the Pooled Sample, Adding Women's Empowerment Factors

This supplementary material has been provided by the authors to give readers additional information about their work.

© 2020 Li Z et al. *JAMA Network Open.*

eTable 1. Percentage of Children Aged 12 to 59 Months Classified as Having Stunting, Underweight, or Wasting in 35 Countries

Country	Year	Stunting (%)	Underweight (%)	Wasting (%)
Benin	2018	34.6(33.6, 35.7)	15.8(15.0, 16.6)	3.9(3.5, 4.3)
Burkina Faso	2010	38.9(37.5, 40.4)	25.3(24.0, 26.6)	12.0(11.0, 13.0)
Burundi	2016	61.1(59.6, 62.6)	31.4(30.0, 32.8)	4.5(3.9, 5.2)
Cameroon	2011	36.5(34.8, 38.2)	15.0(13.8, 16.3)	4.7(4.0, 5.4)
Chad	2015	47.5(46.3, 48.6)	32.7(31.6, 33.8)	12.3(11.6, 13.1)
Comoros	2012	31.4(29.3, 33.5)	15.7(14.0, 17.4)	9.5(8.1, 10.8)
Cote d'Ivoire	2012	32.7(30.6, 34.7)	14.3(12.8, 15.8)	5.8(4.8, 6.8)
Democratic Republic of Congo	2013	48.3(47.0, 49.7)	24.2(23.0, 25.4)	6.3(5.6, 7.0)
Ethiopia	2016	44.4(43.1, 45.7)	25.0(23.9, 26.1)	8.7(8.0, 9.4)
Gambia	2013	26.3(24.3, 28.2)	16.5(14.9, 18.1)	10.4(9.1, 11.8)
Ghana	2014	19.9(18.1, 21.8)	10.5(9.1, 11.9)	3.6(2.7, 4.4)
Guinea	2018	33.3(31.3, 35.2)	15.5(14.0, 17.0)	8.1(7.0, 9.2)
Haiti	2017	21.8(20.5, 23.1)	8.1(7.2, 8.9)	2.5(2.0, 3.0)
India	2015	41.4(41.2, 41.7)	36.9(36.7, 37.2)	19.0(18.8, 19.2)
Kenya	2014	28.6(27.9, 29.4)	11.4(10.9, 12.0)	3.7(3.4, 4.1)
Kyrgyzstan	2012	19.5(18.0, 21.0)	2.9(2.3, 3.5)	2.0(1.5, 2.6)
Lesotho	2014	36.1(33.0, 39.2)	11.2(9.2, 13.2)	2.6(1.5, 3.6)
Liberia	2013	34.4(32.4, 36.3)	14.8(13.3, 16.2)	4.9(4.0, 5.8)
Malawi	2015	39.4(37.8, 41.0)	11.4(10.4, 12.4)	2.6(2.1, 3.1)
Mali	2018	30.1(28.9, 31.3)	18.9(17.9, 20.0)	7.9(7.3, 8.6)
Mozambique	2011	45.7(44.5, 47.0)	14.1(13.3, 15.0)	4.8(4.3, 5.4)
Myanmar	2016	32.1(30.5, 33.8)	20.1(18.7, 21.6)	6.4(5.6, 7.3)
Namibia	2013	25.2(22.7, 27.7)	14.3(12.3, 16.3)	5.6(4.3, 6.9)
Nepal	2016	40.7(38.4, 43.0)	29.2(27.1, 31.4)	8.4(7.1, 9.7)
Niger	2012	48.6(46.9, 50.4)	37.5(35.8, 39.2)	16.1(14.8, 17.4)
Peru	2012	18.8(17.9, 19.8)	3.2(2.8, 3.7)	0.6(0.4, 0.8)
São Tomé and Príncipe	2008	26.9(24.1, 29.6)	13.0(10.9, 15.0)	11.3(9.3, 13.2)
Sierra Leone	2013	41.0(39.2, 42.8)	15.7(14.3, 17.0)	8.1(7.1, 9.1)
Swaziland	2007	30.4(28.0, 32.8)	4.8(3.7, 5.9)	2.2(1.4, 2.9)
Tajikistan	2017	20.2(19.0, 21.4)	6.9(6.1, 7.6)	4.1(3.5, 4.7)
Tanzania	2015	38.3(37.0, 39.5)	14.3(13.4, 15.2)	3.8(3.4, 4.3)
Togo	2014	29.5(27.6, 31.4)	16.4(14.8, 18.0)	5.2(4.3, 6.2)
Uganda	2016	31.7(30.0, 33.4)	9.9(8.8, 11.0)	2.3(1.8, 2.9)
Zambia	2013	43.6(42.6, 44.6)	15.3(14.6, 16.1)	5.3(4.8, 5.7)
Zimbabwe	2015	28.0(26.6, 29.5)	7.9(7.0, 8.8)	2.8(2.3, 3.4)

© 2020 Li Z et al. *JAMA Network Open.*

eTable 2. Full Regression Results From the Pooled, Fully Adjusted Model

	Stunting	Underweight	Wasting
	b/se	b/se	b/se
**Child age (reference group: 48-59 months old)**			
12-23 months old	0.928***	0.704***	1.278***
	(0.027)	(0.023)	(0.057)
24-35 months old	1.232***	0.897***	1.009
	(0.023)	(0.018)	(0.027)
36-47 months old	1.160***	0.931***	0.961
	(0.019)	(0.017)	(0.024)
**Child is a female (reference group: male)**	0.885***	0.942***	0.873***
	(0.010)	(0.012)	(0.014)
**Maternal age at birth (year)**	0.995***	0.996***	1.000
	(0.001)	(0.001)	(0.002)
**Birth order (reference group: 1st birth in the household)**			
2nd birth in the household	1.008	0.867**	0.837**
	(0.057)	(0.056)	(0.071)
3rd birth in the household	0.726***	0.681***	0.794**
	(0.047)	(0.050)	(0.077)
4th birth in the household	0.553***	0.553***	0.84
	(0.079)	(0.083)	(0.165)
5th birth in the household	1.671	1.312	1.657
	(0.856)	(0.730)	(1.120)
6th birth in the household	1.012	0.863	3.456
	(0.892)	(0.830)	(4.090)
**Child lives in the rural (reference group: child lives in the urban)**	0.979	0.907***	0.885***
	(0.018)	(0.019)	(0.023)
**Household wealth (reference group: richest)**			
Poorest	1.700***	1.639***	1.171***
	(0.051)	(0.059)	(0.054)
Poorer	1.514***	1.465***	1.078*
	(0.042)	(0.050)	(0.047)
Middle	1.390***	1.329***	1.016
	(0.037)	(0.043)	(0.040)
Richer	1.205***	1.188***	0.992
	(0.030)	(0.035)	(0.038)
**Maternal education (reference group: college and above)**			
No schooling	1.913***	1.837***	1.241***
	(0.064)	(0.070)	(0.054)
Primary education	1.682***	1.558***	1.113**
	(0.056)	(0.059)	(0.049)
Secondary education	1.318***	1.341***	1.175***
	(0.042)	(0.049)	(0.045)
**Maternal height (reference group: 160+cm)**			
<145cm (short maternal height)	4.718***	3.479***	1.153***
	(0.142)	(0.106)	(0.044)

© 2020 Li Z et al. *JAMA Network Open.*

145-149.9 cm	3.009***	2.567***	1.187***
	(0.067)	(0.064)	(0.038)
150-154.9 cm	2.118***	1.816***	1.106***
	(0.041)	(0.040)	(0.033)
155-159.9 cm	1.516***	1.396***	1.080***
	(0.028)	(0.031)	(0.032)
**Maternal BMI (reference group: 25+ kg/m2)**			
<18.5 kg/m2 (low maternal BMI)	1.643***	2.719***	2.281***
	(0.036)	(0.071)	(0.079)
18.5-24.9 kg/m2	1.301***	1.568***	1.529***
	(0.023)	(0.036)	(0.048)
**Child marriage**	1.038***	1.009	0.969*
	(0.013)	(0.014)	(0.017)
**Delivered with skilled birth attendant**	1.155***	1.154***	1.008
	(0.016)	(0.018)	(0.020)
**Family planning need satisfied**	1.031**	1.008	0.957**
	(0.013)	(0.014)	(0.017)
**Dietary diversity score (reference group: richest)**			
Poor	1.222***	1.336***	1.200***
	(0.032)	(0.041)	(0.049)
Relatively poor	1.095***	1.240***	1.190***
	(0.031)	(0.040)	(0.050)
Middle	1.127***	1.190***	1.079*
	(0.031)	(0.038)	(0.045)
Relatively rich	1.091***	1.116***	1.072
	(0.032)	(0.040)	(0.047)
**Delayed breastfeeding**	1.037***	0.983	0.937***
	(0.013)	(0.014)	(0.016)
**Caught by infectious disease in past 2 weeks**	1.039*	1.039*	0.969
	(0.021)	(0.024)	(0.030)
**Not fully vaccinated**	1.041***	1.047***	1.005
	(0.013)	(0.015)	(0.019)
**Oral rehydration therapy for children diarrhea (reference group: no diarrhea)**			
Had diarrhea and used oral rehydration therapy	1.150***	1.236***	1.195***
	(0.030)	(0.038)	(0.046)
Had diarrhea but did not oral rehydration therapy	1.096***	1.246***	1.165***
	(0.030)	(0.038)	(0.049)
**Care seeking for suspected pneumonia (reference group: no suspected pneumonia)**			
With suspected pneumonia and sought for care for it	0.936**	0.949*	0.946
	(0.025)	(0.027)	(0.037)
With suspected pneumonia, but did not seek for care for it	0.958	0.951	0.932
	(0.028)	(0.032)	(0.043)
**Antenatal care (reference group: had eight or more antenatal care visits)**			
Less than four antenatal care visits	1.152***	1.112***	0.959
	(0.029)	(0.031)	(0.033)

© 2020 Li Z et al. *JAMA Network Open.*

Four to seven antenatal care visits	0.998	1.010	0.951
	(0.026)	(0.030)	(0.034)
**Not received vitamin A supplement**	0.996	1.027*	1.040**
	(0.014)	(0.015)	(0.020)
**Unsafe water**	0.974*	0.991	1.071***
	(0.015)	(0.017)	(0.026)
**Unimproved sanitation**	1.098***	1.091***	1.085***
	(0.017)	(0.020)	(0.026)
**Unsafe stool disposal**	1.060***	1.091***	1.008
	(0.016)	(0.019)	(0.024)
**High indoor pollution**	1.055***	1.012	0.996
	(0.021)	(0.024)	(0.027)
**Not used iodized salt**	0.982	0.953**	0.940**
	(0.020)	(0.021)	(0.027)
**Country dummies**	Included	Included	Included
**_cons**	0.113***	0.028***	0.020***
	(0.009)	(0.003)	(0.003)

© 2020 Li Z et al. *JAMA Network Open.*

eTable 3. Magnitudes of 20 Factors Associated With Child Anthropometric Failures From Fully Adjusted Models for the Pooled Sample In three settings: original) controlling only for the 20 factors listed below (n=299,353), sensitivity test 1) controlling for the 20 factors listed below, as well as child birth weight and preceding birth interval (n=128,902), sensitivity test 2) controlling for the 20 factors listed below, as well as women's empowerment (n=142,638)

	Stunting				Underweight				Wasted		
**Factor**	**Original result**	**Sensitivity test 1**	**Sensitivity test 2**		**Original result**	**Sensitivity test 1**	**Sensitivity test 2**		**Original result**	**Sensitivity test 1**	**Sensitivity test 2**
Short maternal stature	4.72(4.45, 5.01)	4.71(4.34, 5.11)	4.96(4.53, 5.44)		3.48(3.28, 3.69)	3.32(3.04, 3.63)	3.75(3.42, 4.12)		1.15(1.07, 1.24)	1.12(1.00, 1.25)	1.24(1.09, 1.41)
Lack of maternal education	1.91(1.79, 2.04)	1.76(1.60, 1.94)	2.03(1.83, 2.25)		1.84(1.71, 1.98)	1.64(1.47, 1.83)	1.96(1.70, 2.25)		1.24(1.14, 1.35)	1.28(1.13, 1.46)	1.26(1.07, 1.48)
Poorest HH wealth	1.70(1.60, 1.80)	1.72(1.58, 1.87)	1.78(1.65, 1.93)		1.64(1.53, 1.76)	1.64(1.47, 1.82)	1.55(1.41, 1.70)		1.17(1.07, 1.28)	1.08(0.95, 1.23)	1.11(0.98, 1.27)
Low maternal BMI	1.64(1.57, 1.71)	1.55(1.46, 1.65)	1.64(1.54, 1.74)		2.72(2.58, 2.86)	2.54(2.36, 2.73)	3.14(2.92, 3.38)		2.28(2.13, 2.44)	2.04(1.85, 2.24)	2.34(2.11, 2.60)
Poor dietary diversity	1.22(1.16, 1.29)	1.16(1.07, 1.25)	1.25(1.18, 1.34)		1.34(1.26, 1.42)	1.32(1.20, 1.45)	1.30(1.20, 1.41)		1.20(1.11, 1.30)	1.29(1.14, 1.46)	1.20(1.07, 1.35)
No SBA	1.15(1.12, 1.19)	1.05(1.00, 1.11)	1.18(1.13, 1.22)		1.15(1.12, 1.19)	1.08(1.01, 1.14)	1.21(1.15, 1.27)		1.01(0.97, 1.05)	1.07(0.99, 1.16)	1.07(1.00, 1.14)
Less than four ANC visits	1.15(1.10, 1.21)	1.12(1.05, 1.20)	1.07(0.99, 1.15)		1.11(1.05, 1.17)	1.05(0.97, 1.12)	1.03(0.93, 1.13)		0.96(0.90, 1.03)	0.87(0.80, 0.94)	1.01(0.88, 1.14)
Unimproved sanitation	1.10(1.06, 1.13)	1.05(1.01, 1.10)	1.11(1.06, 1.15)		1.09(1.05, 1.13)	1.09(1.04, 1.15)	1.09(1.04, 1.15)		1.08(1.04, 1.14)	1.13(1.06, 1.21)	1.09(1.02, 1.18)
Had diarrhea, but not used ORT	1.10(1.04, 1.16)	1.09(1.00, 1.19)	1.07(1.00, 1.14)		1.25(1.17, 1.32)	1.11(1.00, 1.23)	1.26(1.17, 1.36)		1.17(1.07, 1.26)	1.06(0.93, 1.22)	1.34(1.20, 1.50)
Unsafe stool disposal	1.06(1.03, 1.09)	1.09(1.04, 1.14)	1.02(0.98, 1.06)		1.09(1.05, 1.13)	1.11(1.06, 1.17)	1.09(1.04, 1.14)		1.01(0.96, 1.06)	1.00(0.94, 1.07)	1.04(0.97, 1.11)
High indoor pollution	1.05(1.01, 1.10)	1.02(0.96, 1.08)	1.13(1.06, 1.21)		1.01(0.97, 1.06)	0.99(0.93, 1.05)	1.06(0.97, 1.15)		1.00(0.94, 1.05)	1.04(0.96, 1.12)	0.97(0.88, 1.07)
Not fully vaccinated	1.04(1.02, 1.07)	1.03(0.99, 1.07)	1.05(1.01, 1.08)		1.05(1.02, 1.08)	1.02(0.97, 1.06)	1.08(1.04, 1.13)		1.01(0.97, 1.04)	0.99(0.94, 1.05)	1.03(0.96, 1.09)
Infectious disease	1.04(1.00, 1.08)	0.98(0.92, 1.05)	1.05(1.00, 1.11)		1.04(0.99, 1.09)	1.06(0.99, 1.14)	1.07(1.00, 1.14)		0.97(0.91, 1.03)	0.97(0.88, 1.06)	0.94(0.86, 1.03)
Child marriage	1.04(1.01, 1.06)	1.02(0.99, 1.06)	1.01(0.98, 1.05)		1.01(0.98, 1.04)	1.02(0.97, 1.06)	0.98(0.95, 1.02)		0.97(0.94, 1.00)	0.99(0.94, 1.05)	0.99(0.93, 1.04)
Delayed breastfeeding	1.04(1.01, 1.06)	1.02(0.99, 1.06)	1.01(0.98, 1.05)		0.98(0.96, 1.01)	0.97(0.93, 1.01)	0.97(0.93, 1.01)		0.94(0.90, 0.97)	0.94(0.89, 0.99)	0.96(0.91, 1.02)
FP need unsatisfied	1.03(1.00, 1.06)	1.03(0.99, 1.07)	1.04(1.01, 1.08)		1.01(0.98, 1.04)	1.03(0.98, 1.07)	1.04(1.00, 1.08)		0.96(0.92, 0.99)	0.97(0.92, 1.02)	0.96(0.91, 1.02)
No vit A supplement	1.00(0.97, 1.02)	1.04(1.00, 1.09)	1.03(0.99, 1.07)		1.03(1.00, 1.06)	1.09(1.03, 1.14)	1.00(0.96, 1.05)		1.04(1.00, 1.08)	1.04(0.98, 1.11)	1.03(0.97, 1.10)
No iodized salt	0.98(0.94, 1.02)	1.01(0.95, 1.07)	0.98(0.94, 1.03)		0.95(0.91, 1.00)	0.98(0.92, 1.05)	0.98(0.93, 1.04)		0.94(0.89, 0.99)	0.93(0.85, 1.01)	0.90(0.82, 0.97)
Unsafe water	0.97(0.95, 1.00)	0.97(0.92, 1.02)	0.97(0.94, 1.01)		0.99(0.96, 1.03)	0.99(0.92, 1.05)	0.97(0.93, 1.02)		1.07(1.02, 1.12)	1.01(0.94, 1.08)	1.04(0.98, 1.11)
No care seeking for susp pneu	0.96(0.90, 1.01)	1.06(0.96, 1.16)	1.07(0.98, 1.16)		0.95(0.89, 1.02)	1.01(0.90, 1.13)	1.11(1.00, 1.23)		0.93(0.85, 1.02)	1.02(0.88, 1.18)	1.05(0.90, 1.22)

Note:

1. Abbreviations - HH: household, BMI: body mass index; SBA: skilled birth attendant; ANC: antenatal care; ORT: oral rehydration therapy; FP: family planning; vit: vitamin; susp pneu: suspected pneumonia.

© 2020 Li Z et al. *JAMA Network Open.*

eFigure 1. Flow Diagram Showing Exclusions and Final Sample Sizes of the Study Population, Using the Most Recent Pooled Demographic Health Survey Data Since 2010

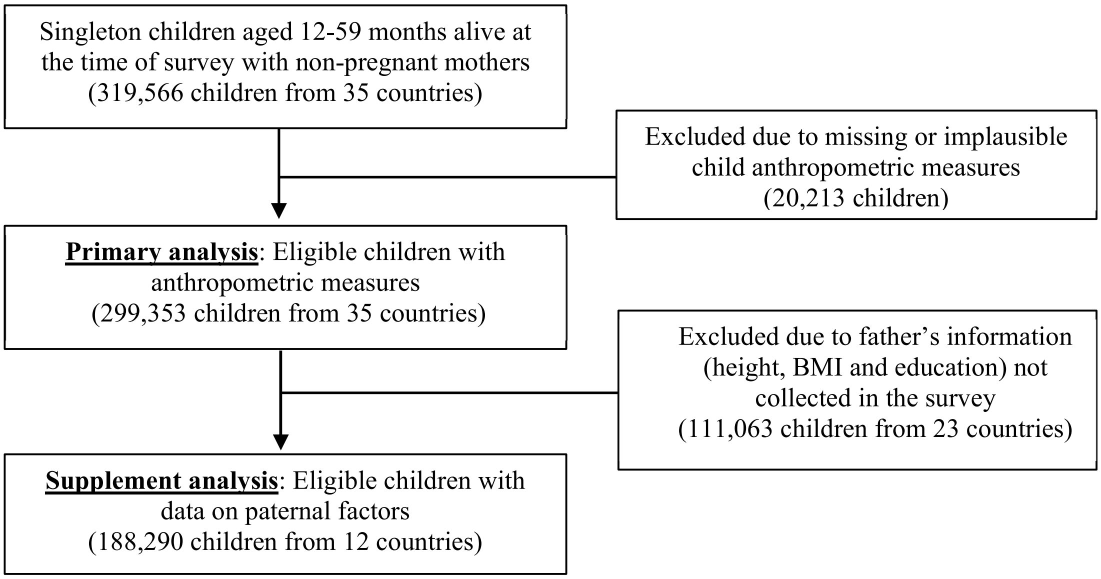

© 2020 Li Z et al. *JAMA Network Open.*

eFigure 2. Relative Ranking of 20 Factors Associated With Child Anthropometric Failures From Single Adjusted Models for the Pooled Sample (N = 299,353)
A) stunting, B) underweight, and C) wasting, odds ratio (OR) and 95% confidence interval (CI)

A) Stunting

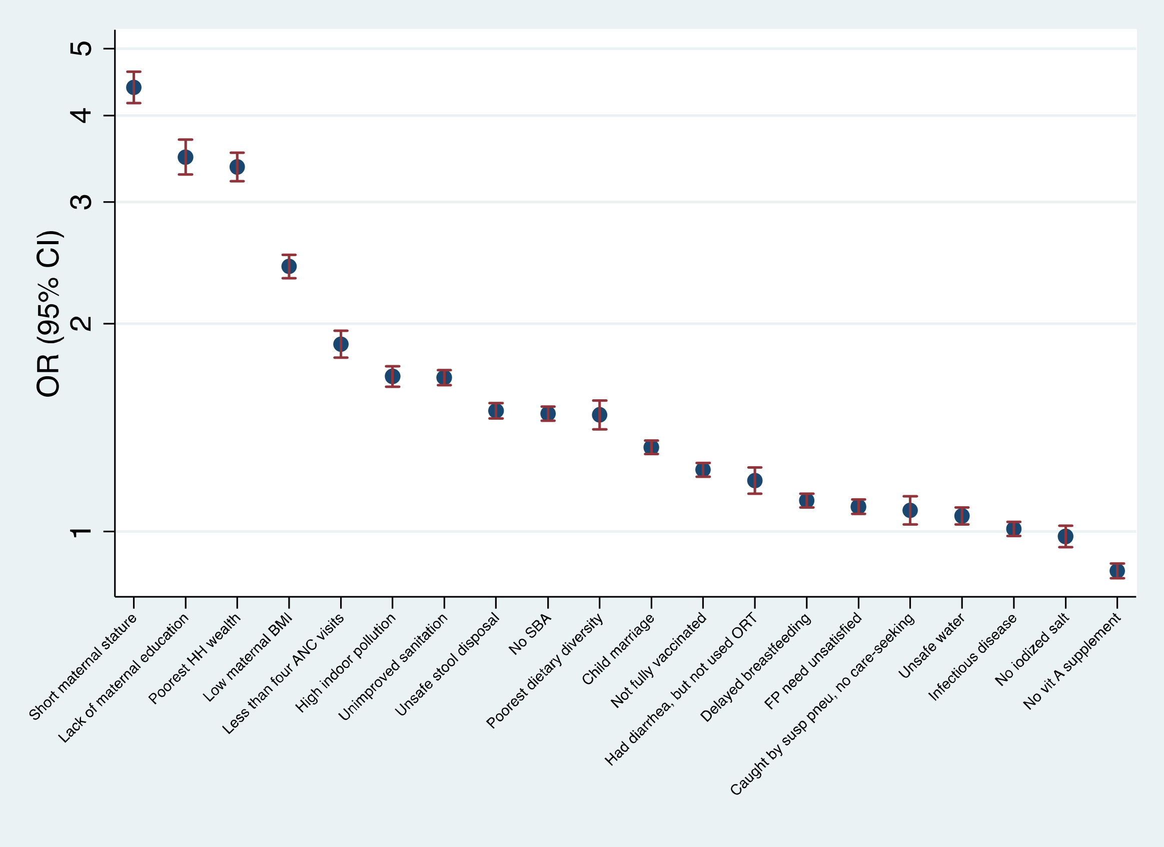

Note:

1. We use logarithmic scale for the y axis.
2. Short maternal statue: maternal height <145cm; low maternal BMI: maternal BMI <18.5 kg/m^2; child marriage: mother's age at marriage <18 years old; delayed breastfeeding: child was not initially breastfed within one hour after born; infectious disease: child was caught by infectious diseases two weeks prior to the survey,
3. Abbreviations - HH: household, BMI: body mass index; SBA: skilled birth attendant; ANC: antenatal care; ORT: oral rehydration therapy; FP: family planning; vit: vitamin; susp pneu: suspected pneumonia.

© 2020 Li Z et al. *JAMA Network Open.*

B) Underweight

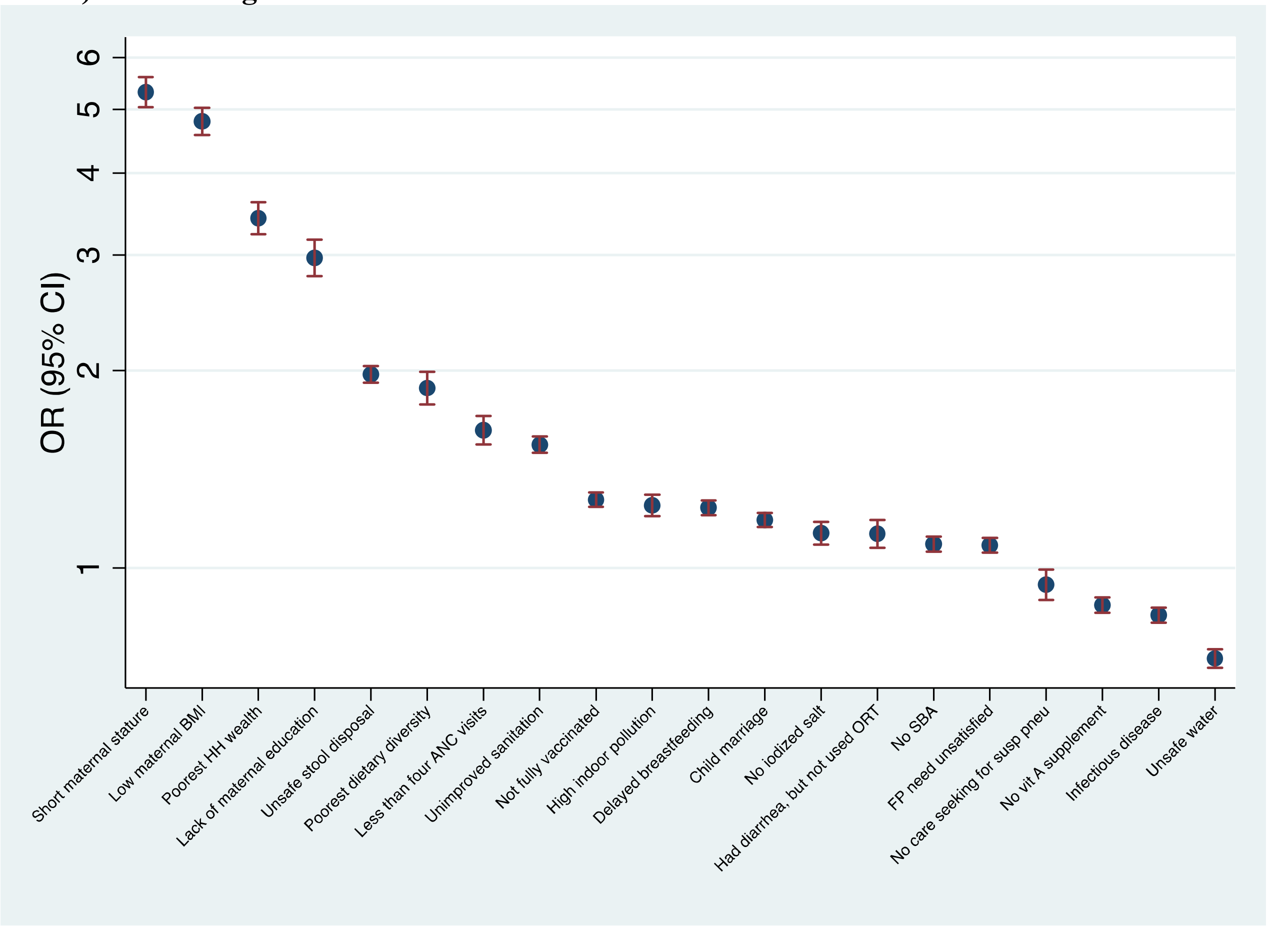

© 2020 Li Z et al. *JAMA Network Open.*

C) Wasting

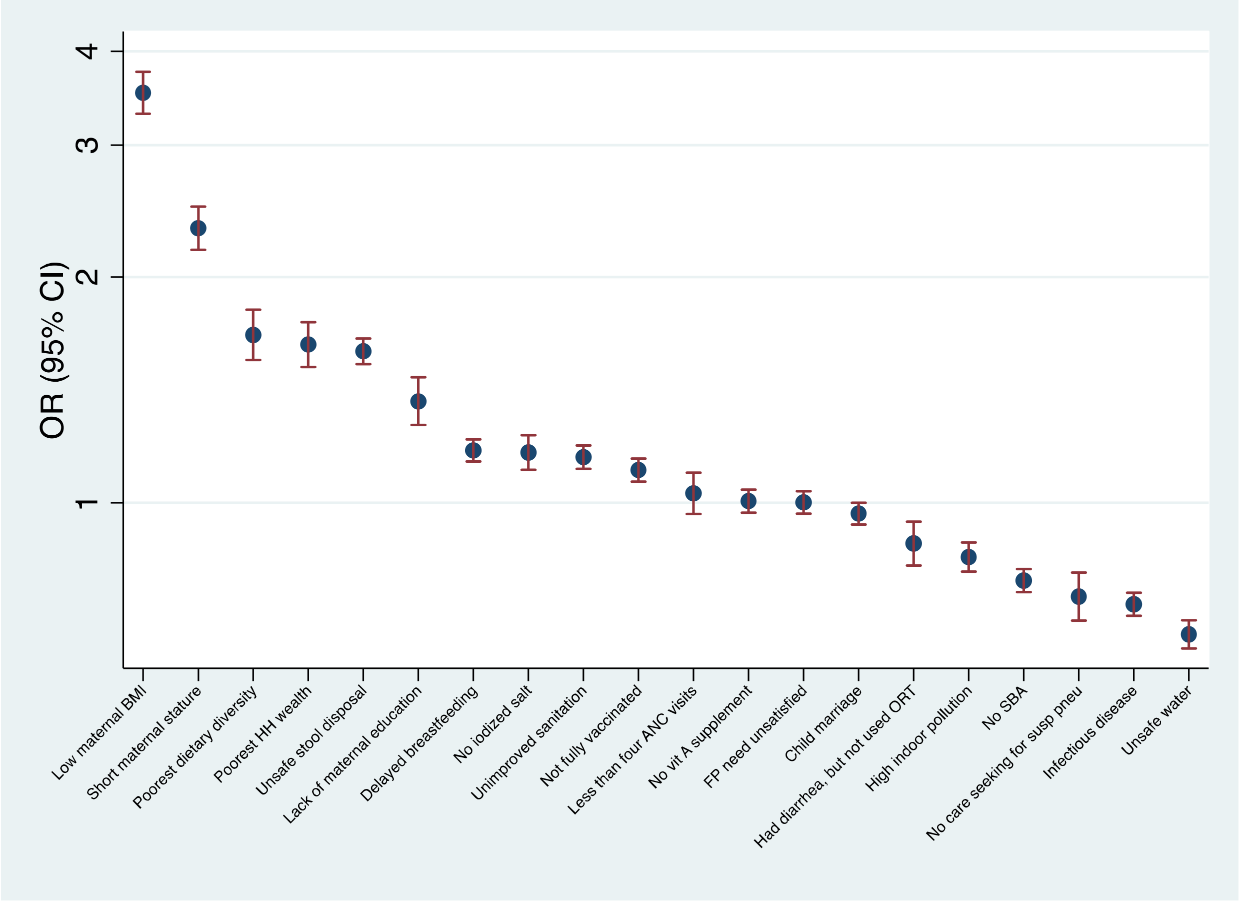

© 2020 Li Z et al. *JAMA Network Open.*

eFigure 3. Country-Specific Ranking of 20 Factors Associated With Child Anthropometric Failures From Fully Adjusted Models on Underweight

	BJ	BF	BI	CM	CI	CD	ET	GM	GH	GN	HT	IN	KE	KG	KM	LS	LR	MW	ML	MZ	MM	NA	NP	NE	PE	ST	SL	SZ	TD	TJ	TZ	TG	UG	ZM	ZW
Short maternal stature	1	1	2	4	3	1	1	1	3	9	3	1	1	11	3	3	1	1	9	4	1	20	2	2	3	3	2	2	11	4	1	2	1	1	6
Lack of maternal education	3	3	19	2	1	6	2	16	1	5	2	3	4	15	2	1	12	16	1	1	18	11	7	5	4	19	20	6	4	15	7	6	2	6	2
Poorest HH wealth	5	6	3	5	13	3	7	12	13	10	7	4	3	16	5	2	7	3	4	5	15	5	11	9	1	1	13	1	7	13	4	5	4	3	17
Low maternal BMI	2	2	1	1	2	2	3	3	2	1	4	2	2	1	1	5	2	2	2	2	2	6	1	1	2	2	1	3	1	1	2	3	3	2	1
Poor dietary diversity	11	13	15	3	9	5	5	13	9	6	19	5	19	3	12	20	3	4	3	6	9	14	6	6	20	16	9	18	8	3	11	12	6	20	20
No SBA	4	5	5	8	17	8	12	5	4	3	15	10	6	13	11	7	9	8	8	20	4	8	13	15	11	9	15	8	6	7	19	9	8	9	11
Less than four ANC visits	10	20	17	6	20	20	4	2	14	4	9	8	7	18	16	10	14	20	14	3	3	16	8	17	19	13	5	20	5	5	20	1	5	4	10
Unimproved sanitation	17	9	12	20	7	15	8	9	10	19	6	7	15	2	17	14	6	19	10	15	11	17	3	14	7	15	3	17	12	6	16	19	19	10	8
Had diarrhea, but not used ORT	9	12	6	7	14	12	6	4	19	7	11	6	8	12	7	4	4	7	6	7	20	3	20	11	8	5	6	4	2	2	12	8	14	16	18
Unsafe stool disposal	18	8	10	14	8	9	10	11	6	14	13	9	9	5	8	8	11	5	7	12	6	4	14	3	13	10	19	10	10	8	8	13	7	18	15
High indoor pollution	7	4	20	11	4	16	20	10	8	2	1	13	5	6	18	17	20	15	20	8	10	2	4	8	12	20	16	19	9	16	3	4	13	5	5
Not fully vaccinated	12	11	7	9	11	17	13	17	11	13	10	14	11	19	4	16	13	6	11	13	13	9	15	13	15	6	8	7	3	19	9	7	15	8	14
Infectious disease	6	7	18	16	6	10	17	20	5	15	20	12	14	4	13	19	10	11	12	9	5	19	5	4	9	17	18	14	14	20	10	14	20	7	9
Child marriage	16	16	13	19	16	13	16	15	20	17	17	15	18	14	15	9	16	12	15	17	7	18	18	10	16	7	17	12	17	14	18	10	17	12	7
Delayed breastfeeding	15	18	11	17	19	18	15	18	18	20	12	16	17	9	10	6	8	9	13	18	14	7	17	12	10	11	11	11	19	10	15	17	10	11	13
FP need unsatisfied	8	10	16	13	12	19	11	14	16	12	14	17	12	8	6	13	5	14	17	14	16	12	19	16	17	18	10	5	15	12	5	11	16	13	4
No vit A supplement	13	17	9	12	10	11	19	6	12	8	16	11	16	7	14	18	15	17	16	16	12	10	9	7	6	4	14	16	18	18	17	15	9	19	19
No iodized salt	20	14	4	10	5	4	9	19	7	11	8	19	20	17	19	11	19	13	19	19	17	15	12	18	5	12	12	13	16	9	6	18	12	17	3
Unsafe water	19	15	14	15	18	14	18	8	15	18	18	18	10	20	9	12	18	10	18	11	8	13	10	19	18	8	4	9	20	17	14	16	11	15	12
No care seeking for susp pneu	14	19	8	18	15	7	14	7	17	16	5	20	13	10	20	15	17	18	5	10	19	1	16	20	14	14	7	15	13	11	13	20	18	14	16

Label:

Rank 1st   Rank 2nd   Rank 3rd-4th   Rank 5th-7th   Rank 8th-12nd   Rank 13th-20th

Note:

1. BJ= Benin, BF= Burkina Faso, BI= Burundi, CM=Cameroon, CI=Côte d'Ivoire, CD=The Democratic Republic of the Congo, ET=Ethiopia, GM=Gambia, GH=Ghana, GN=Guinea, HT=Haiti, IN=India, KE=Kenya, KG=Kyrgyzstan, KM=Comoros, LS=Lesotho, LR=Liberia, MW=Malawi, ML=Mali, MZ=Mozambique, MM=Myanmar, NA=Namibia, NP=Nepal, NE=Niger, PE=Peru, ST= São Tomé and Príncipe, SL=Sierra Leone, SZ=Swaziland, TD=Chad, TJ=Tajikistan, TZ=Tanzania, TG=Togo, UG=Uganda, ZM=Zambia, ZW=Zimbabwe.
2. Short maternal statue: maternal height <145cm; low maternal BMI: maternal BMI <18.5 kg/m^2; child marriage: mother's age at marriage <18 years old; delayed breastfeeding: child was not initially breastfed within one hour after born; infectious disease: child was caught by infectious diseases two weeks prior to the survey.
3. Abbreviations - HH: household, BMI: body mass index; SBA: skilled birth attendant; ANC: antenatal care; ORT: oral rehydration therapy; FP: family planning; vit: vitamin; susp pneu: suspected pneumonia.

© 2020 Li Z et al. *JAMA Network Open.*

eFigure 4. Country-Specific Odds Ratios for 20 Factors Associated With Child Anthropometric Failures From Fully Adjusted Models on Underweight

	BJ	BF	BI	CM	CI	CD	ET	GM	GH	GN	HT	IN	KE	KG	KM	LS	LR	MW	ML	MZ	MM	NA	NP	NE	PE	ST	SL	SZ	TD	TJ	TZ	TG	UG	ZM	ZW
Short maternal stature	4.2	6.1	3.8	2.6	2.4	3.4	4.0	6.2	2.3	1.6	6.9	3.4	4.9	1.0	1.8	5.2	6.7	6.8	1.2	4.0	4.2	0.2	2.6	1.6	6.2	3.2	2.1	5.0	1.1	1.7	5.8	5.6	7.0	4.3	1.3
Lack of maternal education	2.3	1.7	0.7	5.2	18.7	1.4	2.8	0.9	11.4	1.7	7.5	1.8	2.0	1.0	3.0	33.3	1.1	0.9	3.7	5.0	0.7	1.2	1.2	1.5	2.7	0.7	0.7	1.9	1.4	0.9	1.2	1.7	5.6	1.4	2.1
Poorest HH wealth	1.3	1.3	2.8	2.5	1.1	2.6	1.8	1.1	1.0	1.4	1.5	1.6	2.6	0.9	1.5	5.7	1.3	2.1	1.6	2.2	0.9	3.1	1.2	1.2	13.9	6.0	1.1	5.4	1.3	1.0	2.0	1.8	2.7	1.9	0.8
Low maternal BMI	3.5	3.7	6.8	5.9	7.3	2.6	2.7	2.0	4.1	2.7	4.3	2.6	3.2	5.5	3.2	2.8	5.6	3.6	2.6	4.8	3.5	2.0	3.6	3.2	7.0	5.7	2.1	3.7	2.8	3.2	3.2	5.0	3.3	2.7	6.4
Poor dietary diversity	1.1	1.0	1.0	3.1	1.1	1.4	1.9	1.1	1.2	1.7	0.8	1.4	0.8	1.9	1.1	0.4	1.6	1.5	1.6	1.7	1.2	0.8	1.3	1.4	0.5	0.8	1.3	0.6	1.2	1.8	1.1	1.2	1.4	0.9	0.5
No SBA	1.6	1.4	1.6	1.4	0.7	1.2	1.0	1.3	1.5	1.8	1.1	1.1	1.3	1.0	1.2	1.2	1.2	1.3	1.3	0.8	1.5	1.4	1.0	1.0	1.1	1.3	1.0	1.2	1.3	1.2	0.9	1.2	1.2	1.2	1.2
Less than four ANC visits	1.1	0.1	0.8	1.9	0.5	0.5	2.2	2.3	0.9	1.7	1.4	1.1	1.3	0.8	0.9	0.9	0.9	0.6	1.0	4.0	1.6	0.7	1.2	1.0	0.8	1.1	1.4	0.2	1.4	1.5	0.8	7.3	1.6	1.9	1.2
Unimproved sanitation	1.0	1.2	1.1	1.0	1.2	1.1	1.3	1.2	1.1	0.9	1.5	1.1	1.0	2.5	0.8	0.7	1.4	0.7	1.1	1.0	1.0	0.6	1.8	1.0	1.3	0.9	1.5	0.7	1.1	1.2	0.9	0.9	0.7	1.2	1.3
Had diarrhea, but not used ORT	1.2	1.1	1.5	1.5	1.0	1.1	1.8	1.5	0.6	1.6	1.2	1.2	1.2	1.0	1.4	3.0	1.5	1.3	1.3	1.6	0.4	3.5	0.8	1.1	1.2	2.5	1.4	3.0	1.4	2.0	1.1	1.3	1.0	0.9	0.7
Unsafe stool disposal	0.9	1.2	1.2	1.2	1.2	1.2	1.1	1.1	1.3	1.1	1.2	1.1	1.1	1.5	1.2	1.1	1.1	1.3	1.3	1.1	1.4	3.4	1.0	1.6	1.0	1.2	0.9	1.1	1.2	1.2	1.2	1.2	1.4	0.9	0.9
High indoor pollution	1.2	1.5	0.3	1.2	1.9	1.0	0.7	1.1	1.3	2.0	7.8	1.0	1.8	1.4	0.8	0.6	0.2	1.0	0.8	1.3	1.1	4.0	1.5	1.2	1.0	0.4	1.0	0.4	1.2	0.9	2.1	3.7	1.0	1.5	1.4
Not fully vaccinated	1.1	1.1	1.4	1.3	1.1	1.0	1.0	0.9	1.1	1.1	1.2	1.0	1.0	0.8	1.6	0.6	1.0	1.3	1.1	1.0	1.0	1.3	0.9	1.0	1.0	1.7	1.3	1.4	1.4	0.8	1.2	1.4	0.9	1.2	0.9
Infectious disease	1.3	1.2	0.8	1.1	1.6	1.2	0.9	0.6	1.5	1.0	0.7	1.0	1.0	1.9	1.0	0.5	1.2	1.1	1.1	1.3	1.5	0.2	1.5	1.5	1.1	0.7	0.9	0.8	1.0	0.7	1.2	1.0	0.7	1.3	1.2
Child marriage	1.0	1.0	1.1	1.0	0.9	1.1	1.0	1.0	0.6	1.0	0.8	1.0	0.8	1.0	0.9	1.0	0.9	1.1	1.0	0.9	1.2	0.5	0.9	1.1	1.0	1.6	1.0	0.9	0.9	0.9	0.9	1.2	0.9	1.1	1.3
Delayed breastfeeding	1.0	0.9	1.1	1.0	0.6	0.9	1.0	0.8	0.7	0.9	1.2	1.0	0.9	1.0	1.2	1.3	1.3	1.2	1.0	0.9	0.9	1.6	0.9	1.0	1.1	1.2	1.2	1.0	0.8	1.0	1.0	0.9	1.1	1.1	1.0
FP need unsatisfied	1.2	1.2	1.0	1.2	1.1	0.8	1.0	1.0	0.8	1.1	1.1	1.0	1.0	1.1	1.5	0.7	1.4	1.0	1.0	1.0	0.8	1.0	0.9	1.0	0.9	0.7	1.2	2.0	1.0	1.0	1.3	1.2	0.9	1.0	1.4
No vit A supplement	1.1	0.9	1.2	1.2	1.1	1.1	0.8	1.2	1.0	1.6	1.1	1.0	0.9	1.3	1.0	0.6	0.9	0.8	1.0	1.0	1.0	1.2	1.2	1.3	1.5	2.6	1.0	0.7	0.8	0.8	0.9	1.0	1.1	0.9	0.7
No iodized salt	0.9	1.0	2.0	1.2	1.6	1.8	1.2	0.7	1.3	1.3	1.4	0.9	0.7	0.8	0.7	0.8	0.3	1.1	1.0	0.9	0.8	0.7	1.1	0.9	1.5	1.2	1.2	0.9	0.9	1.1	1.3	0.9	1.0	0.9	1.6
Unsafe water	0.9	1.0	1.1	1.1	0.7	1.1	0.9	1.2	0.9	1.0	0.8	1.0	1.1	0.6	1.2	0.8	0.7	1.2	1.0	1.1	1.2	1.0	1.2	0.9	0.9	1.3	1.5	1.1	0.8	0.8	1.0	1.0	1.1	1.0	1.0
No care seeking for susp pneu	1.0	0.9	1.3	1.0	1.0	1.2	1.0	1.2	0.7	1.0	1.6	0.9	1.0	1.0	0.7	0.7	0.8	0.8	1.4	1.2	0.7	5.1	0.9	0.6	1.0	1.0	1.3	0.8	1.0	1.0	1.0	0.7	0.8	1.0	0.9

Label: odds ratios

- >=4.0
- [2.0, 4.0)
- [1.3, 2.0)
- [1.1, 1.3)
- [1.0, 1.1)
- <1.0

Note:

1. BJ= Benin, BF= Burkina Faso, BI= Burundi, CM=Cameroon, CI=Côte d'Ivoire, CD=The Democratic Republic of the Congo, ET=Ethiopia, GM=Gambia, GH=Ghana, GN=Guinea, HT=Haiti, IN=India, KE=Kenya, KG=Kyrgyzstan, KM=Comoros, LS=Lesotho, LR=Liberia, MW=Malawi, ML=Mali, MZ=Mozambique, MM=Myanmar, NA=Namibia, NP=Nepal, NE=Niger, PE=Peru, ST= São Tomé and Príncipe, SL=Sierra Leone, SZ=Swaziland, TD=Chad, TJ=Tajikistan, TZ=Tanzania, TG=Togo, UG=Uganda, ZM=Zambia, ZW=Zimbabwe.
2. Short maternal statue: maternal height <145cm; low maternal BMI: maternal BMI <18.5 kg/m^2; child marriage: mother's age at marriage <18 years old; delayed breastfeeding: child was not initially breastfed within one hour after born; infectious disease: child was caught by infectious diseases two weeks prior to the survey.
3. Abbreviations - HH: household, BMI: body mass index; SBA: skilled birth attendant; ANC: antenatal care; ORT: oral rehydration therapy; FP: family planning; vit: vitamin; susp pneu: suspected pneumonia.

© 2020 Li Z et al. *JAMA Network Open.*

eFigure 5. Country-Specific Ranking of 20 Factors Associated With Child Anthropometric Failures From Fully Adjusted Models on Wasting

	BJ	BF	BI	CM	CI	CD	ET	GM	GH	GN	HT	IN	KE	KG	KM	LS	LR	MW	ML	MZ	MM	NA	NP	NE	PE	ST	SL	SZ	TD	TJ	TZ	TG	UG	ZM	ZW
Short maternal stature	3	5	19	9	9	2	6	9	11	13	18	5	11	10	2	8	1	1	20	1	17	11	20	1	3	20	20	10	1	9	3	3	3	5	2
Lack of maternal education	17	4	20	5	1	1	7	6	1	5	1	3	1	8	3	10	14	20	2	4	19	2	6	19	14	7	5	19	6	19	8	4	19	20	12
Poorest HH wealth	20	10	3	1	13	4	3	20	14	19	6	4	2	7	4	13	8	18	7	2	3	1	19	4	16	16	19	2	16	20	18	2	2	2	6
Low maternal BMI	1	2	1	4	2	5	1	2	2	3	2	1	5	2	10	4	3	3	1	3	1	7	2	2	1	11	1	1	2	1	1	1	1	6	1
Poor dietary diversity	14	1	17	2	14	8	5	4	20	2	19	2	19	15	1	2	4	5	17	6	2	20	1	11	19	19	2	6	11	8	5	18	13	13	3
No SBA	10	11	5	3	18	3	9	10	6	4	12	15	7	11	18	7	10	7	12	19	6	10	14	18	10	14	13	18	13	13	10	7	7	3	16
Less than four ANC visits	19	18	15	20	20	20	2	1	3	1	4	18	3	19	19	16	13	13	10	20	5	6	13	20	17	1	10	20	4	3	19	20	18	1	9
Unimproved sanitation	13	16	10	10	10	19	19	14	8	10	9	7	18	6	8	1	9	14	4	14	14	14	4	10	7	4	4	5	3	11	16	12	8	7	7
Had diarrhea, but not used ORT	5	6	4	6	16	10	8	12	5	6	13	9	4	12	5	9	5	10	11	12	18	8	9	6	9	10	3	11	8	2	20	13	17	8	17
Unsafe stool disposal	18	3	14	16	7	18	10	8	16	18	8	11	6	16	6	17	12	17	9	9	4	5	10	5	15	8	7	8	7	6	7	17	10	18	15
High indoor pollution	2	20	18	19	3	9	4	16	19	7	16	10	13	14	16	15	11	12	3	15	13	15	5	3	2	17	14	14	5	14	2	19	20	19	5
Not fully vaccinated	6	17	8	8	17	12	16	17	17	16	10	13	9	4	9	6	15	8	13	7	12	12	16	8	5	9	15	13	9	12	6	8	16	14	18
Infectious disease	9	12	11	13	4	6	18	19	18	12	17	14	17	20	17	20	18	4	5	8	10	19	7	16	6	18	12	7	15	18	4	11	11	16	8
Child marriage	12	13	7	17	5	14	11	13	9	15	20	17	16	5	14	14	19	9	15	17	11	9	17	14	12	6	8	17	12	7	17	5	12	9	10
Delayed breastfeeding	15	14	12	7	19	11	15	11	15	20	7	19	14	18	15	11	7	15	8	11	7	4	12	15	11	5	16	4	10	5	9	9	9	17	14
FP need unsatisfied	16	9	16	11	12	16	17	15	13	17	11	16	15	9	7	18	16	11	16	18	16	17	15	9	8	13	9	3	18	15	12	15	15	10	20
No vit A supplement	8	7	13	15	15	7	20	5	7	8	5	8	8	3	12	3	6	16	18	5	20	18	18	7	20	3	17	9	17	17	14	6	14	11	19
No iodized salt	7	19	2	14	11	17	12	18	12	9	3	12	20	13	20	19	20	2	14	10	15	16	3	17	18	2	18	12	20	4	13	14	4	4	4
Unsafe water	11	15	6	18	6	15	13	7	10	14	14	6	10	17	13	12	17	6	19	16	8	13	11	13	4	15	11	15	19	16	11	10	5	12	11
No care seeking for susp pneu	4	8	9	12	8	13	14	3	4	11	15	20	12	1	11	5	2	19	6	13	9	3	8	12	13	12	6	16	14	10	15	16	6	15	13

Label:

Rank 1st; Rank 2nd; Rank 3rd-4th; Rank 5th-7th; Rank 8th-12nd; Rank 13th-20th

Note:

1. BJ= Benin, BF= Burkina Faso, BI= Burundi, CM=Cameroon, CI=Côte d'Ivoire, CD=The Democratic Republic of the Congo, ET=Ethiopia, GM=Gambia, GH=Ghana, GN=Guinea, HT=Haiti, IN=India, KE=Kenya, KG=Kyrgyzstan, KM=Comoros, LS=Lesotho, LR=Liberia, MW=Malawi, ML=Mali, MZ=Mozambique, MM=Myanmar, NA=Namibia, NP=Nepal, NE=Niger, PE=Peru, ST= São Tomé and Príncipe, SL=Sierra Leone, SZ=Swaziland, TD=Chad, TJ=Tajikistan, TZ=Tanzania, TG=Togo, UG=Uganda, ZM=Zambia, ZW=Zimbabwe.
2. Short maternal statue: maternal height <145cm; low maternal BMI: maternal BMI <18.5 kg/m^2; child marriage: mother's age at marriage <18 years old; delayed breastfeeding: child was not initially breastfed within one hour after born; infectious disease: child was caught by infectious diseases two weeks prior to the survey.
3. Abbreviations - HH: household, BMI: body mass index; SBA: skilled birth attendant; ANC: antenatal care; ORT: oral rehydration therapy; FP: family planning; vit: vitamin; susp pneu: suspected pneumonia.

© 2020 Li Z et al. *JAMA Network Open.*

eFigure 6. Country-Specific Odds Ratios for 20 Factors Associated With Child Anthropometric Failures From Fully Adjusted Models on Wasting

	BJ	BF	BI	CM	CI	CD	ET	GM	GH	GN	HT	IN	KE	KG	KM	LS	LR	MW	ML	MZ	MM	NA	NP	NE	PE	ST	SL	SZ	TD	TJ	TZ	TG	UG	ZM	ZW
Short maternal stature	2.0	1.5	0.5	1.4	1.0	1.8	1.7	1.0	1.0	0.9	0.7	1.2	1.0	1.0	1.6	1.1	5.1	8.7	0.6	2.7	0.6	1.0	0.4	3.1	2.1	0.4	0.2	1.0	5.3	1.0	1.9	2.2	2.4	1.5	3.3
Lack of maternal education	0.8	1.8	0.1	1.7	3.3	4.1	1.6	1.3	44.0	1.7	7.4	1.2	4.0	1.1	1.6	1.0	1.0	0.2	2.5	1.9	0.6	5.1	1.8	0.7	1.0	1.3	1.6	0.1	1.4	0.6	1.2	1.9	0.5	0.6	1.0
Poorest HH wealth	0.5	1.1	1.9	5.1	0.8	1.7	1.9	0.6	0.7	0.6	1.4	1.2	3.1	1.2	1.5	0.8	1.1	0.7	1.2	2.6	1.4	16.5	0.5	1.7	0.8	0.8	0.7	16.6	0.9	0.6	0.7	4.1	4.3	1.7	1.4
Low maternal BMI	5.7	2.8	16.4	2.2	2.9	1.4	3.7	3.2	3.1	2.0	2.8	2.3	1.5	2.7	1.3	2.0	2.7	3.0	2.6	2.0	2.1	1.6	2.2	2.8	11.6	1.1	2.2	40.0	4.6	3.4	2.5	4.5	5.8	1.2	3.8
Poor dietary diversity	1.0	4.5	0.6	2.4	0.8	1.1	1.7	1.7	0.2	4.6	0.6	1.2	0.7	0.8	2.2	5.9	2.0	1.9	0.8	1.7	1.4	0.2	2.4	1.0	0.6	0.7	1.9	1.7	1.1	1.0	1.7	0.6	0.8	1.1	2.6
No SBA	1.2	1.0	1.6	2.3	0.6	1.8	1.3	1.0	1.7	1.7	1.0	1.0	1.2	1.0	0.7	1.6	1.0	1.3	1.1	0.6	1.3	1.2	1.0	0.8	1.2	1.0	1.1	0.2	1.0	0.9	1.1	1.2	1.0	1.6	0.6
Less than four ANC visits	0.6	0.6	1.0	0.5	0.3	0.7	2.6	14.0	2.9	6.9	1.4	0.9	2.2	0.3	0.7	0.3	1.0	0.8	1.1	0.5	1.3	2.6	1.0	0.6	0.8	4.7	1.2	0.1	1.5	2.2	0.5	0.3	0.5	2.5	1.1
Unimproved sanitation	1.1	0.8	1.1	1.3	1.0	0.8	0.7	0.9	1.6	1.1	1.3	1.1	0.8	1.3	1.4	16.8	1.1	0.8	1.3	0.9	0.9	0.6	2.0	1.0	1.3	1.6	1.6	1.7	1.6	1.0	0.8	1.0	0.9	1.2	1.2
Had diarrhea, but not used ORT	1.6	1.4	1.7	1.5	0.7	1.0	1.6	1.0	2.4	1.5	0.8	1.0	1.8	1.0	1.5	1.0	1.8	1.1	1.1	0.9	0.6	1.5	1.3	1.4	1.2	1.2	1.9	1.0	1.3	2.7	0.5	0.9	0.5	1.2	0.6
Unsafe stool disposal	0.7	1.9	1.0	0.8	1.0	0.8	1.2	1.3	0.7	0.7	1.3	1.0	1.4	0.7	1.4	0.2	1.0	0.7	1.1	1.3	1.3	3.7	1.3	1.4	0.9	1.3	1.3	1.4	1.3	1.3	1.3	0.7	0.9	0.8	0.7
High indoor pollution	2.4	0.6	0.5	0.6	2.3	1.0	1.9	0.9	0.3	1.3	0.7	1.0	1.0	0.8	0.8	0.5	1.0	0.9	1.9	0.8	0.9	0.5	1.8	1.8	2.5	0.8	1.0	0.7	1.4	0.9	2.0	0.6	0.4	0.8	1.9
Not fully vaccinated	1.3	0.8	1.2	1.4	0.7	0.9	0.9	0.8	0.5	0.8	1.3	1.0	1.1	1.6	1.3	1.9	0.9	1.3	1.0	1.4	0.9	0.8	0.9	1.1	1.7	1.2	1.0	0.8	1.2	1.0	1.6	1.2	0.6	1.0	0.6
Infectious disease	1.2	1.0	1.1	1.0	2.0	1.4	0.8	0.7	0.3	0.9	0.7	1.0	0.8	0.6	0.7	0.1	0.6	2.0	1.3	1.3	1.0	0.3	1.7	0.8	1.6	0.8	1.1	1.6	0.9	0.7	1.9	1.0	0.9	1.0	1.1
Child marriage	1.1	1.0	1.4	0.8	1.3	0.9	1.1	0.9	1.1	0.8	0.6	1.0	0.8	1.3	0.8	0.5	0.6	1.3	0.9	0.7	1.0	1.3	0.8	0.9	1.1	1.4	1.3	0.3	1.1	1.2	0.8	1.5	0.9	1.2	1.1
Delayed breastfeeding	1.0	0.9	1.1	1.5	0.6	0.9	0.9	1.0	0.7	0.6	1.4	0.9	0.9	0.5	0.8	1.0	1.2	0.8	1.2	1.0	1.3	4.1	1.0	0.9	1.2	1.4	0.9	2.2	1.2	1.5	1.2	1.1	0.9	0.9	0.7
FP need unsatisfied	1.0	1.2	1.0	1.3	0.8	0.8	0.8	0.9	0.8	0.8	1.2	1.0	0.9	1.0	1.4	0.2	0.9	1.1	0.8	0.7	0.7	0.4	0.9	1.0	1.3	1.0	1.2	2.2	0.8	0.9	1.1	0.9	0.7	1.1	0.3
No vit A supplement	1.3	1.3	1.0	0.9	0.7	1.2	0.7	1.4	1.6	1.2	1.4	1.0	1.1	2.5	1.0	3.1	1.3	0.8	0.7	1.7	0.5	0.4	0.6	1.3	0.5	3.2	0.8	1.3	0.9	0.9	1.0	1.3	0.7	1.1	0.5
No iodized salt	1.3	0.6	2.3	1.0	0.8	0.8	1.1	0.8	1.0	1.2	1.5	1.0	0.5	0.9	0.5	0.2	0.1	3.8	0.9	1.0	0.8	0.5	2.1	0.8	0.6	4.2	0.7	0.9	0.7	1.5	1.0	0.9	1.5	1.5	2.3
Unsafe water	1.1	0.9	1.5	0.7	1.1	0.9	1.0	1.3	1.1	0.8	0.8	1.1	1.0	0.6	0.9	1.0	0.9	1.4	0.6	0.8	1.2	0.6	1.1	1.0	2.0	0.9	1.2	0.6	0.8	0.9	1.1	1.0	1.3	1.1	1.0
No care seeking for susp pneu	1.7	1.2	1.2	1.0	1.0	0.9	1.0	1.9	2.8	1.0	0.8	0.8	1.0	10.7	1.1	1.9	2.7	0.7	1.2	0.9	1.1	4.4	1.4	1.0	1.0	1.0	1.4	0.4	1.0	1.0	1.0	0.8	1.1	1.0	0.9

Label: odds ratios

>=4.0 [2.0, 4.0) [1.3, 2.0) [1.1, 1.3) [1.0, 1.1) <1.0

Note:

1. BJ= Benin, BF= Burkina Faso, BI= Burundi, CM=Cameroon, CI=Côte d'Ivoire, CD=The Democratic Republic of the Congo, ET=Ethiopia, GM=Gambia, GH=Ghana, GN=Guinea, HT=Haiti, IN=India, KE=Kenya, KG=Kyrgyzstan, KM=Comoros, LS=Lesotho, LR=Liberia, MW=Malawi, ML=Mali, MZ=Mozambique, MM=Myanmar, NA=Namibia, NP=Nepal, NE=Niger, PE=Peru, ST= São Tomé and Príncipe, SL=Sierra Leone, SZ=Swaziland, TD=Chad, TJ=Tajikistan, TZ=Tanzania, TG=Togo, UG=Uganda, ZM=Zambia, ZW=Zimbabwe.
2. Short maternal statue: maternal height <145cm; low maternal BMI: maternal BMI <18.5 kg/m^2; child marriage: mother's age at marriage <18 years old; delayed breastfeeding: child was not initially breastfed within one hour after born; infectious disease: child was caught by infectious diseases two weeks prior to the survey.
3. Abbreviations - HH: household, BMI: body mass index; SBA: skilled birth attendant; ANC: antenatal care; ORT: oral rehydration therapy; FP: family planning; vit: vitamin; susp pneu: suspected pneumonia.

© 2020 Li Z et al. *JAMA Network Open.*

eFigure 7. Relative Ranking of 23 Factors Associated With Child Anthropometric Failures From Supplementary Analysis of Fully Adjusted Models for the Pooled Sample (n = 188,290)
A) stunting, B) underweight, and C) wasting, odds ratio (OR) and 95% confidence interval (CI)

A) Stunting

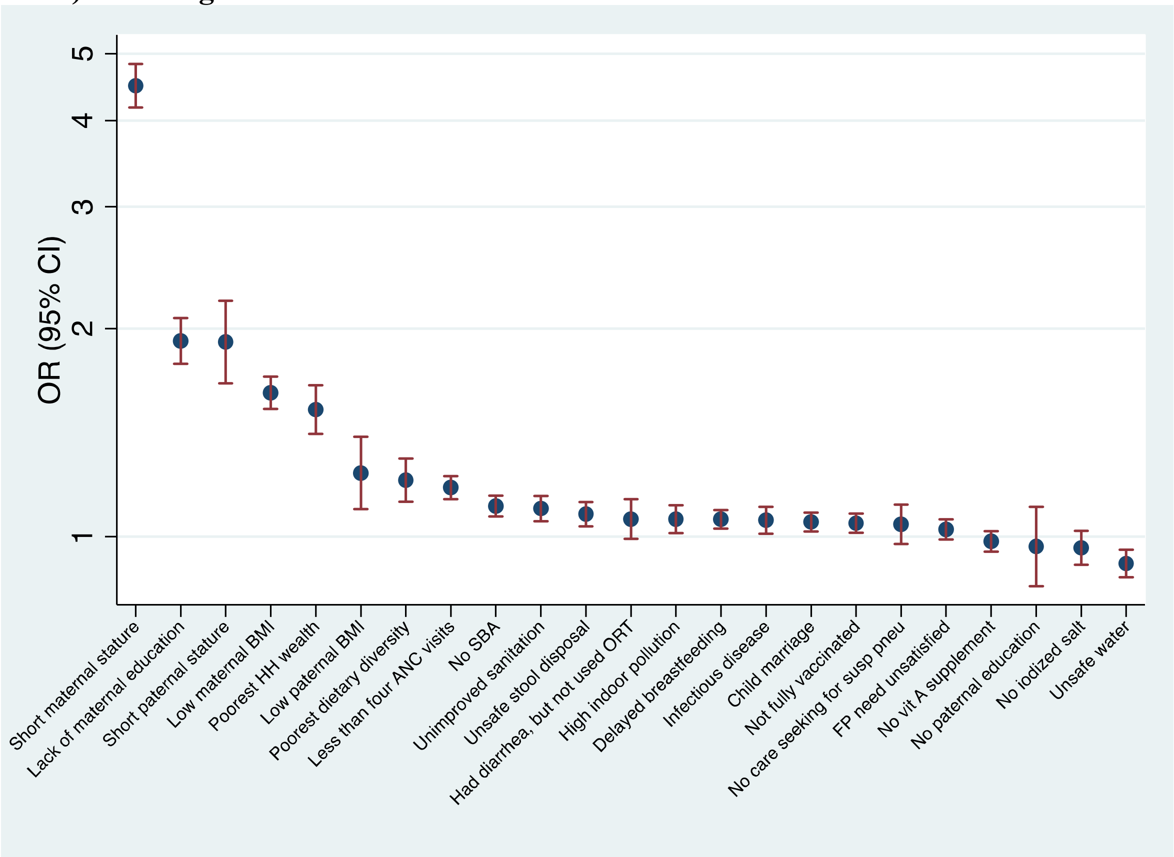

Note:

1. We use logarithmic scale for the y axis.
2. Short maternal statue: maternal height <145cm; low maternal BMI: maternal BMI <18.5 kg/m^2; child marriage: mother's age at marriage <18 years old; delayed breastfeeding: child was not initially breastfed within one hour after born; infectious disease: child was caught by infectious diseases two weeks prior to the survey,
3. Abbreviations - HH: household, BMI: body mass index; SBA: skilled birth attendant; ANC: antenatal care; ORT: oral rehydration therapy; FP: family planning; vit: vitamin; susp pneu: suspected pneumonia.

© 2020 Li Z et al. *JAMA Network Open.*

B) Underweight

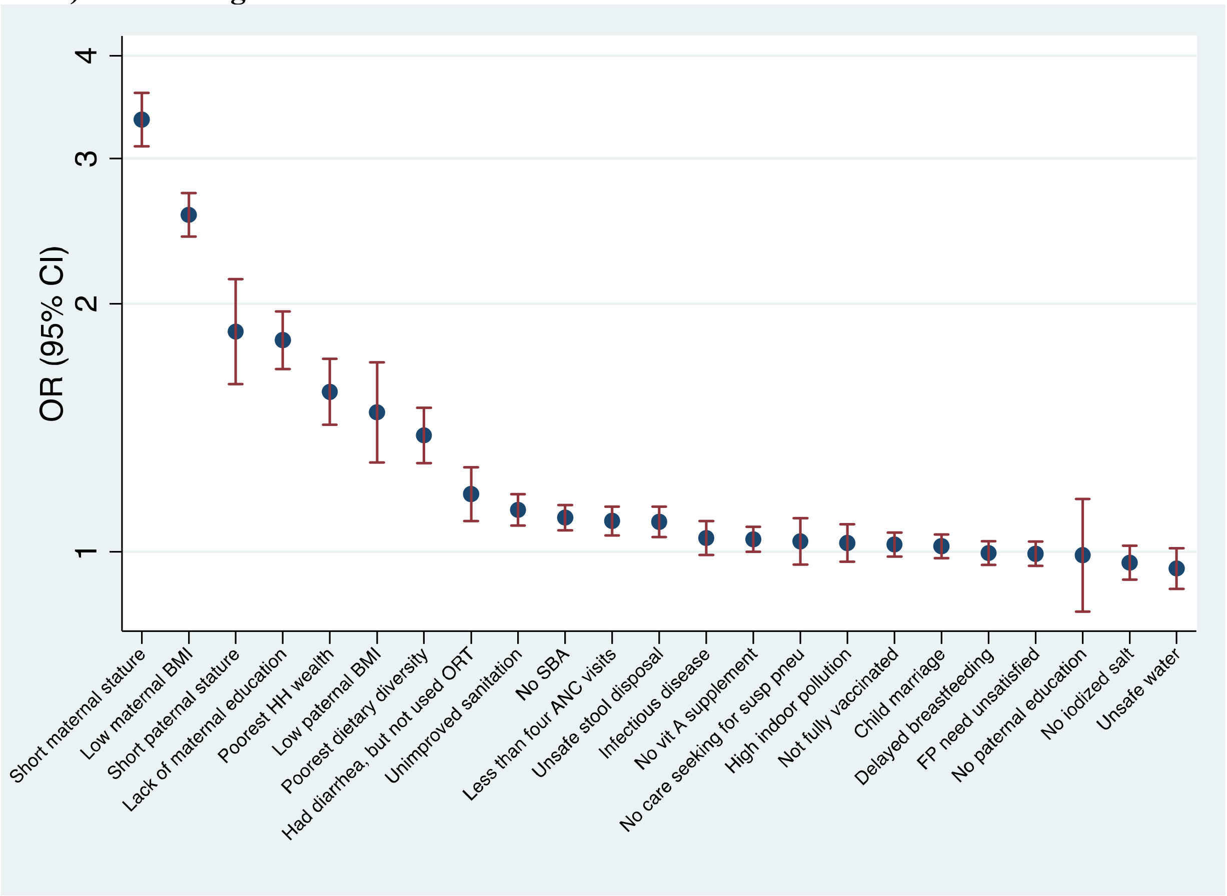

© 2020 Li Z et al. *JAMA Network Open.*

C) Wasting

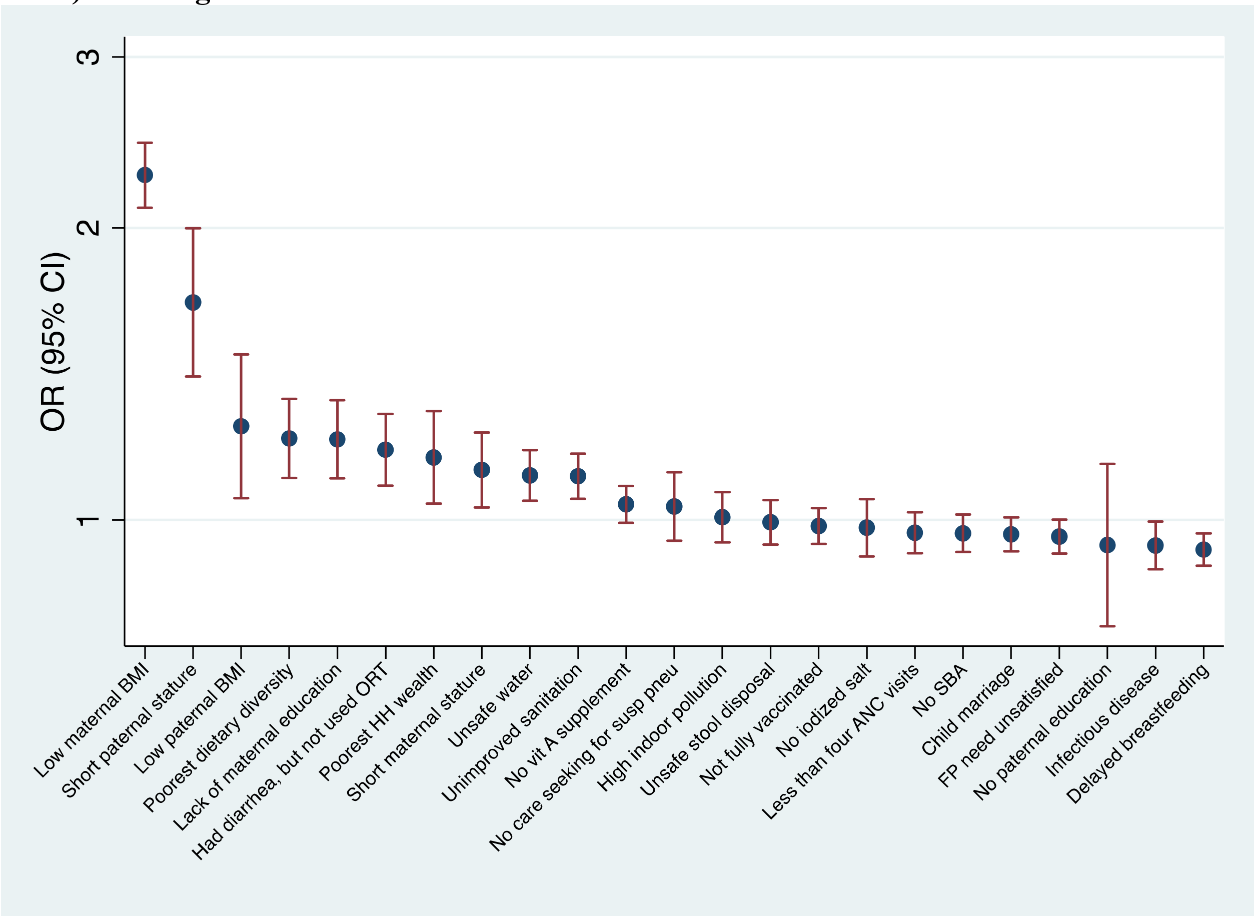

© 2020 Li Z et al. *JAMA Network Open.*

eFigure 8. Country-Specific Ranking of 23 Factors Associated With Child Anthropometric Failures From Supplementary Analysis of Fully Adjusted Models on Stunting, Underweight, and Wasting in 12 Countries

A) Stunting

	ET	GH	IN	LS	LR	NA	NP	ST	SL	SZ	UG	ZW
Short maternal stature	1	1	1	6	1	2	1	3	3	1	1	1
Lack of maternal education	4	2	2	3	2	7	6	13	1	4	2	2
Poorest HH wealth	2	3	5	1	4	6	2	1	4	5	5	4
Low maternal BMI	5	5	4	5	3	8	5	6	5	3	6	3
Poor dietary diversity	3	14	7	15	8	21	22	8	13	22	7	16
No SBA	13	4	12	9	19	17	15	14	15	7	11	9
Less than four ANC visits	20	8	8	7	17	12	13	19	9	18	4	6
Unimprovned sanitation	7	17	11	19	13	13	4	23	17	20	16	8
Had diarrhea, but not used ORT	11	23	9	4	10	3	20	17	22	6	15	23
Unsafe stool disposal	23	7	10	14	16	10	7	5	19	17	8	13
High indoor pollution	19	18	14	22	18	5	14	20	23	21	14	5
Not fully vaccinated	17	6	17	17	15	9	18	7	12	10	21	11
Infectious disease	8	10	16	23	11	11	8	4	18	19	20	7
Child marriage	18	11	13	13	21	22	16	9	14	13	18	10
Delayed breastfeeding	9	16	15	12	9	14	17	12	8	15	17	14
FP need unsatisfied	10	20	18	20	14	16	11	21	7	12	12	17
No vit A supplement	22	9	19	21	12	19	12	11	10	11	19	15
No iodized salt	15	13	20	11	23	23	23	18	6	8	10	22
Unsafe water	16	22	23	16	20	20	9	15	11	16	13	21
No care seeking for susp pneu	14	21	21	10	22	18	19	16	20	14	23	19
Short paternal stature	6	19	3	2	5	1	3	2	2	2	3	20
Low paternal BMI	12	12	6	18	6	4	10	22	16	23	9	12
Lack of paternal education	21	15	22	8	7	15	21	10	21	9	22	18

Label:
Rank 1st Rank 2nd Rank 3rd-4th Rank 5th-7th Rank 8th-12nd Rank 13th-23th

Note:

1. ET=Ethiopia, GH=Ghana, IN=India, LS=Lesotho, LR=Liberia, NA=Namibia, NP=Nepal, ST= São Tomé and Príncipe, SL=Sierra Leone, SZ=Swaziland, UG=Uganda, ZW=Zimbabwe.
2. Short maternal statue: maternal height <145cm; low maternal BMI: maternal BMI <18.5 kg/m2; child marriage: mother's age at marriage <18 years old; delayed breastfeeding: child was not initially breastfed within one hour after born; infectious disease: child was caught by infectious diseases two weeks prior to the survey; short paternal statue: paternal height <155cm; low paternal BMI: paternal BMI <18.5 kg/m2.
3. Abbreviations - HH: household, BMI: body mass index; SBA: skilled birth attendant; ANC: antenatal care; ORT: oral rehydration therapy; FP: family planning; vit: vitamin; susp pneu: suspected pneumonia.

© 2020 Li Z et al. *JAMA Network Open.*

B) Underweight

	ET	GH	IN	LS	LR	NA	NP	ST	SL	SZ	UG	ZW
Short maternal stature	1	3	1	3	1	20	3	5	3	3	1	17
Lack of maternal education	2	1	4	1	13	12	9	21	23	6	2	5
Poorest HH wealth	8	18	6	4	15	6	14	2	15	2	4	20
Low maternal BMI	3	2	2	6	2	8	1	3	1	4	3	1
Poor dietary diversity	5	8	7	22	5	16	8	18	11	15	8	23
No SBA	15	5	12	9	14	10	15	11	17	8	9	13
Less than four ANC visits	4	17	10	12	17	18	10	17	6	22	5	12
Unimprovned sanitation	9	14	9	15	9	19	4	20	8	18	21	9
Had diarrhea, but not used ORT	6	22	8	5	7	4	22	6	5	5	13	22
Unsafe stool disposal	12	7	11	11	12	3	16	10	22	14	7	18
High indoor pollution	22	9	14	16	23	2	6	23	18	21	14	10
Not fully vaccinated	14	12	16	18	16	11	18	7	10	9	17	16
Infectious disease	20	4	15	20	11	22	7	22	21	19	22	8
Child marriage	18	23	17	10	19	21	20	8	19	16	19	11
Delayed breastfeeding	17	21	18	8	10	9	19	12	13	11	11	14
FP need unsatisfied	13	20	19	17	8	14	21	19	12	7	18	7
No vit A supplement	21	13	13	21	18	13	11	4	16	20	10	21
No iodized salt	10	6	21	14	22	17	13	13	14	12	15	6
Unsafe water	19	16	20	13	21	15	12	9	7	10	12	15
No care seeking for susp pneu	16	19	23	19	20	1	17	16	9	17	20	19
Short paternal stature	11	15	3	2	3	7	2	1	2	1	16	2
Low paternal BMI	7	11	5	7	4	5	5	15	20	13	6	3
Lack of paternal education	23	10	22	23	6	23	23	14	4	23	23	4

Label: Rank 1st; Rank 2nd; Rank 3rd-4th; Rank 5th-7th; Rank 8th-12nd; Rank 13th-23th

© 2020 Li Z et al. *JAMA Network Open.*

C) Wasting

	ET	GH	IN	LS	LR	NA	NP	ST	SL	SZ	UG	ZW
Short maternal stature	7	12	7	9	1	11	22	22	23	14	4	3
Lack of maternal education	4	1	5	12	16	2	8	4	4	23	15	13
Poorest HH wealth	5	14	6	15	20	1	23	19	20	4	2	6
Low maternal BMI	1	2	1	8	3	8	3	11	1	2	1	2
Poor dietary diversity	8	23	4	3	6	22	2	21	3	9	16	4
No SBA	10	6	18	7	11	9	17	12	13	21	8	17
Less than four ANC visits	2	4	21	18	13	7	15	1	11	22	21	8
Unimprovned sanitation	21	7	9	2	10	19	5	5	5	11	9	9
Had diarrhea, but not used ORT	9	5	11	11	7	12	13	13	2	1	20	19
Unsafe stool disposal	11	16	13	19	18	6	12	9	8	12	11	18
High indoor pollution	6	20	12	16	14	18	6	20	15	16	22	7
Not fully vaccinated	17	19	14	5	17	14	18	10	14	17	19	20
Infectious disease	19	22	16	20	21	20	7	18	12	13	13	11
Child marriage	12	9	19	17	22	10	20	8	7	20	14	10
Delayed breastfeeding	16	17	22	6	8	4	16	7	16	7	12	16
FP need unsatisfied	18	15	17	22	15	21	19	14	9	6	18	23
No vit A supplement	20	8	10	4	9	17	21	3	17	5	17	21
No iodized salt	13	10	15	21	23	16	4	2	18	8	5	5
Unsafe water	14	13	8	13	19	15	14	17	10	18	6	12
No care seeking for susp pneu	15	3	23	14	4	5	11	15	6	19	7	15
Short paternal stature	22	11	2	10	2	3	10	6	21	15	10	1
Low paternal BMI	3	18	3	23	5	23	1	23	22	3	3	22
Lack of paternal education	23	21	20	1	12	13	9	16	19	10	23	14

Label:

Rank 1st   Rank 2nd   Rank 3rd-4th   Rank 5th-7th   Rank 8th-12nd   Rank 13th-23th

© 2020 Li Z et al. *JAMA Network Open.*

eFigure 9. Country-Specific Odds Ratios for 23 Factors Associated With Child Anthropometric Failures From Supplementary Analysis of Fully Adjusted Models on Stunting, Underweight, and Wasting in 12 Countries

A) Stunting

	ET	GH	IN	LS	LR	NA	NP	ST	SL	SZ	UG	ZW
Short maternal stature	3.0	6.4	4.4	2.6	3.8	3.6	9.2	3.0	2.3	10.5	11.7	6.1
Lack of maternal education	1.8	4.0	1.9	6.9	2.7	2.1	1.4	1.1	3.5	3.7	4.8	4.9
Poorest HH wealth	2.1	1.8	1.4	20.8	2.0	2.2	3.3	6.5	1.6	3.0	2.2	1.8
Low maternal BMI	1.6	1.5	1.6	4.0	2.1	1.9	1.5	2.1	1.4	5.0	1.4	2.1
Poor dietary diversity	2.0	1.0	1.2	0.9	1.2	0.8	0.7	2.0	1.1	0.6	1.3	1.0
No SBA	1.0	1.8	1.1	1.6	0.9	1.1	1.0	1.1	1.0	1.8	1.1	1.2
Less than four ANC visits	0.9	1.2	1.2	2.4	1.0	1.4	1.0	0.6	1.1	1.0	3.4	1.4
Unimprovned sanitation	1.2	0.8	1.1	0.7	1.1	1.4	1.6	0.4	0.9	0.7	0.9	1.2
Had diarrhea, but not used ORT	1.1	0.5	1.1	6.5	1.2	3.0	0.7	0.9	0.4	2.5	0.9	0.7
Unsafe stool disposal	0.7	1.3	1.1	0.9	1.0	1.6	1.2	2.2	0.9	1.0	1.2	1.0
High indoor pollution	0.9	0.8	1.1	0.5	1.0	2.4	1.0	0.6	0.3	0.7	1.0	1.4
Not fully vaccinated	1.0	1.4	1.0	0.7	1.1	1.8	0.8	2.0	1.1	1.5	0.8	1.1
Infectious disease	1.1	1.1	1.1	0.5	1.1	1.5	1.2	2.5	0.9	0.8	0.8	1.3
Child marriage	0.9	1.1	1.1	1.0	0.8	0.6	0.9	1.4	1.0	1.3	0.9	1.2
Delayed breastfeeding	1.1	0.8	1.1	1.0	1.2	1.4	0.9	1.2	1.2	1.1	0.9	1.0
FP need unsatisfied	1.1	0.8	1.0	0.6	1.1	1.2	1.1	0.6	1.2	1.3	1.0	1.0
No vit A supplement	0.8	1.1	1.0	0.6	1.1	1.0	1.1	1.2	1.1	1.5	0.8	1.0
No iodized salt	1.0	1.0	1.0	1.3	0.6	0.5	0.6	0.6	1.4	1.7	1.1	0.9
Unsafe water	1.0	0.7	0.9	0.8	0.8	0.8	1.2	1.0	1.1	1.1	1.0	0.9
No care seeking for susp pneu	1.0	0.8	0.9	1.4	0.8	1.1	0.8	1.0	0.8	1.2	0.7	1.0
Short paternal stature	1.6	0.8	1.9	11.5	1.9	4.9	2.7	4.9	2.7	5.1	3.9	0.9
Low paternal BMI	1.1	1.0	1.3	0.7	1.7	2.5	1.1	0.6	1.0	0.5	1.1	1.0
Lack of paternal education	0.9	0.9	0.9	1.7	1.6	1.3	0.7	1.3	0.7	1.7	0.7	1.0

Label: odds ratios

>=4.0 | [2.0, 4.0) | [1.3, 2.0) | [1.1, 1.3) | [1.0, 1.1) | <1.0

Note:

1. ET=Ethiopia, GH=Ghana, IN=India, LS=Lesotho, LR=Liberia, NA=Namibia, NP=Nepal, ST= São Tomé and Príncipe, SL=Sierra Leone, UG=Uganda ZW=Zimbabwe.
2. Short maternal statue: maternal height <145cm; low maternal BMI: maternal BMI <18.5 kg/m2; child marriage: mother's age at marriage <18 years old; delayed breastfeeding: child was not initially breastfed within one hour after born; infectious disease: child was caught by infectious diseases two weeks prior to the survey; short paternal statue: paternal height <155cm; low paternal BMI: paternal BMI <18.5 kg/m2.
3. Abbreviations - HH: household, BMI: body mass index; SBA: skilled birth attendant; ANC: antenatal care; ORT: oral rehydration therapy; FP: family planning; vit: vitamin; susp pneu: suspected pneumonia.

© 2020 Li Z et al. *JAMA Network Open.*

B) Underweight

	ET	GH	IN	LS	LR	NA	NP	ST	SL	SW	UG	ZW
Short maternal stature	4.1	2.5	3.4	6.8	6.4	0.5	2.4	2.9	2.0	5.4	7.1	0.9
Lack of maternal education	2.9	13.7	1.8	53.6	1.1	1.3	1.2	0.7	0.5	2.5	6.9	1.7
Poorest HH wealth	1.7	0.9	1.5	6.1	1.1	2.3	1.1	6.9	1.2	7.7	2.8	0.7
Low maternal BMI	2.7	4.2	2.5	2.5	5.2	1.6	3.5	5.9	2.3	3.7	3.2	6.3
Poor dietary diversity	1.9	1.3	1.4	0.3	1.6	0.8	1.4	1.0	1.3	0.8	1.4	0.5
No SBA	1.0	1.5	1.1	1.1	1.1	1.4	1.0	1.4	1.0	1.4	1.3	1.2
Less than four ANC visits	2.2	0.9	1.1	0.9	0.9	0.7	1.2	1.0	1.5	0.2	1.5	1.2
Unimprovned sanitation	1.3	1.0	1.1	0.8	1.4	0.5	1.8	0.8	1.4	0.7	0.8	1.3
Had diarrhea, but not used ORT	1.8	0.6	1.2	2.6	1.5	3.6	0.8	2.6	1.6	3.7	1.0	0.7
Unsafe stool disposal	1.1	1.3	1.1	1.0	1.2	4.0	0.9	1.4	0.9	1.0	1.4	0.9
High indoor pollution	0.7	1.2	1.0	0.7	0.1	4.5	1.5	0.4	1.0	0.3	1.0	1.3
Not fully vaccinated	1.0	1.0	1.0	0.6	1.0	1.3	0.9	1.8	1.3	1.3	0.9	1.0
Infectious disease	0.9	1.5	1.0	0.5	1.2	0.2	1.4	0.7	0.9	0.7	0.7	1.3
Child marriage	0.9	0.6	1.0	1.0	0.9	0.5	0.9	1.6	1.0	0.8	0.9	1.3
Delayed breastfeeding	1.0	0.7	1.0	1.3	1.3	1.5	0.9	1.2	1.2	1.1	1.1	1.0
FP need unsatisfied	1.0	0.8	1.0	0.7	1.4	1.0	0.8	0.8	1.3	1.8	0.9	1.6
No vit A supplement	0.8	1.0	1.0	0.5	0.9	1.2	1.2	3.0	1.0	0.5	1.1	0.7
No iodized salt	1.2	1.3	0.9	0.8	0.3	0.7	1.1	1.1	1.2	1.0	1.0	1.7
Unsafe water	0.9	1.0	1.0	0.9	0.7	0.9	1.1	1.5	1.5	1.1	1.1	1.0
No care seeking for susp pneu	1.0	0.8	0.9	0.6	0.7	4.6	0.9	1.0	1.4	0.7	0.8	0.9
Short paternal stature	1.1	1.0	1.9	8.1	2.2	1.9	3.0	47.0	2.2	70.4	0.9	2.8
Low paternal BMI	1.7	1.1	1.6	1.8	2.1	2.4	1.5	1.0	0.9	1.0	1.4	1.8
Lack of paternal education	0.5	1.1	0.9	0.3	1.5	0.1	0.8	1.0	1.8	0.0	0.5	1.8

Label: odds ratios
>=4.0 [2.0, 4.0) [1.3, 2.0) [1.1, 1.3) [1.0, 1.1) <1.0

© 2020 Li Z et al. *JAMA Network Open.*

C) Wasting

	ET	GH	IN	LS	LR	NA	NP	ST	SL	SW	UG	ZW
Short maternal stature	1.70	1.00	1.15	1.22	4.73	1.00	0.40	0.38	0.24	1.00	2.49	3.80
Lack of maternal education	2.01	182.46	1.21	1.00	0.95	6.92	1.65	1.65	1.86	0.05	0.86	1.00
Poorest HH wealth	1.87	0.87	1.16	0.80	0.76	43.61	0.36	0.78	0.73	14.68	4.09	1.80
Low maternal BMI	3.74	4.22	2.28	1.24	2.78	1.80	2.10	1.13	2.34	135.21	5.89	4.73
Poor dietary diversity	1.63	0.18	1.22	4.08	2.15	0.23	2.50	0.71	1.93	2.13	0.83	2.63
No SBA	1.32	1.85	0.96	1.49	1.06	1.31	1.00	1.06	1.09	0.18	1.07	0.65
Less than four ANC visits	2.54	2.73	0.94	0.27	1.04	2.42	1.05	4.42	1.17	0.11	0.51	1.34
Unimprovned sanitation	0.72	1.56	1.10	20.05	1.15	0.39	1.82	1.62	1.63	2.08	1.01	1.24
Had diarrhea, but not used ORT	1.58	2.52	1.02	1.00	2.00	0.92	1.25	1.05	2.10	276.22	0.59	0.53
Unsafe stool disposal	1.18	0.72	1.00	0.20	0.90	3.08	1.25	1.28	1.29	1.63	0.94	0.65
High indoor pollution	1.87	0.32	1.01	0.53	1.00	0.40	1.77	0.77	1.00	0.91	0.48	1.76
Not fully vaccinated	0.90	0.48	0.99	2.50	0.91	0.84	0.87	1.21	1.05	0.83	0.64	0.52
Infectious disease	0.77	0.25	0.97	0.20	0.59	0.36	1.76	0.79	1.10	1.35	0.87	1.00
Child marriage	1.16	1.14	0.96	0.34	0.58	1.23	0.84	1.39	1.32	0.23	0.86	1.09
Delayed breastfeeding	0.94	0.68	0.93	1.86	1.21	4.26	1.04	1.42	0.93	2.23	0.93	0.71
FP need unsatisfied	0.83	0.76	0.96	0.10	0.99	0.30	0.84	1.04	1.28	2.83	0.68	0.34
No vit A supplement	0.73	1.53	1.05	3.13	1.18	0.46	0.60	3.52	0.84	3.54	0.74	0.50
No iodized salt	1.11	1.02	0.99	0.13	0.09	0.52	2.05	3.85	0.75	2.14	1.52	2.22
Unsafe water	1.05	1.00	1.11	0.89	0.88	0.68	1.09	0.94	1.23	0.64	1.36	1.00
No care seeking for susp pneu	1.00	3.14	0.85	0.86	2.53	3.91	1.39	1.00	1.50	0.24	1.13	0.90
Short paternal stature	0.44	1.00	1.74	1.00	3.52	6.86	1.42	1.46	0.64	1.00	1.00	6.76
Low paternal BMI	2.51	0.67	1.27	0.09	2.37	0.19	2.90	0.16	0.58	30.16	3.63	0.42
Lack of paternal education	0.27	0.26	0.95	32.41	1.05	0.90	1.48	1.00	0.74	2.08	0.22	1.00

Label: odds ratios

>=4.0   [2.0, 4.0)   [1.3, 2.0)   [1.1, 1.3)   [1.0, 1.1)   <1.0

© 2020 Li Z et al. *JAMA Network Open.*

eFigure 10. Relative Ranking of 20 Factors Associated With Child Anthropometric Failures From Fully Adjusted Models for Pooled Sample, Stratified by Children's Age
A) stunting, B) underweight, and C) wasting, odds ratio (OR) and 95% confidence interval (CI)

A) Stunting

**Children 12-23 months old, n=76,862**	**Children 24-59 months old, n=222,491**

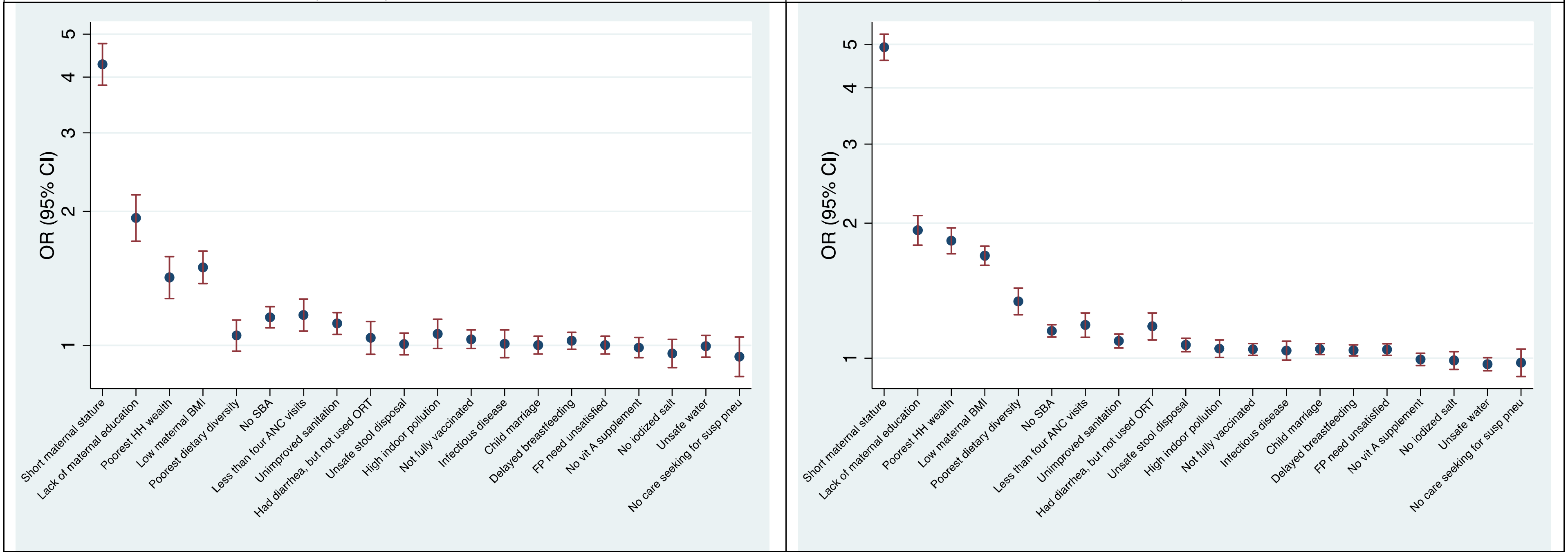

Note:

1. We use logarithmic scale for the y axis.
2. Short maternal statue: maternal height <145cm; low maternal BMI: maternal BMI <18.5 kg/m^2; child marriage: mother's age at marriage <18 years old; delayed breastfeeding: child was not initially breastfed within one hour after born; infectious disease: child was caught by infectious diseases two weeks prior to the survey.
3. Abbreviations - HH: household, BMI: body mass index; SBA: skilled birth attendant; ANC: antenatal care; ORT: oral rehydration therapy; FP: family planning; vit: vitamin; susp pneu: suspected pneumonia.

© 2020 Li Z et al. *JAMA Network Open.*

B) Underweight

Children 12-23 months old, n=76,862

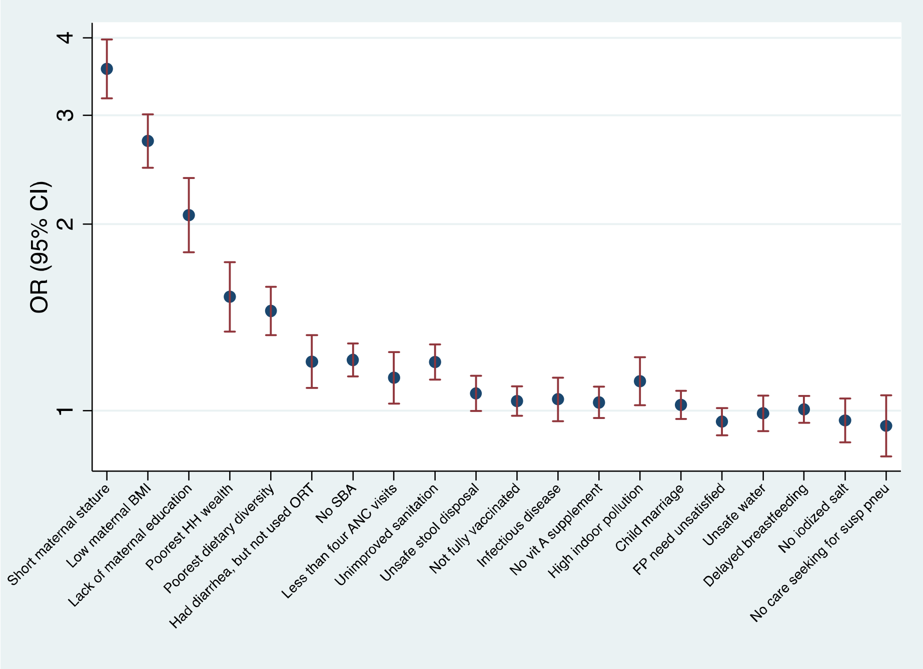

Children 24-59 months old, n=222,491

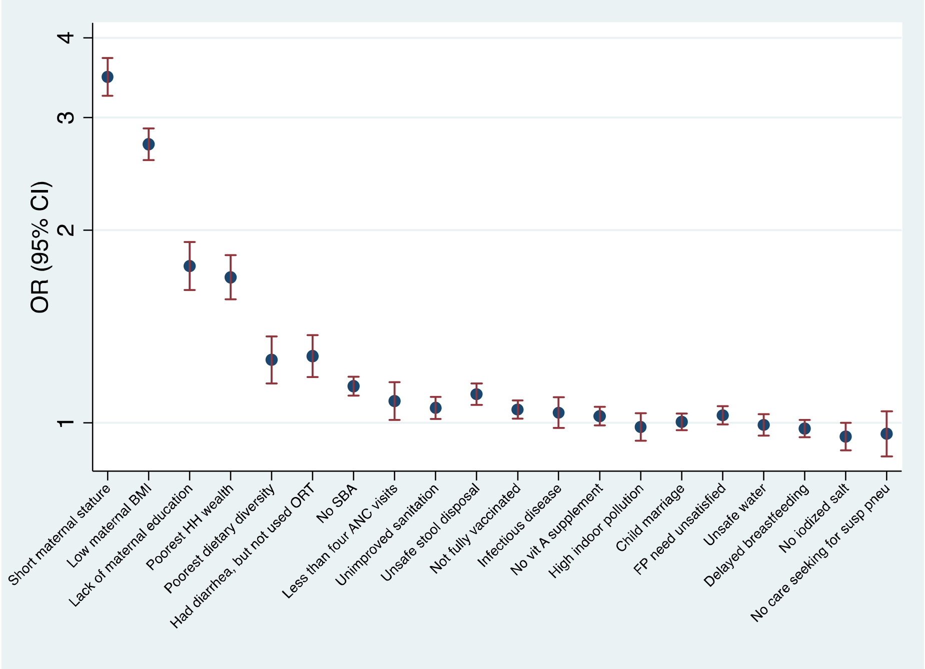

© 2020 Li Z et al. *JAMA Network Open.*

C) Wasting

Children 12-23 months old, n=76,862 | **Children 24-59 months old, n=222,491**

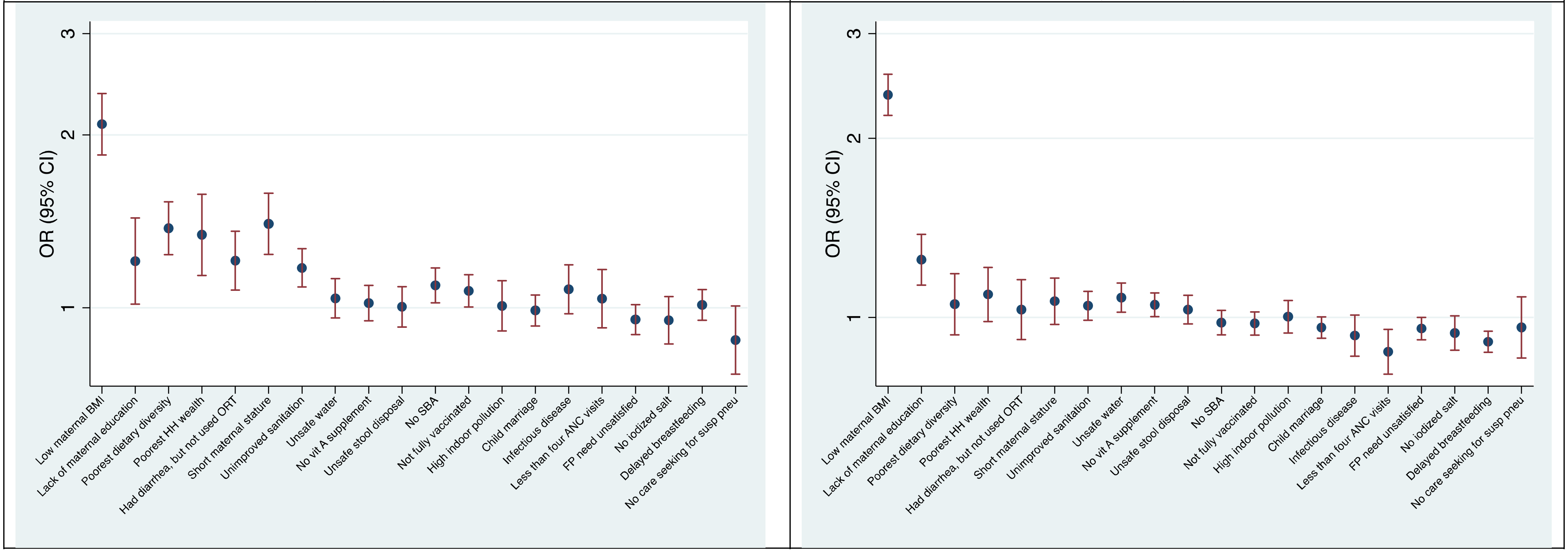

© 2020 Li Z et al. *JAMA Network Open.*

eFigure 11. Country-Specific Ranking of 20 Factors Associated With Child Anthropometric Failures From Fully Adjusted Models, Stratified by Children's Age
A) stunting among children 12-23 months old, B) stunting among children 24-59 months old, C) underweight among children 12-23 months old, D) underweight among children 24-59 months old, E) wasting among children 12-23 months old, and F) wasting among children 24-59 months old

A) Stunting, children 12-23 months old, total n=76,862

	BJ	BF	BI	CM	CI	CD	ET	GM	GH	GN	HT	IN	KE	KG	KM	LS	LR	MW	ML	MZ	MM	NA	NP	NE	PE	ST	SL	SZ	TD	TJ	TZ	TG	UG	ZM	ZW
Short maternal stature	1	5	1	13	3	2	1	1	1	8	2	1	1	3	13	5	2	1	13	2	1	10	1	2	1	1	4	3	1	4	1	14	3	1	2
Lack of maternal education	3	1	18	1	8	1	2	20	13	4	6	2	3	16	3	7	18	3	2	1	10	20	6	4	3	9	14	4	16	1	19	20	1	5	1
Poorest HH wealth	8	11	2	2	19	4	3	16	3	2	1	4	2	8	2	1	1	7	1	4	20	1	2	20	2	3	12	2	18	18	3	7	17	12	3
Low maternal BMI	5	3	3	5	2	5	4	3	20	3	7	3	13	1	1	2	17	2	3	15	2	7	20	1	5	7	16	10	7	6	5	2	4	2	4
Poor dietary diversity	18	4	6	12	18	16	6	8	15	9	3	11	20	2	16	6	20	15	5	9	19	9	3	3	8	2	19	18	3	5	2	9	19	11	20
No SBA	6	6	5	8	17	10	7	12	4	15	10	7	6	11	18	8	11	5	9	5	7	17	13	16	4	13	5	12	5	15	15	11	12	3	8
Less than four ANC visits	2	20	20	4	1	3	20	4	2	1	17	6	4	19	7	4	7	20	18	6	4	2	9	7	9	19	11	19	20	8	4	1	2	7	5
Unimproved sanitation	13	8	15	19	15	11	5	5	19	5	8	5	11	9	9	17	12	12	20	10	18	12	12	15	18	15	6	13	10	7	12	17	8	6	7
Had diarrhea, but not used ORT	7	19	7	10	4	17	11	11	18	19	16	12	7	12	5	3	16	4	19	8	13	4	18	14	10	6	20	9	2	2	6	19	11	18	19
Unsafe stool disposal	16	7	4	7	9	15	16	6	8	17	9	13	16	17	14	16	9	6	10	17	16	3	4	6	19	11	8	15	8	3	10	10	5	9	14
High indoor pollution	4	16	13	3	6	19	19	18	7	20	19	9	8	6	19	18	14	19	7	3	5	16	16	18	16	20	13	20	4	14	8	13	20	4	10
Not fully vaccinated	12	12	16	11	16	14	15	13	9	18	13	10	9	10	4	12	4	14	16	16	14	5	15	13	13	5	7	5	9	13	14	8	15	15	17
Infectious disease	10	2	10	16	20	8	12	9	16	6	12	15	17	20	8	15	19	17	6	11	17	6	8	9	20	8	2	16	19	20	17	18	9	10	6
Child marriage	11	15	8	20	11	9	18	15	14	16	20	8	15	14	11	13	8	16	17	14	8	18	14	8	14	10	17	6	13	17	20	16	16	14	12
Delayed breastfeeding	15	17	14	17	13	7	8	10	10	11	4	14	10	7	12	9	5	10	11	12	6	11	17	11	17	16	1	11	17	9	16	15	7	8	9
FP need unsatisfied	9	9	9	18	14	18	9	7	17	14	5	16	18	4	10	20	6	13	15	18	12	15	5	10	7	17	10	14	14	12	13	4	14	19	11
No vit A supplement	20	14	11	15	5	6	17	2	11	7	18	17	19	15	6	19	13	11	12	19	3	19	11	5	6	4	3	1	6	16	18	6	10	17	13
No iodized salt	17	18	19	6	7	20	10	17	5	13	15	20	12	18	15	11	15	9	8	13	11	13	7	17	12	18	15	17	15	19	7	12	13	20	15
Unsafe water	14	13	12	9	10	13	13	14	12	10	14	18	5	13	20	10	10	18	14	7	9	14	10	12	15	14	9	8	11	10	9	5	6	16	18
No care seeking for susp pneu	19	10	17	14	12	12	14	19	6	12	11	19	14	5	17	14	3	8	4	20	15	8	19	19	11	12	18	7	12	11	11	3	18	13	16

Label:
- Rank 1st
- Rank 2nd
- Rank 3rd-4th
- Rank 5th-7th
- Rank 8th-12nd
- Rank 13th-20th

Note:
1. BJ= Benin, BF= Burkina Faso, BI= Burundi, CM=Cameroon, CI=Côte d'Ivoire, CD=The Democratic Republic of the Congo, ET=Ethiopia, GM=Gambia, GH=Ghana, GN=Guinea, HT=Haiti, IN=India, KE=Kenya, KG=Kyrgyzstan, KM=Comoros, LS=Lesotho, LR=Liberia, MW=Malawi, ML=Mali, MZ=Mozambique, MM=Myanmar, NA=Namibia, NP=Nepal, NE=Niger, PE=Peru, ST= São Tomé and Príncipe, SL=Sierra Leone, SZ=Swaziland, TD=Chad, TJ=Tajikistan, TZ=Tanzania, TG=Togo, UG=Uganda, ZM=Zambia, ZW=Zimbabwe.
2. Short maternal statue: maternal height <145cm; low maternal BMI: maternal BMI <18.5 kg/m2; child marriage: mother's age at marriage <18 years old; delayed breastfeeding: child was not initially breastfed within one hour after born; infectious disease: child was caught by infectious diseases two weeks prior to the survey.
3. Abbreviations - HH: household, BMI: body mass index; SBA: skilled birth attendant; ANC: antenatal care; ORT: oral rehydration therapy; FP: family planning; vit: vitamin; susp pneu: suspected pneumonia.

© 2020 Li Z et al. *JAMA Network Open.*

B) Stunting, children 24-59 months old, total n=222,491

	BJ	BF	BI	CM	CI	CD	ET	GM	GH	GN	HT	IN	KE	KG	KM	LS	LR	MW	ML	MZ	MM	NA	NP	NE	PE	ST	SL	SZ	TD	TJ	TZ	TG	UG	ZM	ZW
Short maternal stature	1	1	1	1	2	2	1	18	2	19	1	1	1	1	6	6	1	1	3	1	1	6	1	4	1	2	2	1	7	1	1	1	1	1	1
Lack of maternal education	5	2	2	4	1	11	4	6	1	7	6	2	10	18	1	2	4	2	1	5	9	15	5	2	3	14	1	3	1	11	2	8	3	3	2
Poorest HH wealth	2	3	3	2	4	3	3	1	4	3	2	4	2	11	9	1	3	4	2	2	2	1	2	10	2	1	4	6	12	5	3	2	2	2	5
Low maternal BMI	4	4	4	5	3	4	5	17	3	18	3	3	3	2	3	4	2	5	6	3	3	2	3	8	4	7	3	2	3	2	4	3	6	4	3
Poor dietary diversity	13	7	16	3	19	5	2	4	11	1	20	5	5	13	20	19	5	13	4	7	8	20	19	9	10	6	5	20	5	3	6	7	5	16	12
No SBA	7	6	9	19	15	16	19	5	5	4	11	9	4	20	11	9	15	11	11	12	4	7	11	11	5	10	15	4	8	6	12	13	7	19	8
Less than four ANC visits	3	14	15	6	18	20	6	10	14	15	4	7	7	17	12	7	13	20	5	6	7	17	17	1	20	18	11	7	2	8	20	20	4	20	15
Unimproved sanitation	16	16	7	20	7	19	8	3	13	14	8	12	6	9	17	15	10	12	16	9	10	8	4	13	6	20	16	19	20	14	8	17	14	14	11
Had diarrhea, but not used ORT	8	5	17	14	20	10	13	19	19	2	9	6	13	16	4	3	9	17	7	10	19	3	18	19	17	19	19	18	6	4	7	14	15	7	20
Unsafe stool disposal	10	10	8	11	10	8	20	7	8	5	14	8	19	7	16	11	14	16	8	19	6	11	7	5	8	3	17	15	9	9	13	5	8	15	13
High indoor pollution	6	17	14	13	5	1	9	20	17	20	5	14	20	8	19	18	12	3	20	4	20	4	9	3	13	13	20	16	4	7	5	4	10	5	4
Not fully vaccinated	19	9	5	15	6	18	15	2	6	6	10	15	16	10	10	17	16	9	15	15	13	9	16	12	12	5	14	8	15	19	15	6	18	10	6
Infectious disease	15	18	12	8	9	17	7	14	7	13	18	10	14	3	18	20	6	18	19	8	12	10	8	6	9	4	18	17	10	12	16	9	19	12	9
Child marriage	11	13	11	16	12	9	12	9	9	16	12	13	8	12	13	12	18	14	13	14	11	18	14	14	11	8	8	11	16	17	19	10	16	6	10
Delayed breastfeeding	12	12	10	18	16	12	11	11	15	17	17	11	9	4	14	10	7	6	17	18	18	14	12	16	14	9	10	12	17	18	17	12	13	13	17
FP need unsatisfied	9	11	18	10	14	15	10	8	16	10	15	16	17	5	8	13	11	7	12	13	14	13	15	17	18	16	7	9	11	15	18	15	9	8	16
No vit A supplement	17	20	6	9	11	7	17	13	10	9	16	17	18	14	15	14	8	15	9	20	16	5	13	7	7	17	12	10	19	20	9	11	17	17	7
No iodized salt	20	19	20	7	8	14	18	12	12	8	13	18	15	15	2	8	20	19	14	16	15	19	20	15	16	15	6	5	13	16	10	18	11	18	19
Unsafe water	18	15	19	12	17	13	16	16	18	11	19	20	11	6	7	16	17	8	10	11	5	16	6	18	19	11	13	13	18	13	11	16	12	9	14
No care seeking for susp pneu	14	8	13	17	13	6	14	15	20	12	7	19	12	19	5	5	19	10	18	17	17	12	10	20	15	12	9	14	14	10	14	19	20	11	18

Label:

Rank 1st   Rank 2nd   Rank 3rd-4th   Rank 5th-7th   Rank 8th-12nd   Rank 13th-20th

© 2020 Li Z et al. *JAMA Network Open.*

C) Underweight, children 12-23 months old, total n=76,862

	BJ	BF	BI	CM	CI	CD	ET	GM	GH	GN	HT	IN	KE	KG	KM	LS	LR	MW	ML	MZ	MM	NA	NP	NE	PE	ST	SL	SZ	TD	TJ	TZ	TG	UG	ZM	ZW
Short maternal stature	2	2	2	5	12	5	1	10	2	2	1	1	1	16	6	9	1	2	20	1	1	8	2	20	2	12	3	15	20	14	1	1	3	1	12
Lack of maternal education	7	5	20	4	3	1	2	18	13	6	18	3	3	9	13	11	3	8	1	2	5	3	9	8	11	5	20	4	6	4	12	9	2	5	4
Poorest HH wealth	20	19	3	19	16	18	3	16	3	4	3	4	2	7	2	8	6	9	5	14	18	9	8	17	13	3	1	1	10	15	4	2	4	6	5
Low maternal BMI	1	1	1	2	1	2	7	4	1	3	2	2	7	4	1	12	2	1	2	3	2	12	5	1	1	1	12	12	1	2	3	4	1	2	1
Poor dietary diversity	5	9	7	3	8	4	4	2	8	5	6	5	19	2	3	10	4	13	4	5	19	13	3	2	3	13	10	10	4	3	5	12	5	16	20
No SBA	6	4	5	7	15	7	11	8	6	11	19	7	9	15	18	6	12	4	9	15	6	14	12	10	7	8	7	16	7	10	17	5	13	9	6
Less than four ANC visits	19	20	17	9	7	16	10	3	11	8	12	9	4	18	11	14	14	20	18	11	3	2	1	18	18	15	13	20	19	7	14	3	6	3	2
Unimproved sanitation	14	6	12	12	10	11	6	6	16	18	8	6	17	1	7	19	10	14	11	20	17	20	7	9	5	17	8	17	17	8	16	19	18	8	9
Had diarrhea, but not used ORT	9	18	6	15	5	13	5	7	10	9	17	8	6	17	8	1	7	3	17	4	16	4	20	3	8	4	2	13	5	1	19	20	9	19	19
Unsafe stool disposal	17	8	8	17	6	19	8	5	5	19	7	12	10	8	15	4	19	18	8	9	13	7	16	4	15	18	15	6	3	5	6	14	8	10	16
High indoor pollution	3	3	19	1	2	14	12	20	12	1	13	10	5	3	14	17	16	10	19	8	4	11	4	13	16	20	16	19	8	16	2	6	12	4	18
Not fully vaccinated	8	16	15	6	13	17	17	19	15	16	11	15	8	19	5	18	15	5	10	12	11	5	13	11	9	14	9	5	2	18	8	10	15	14	14
Infectious disease	10	7	18	13	18	20	14	13	14	13	10	14	13	20	10	20	8	16	3	10	7	18	18	7	10	19	6	18	9	20	11	7	17	13	13
Child marriage	11	13	14	18	19	10	18	14	20	7	20	13	20	12	19	7	13	6	13	13	10	19	11	5	14	10	18	9	12	6	20	11	19	11	7
Delayed breastfeeding	15	17	10	8	11	15	16	15	19	14	9	16	15	10	9	2	5	19	14	16	9	1	15	15	6	6	4	8	18	9	10	17	10	7	8
FP need unsatisfied	4	10	16	14	17	12	15	12	17	20	4	19	18	11	4	16	9	15	15	18	15	16	14	12	17	7	17	3	16	19	9	8	16	20	10
No vit A supplement	13	15	13	10	9	6	20	1	18	10	16	11	16	6	12	15	17	7	12	6	12	17	17	6	4	2	19	14	15	12	18	15	11	12	17
No iodized salt	16	12	4	20	20	3	9	17	9	12	5	18	14	5	16	3	18	17	7	19	14	6	6	19	20	16	11	2	13	11	7	13	14	18	3
Unsafe water	12	11	9	11	14	8	19	9	7	15	15	17	11	13	17	13	20	12	16	7	8	15	10	14	19	9	5	11	14	17	15	16	7	17	15
No care seeking for susp pneu	18	14	11	16	4	9	13	11	4	17	14	20	12	14	20	5	11	11	6	17	20	10	19	16	12	11	14	7	11	13	13	18	20	15	11

Label:
Rank 1st   Rank 2nd   Rank 3rd-4th   Rank 5th-7th   Rank 8th-12nd   Rank 13th-20th

© 2020 Li Z et al. *JAMA Network Open.*

D) Underweight, children 24-59 months old, total n=222,491

	BJ	BF	BI	CM	CI	CD	ET	GM	GH	GN	HT	IN	KE	KG	KM	LS	LR	MW	ML	MZ	MM	NA	NP	NE	PE	ST	SL	SZ	TD	TJ	TZ	TG	UG	ZM	ZW
Short maternal stature	2	1	2	3	4	1	2	2	3	11	1	1	2	12	4	2	2	1	1	2	1	14	2	2	1	2	1	1	8	2	1	3	2	1	2
Lack of maternal education	1	3	5	6	2	19	4	12	1	7	4	3	4	13	1	4	17	17	2	4	19	15	8	4	5	18	3	3	3	20	6	6	1	6	3
Poorest HH wealth	4	4	3	2	6	2	6	5	19	12	15	4	3	17	9	3	3	3	7	3	12	3	6	7	2	1	18	2	7	6	4	19	4	3	19
Low maternal BMI	3	2	1	1	1	3	3	3	2	1	3	2	1	1	2	1	1	2	3	1	2	7	1	1	4	3	2	4	1	1	2	2	3	2	1
Poor dietary diversity	13	15	16	4	14	9	5	19	12	2	19	5	18	14	20	19	14	4	8	5	3	11	18	15	20	14	16	18	9	12	12	9	5	18	20
No SBA	5	8	7	10	15	10	16	6	5	3	11	9	6	11	8	6	9	11	10	20	5	6	12	17	18	12	17	5	5	9	18	12	7	8	12
Less than four ANC visits	6	7	18	5	20	20	1	4	13	19	8	8	13	15	15	16	16	15	6	18	6	19	20	11	13	13	5	20	2	3	20	17	18	4	15
Unimproved sanitation	18	12	15	18	9	14	10	13	7	15	10	10	10	3	19	13	7	19	12	10	9	16	3	14	7	15	6	9	10	10	17	14	19	10	7
Had diarrhea, but not used ORT	8	5	9	7	19	7	7	7	17	4	6	6	9	10	6	7	5	20	4	8	20	2	10	19	8	10	13	8	4	4	5	4	15	16	18
Unsafe stool disposal	15	11	11	9	13	5	12	17	9	10	16	7	12	5	5	5	6	5	9	12	4	5	14	5	11	4	19	12	12	18	8	7	6	19	11
High indoor pollution	14	6	20	20	8	15	20	1	6	6	2	14	5	4	16	14	19	14	5	7	15	1	7	6	12	19	15	19	11	13	3	1	13	9	4
Not fully vaccinated	17	9	4	11	11	16	8	11	8	14	12	13	16	9	3	15	11	10	13	14	13	12	19	16	19	5	8	7	6	17	9	5	12	7	16
Infectious disease	7	13	19	14	5	8	19	20	4	17	20	11	11	2	18	12	8	8	20	9	7	20	4	3	9	17	20	6	16	8	11	15	20	5	6
Child marriage	16	16	13	15	12	11	14	15	18	18	14	15	19	16	13	11	13	12	16	15	8	18	16	12	15	7	12	13	19	15	16	8	11	13	9
Delayed breastfeeding	12	18	12	19	18	17	15	16	15	20	13	17	17	8	12	8	10	6	14	17	17	9	13	10	17	9	14	14	15	14	19	16	10	12	13
FP need unsatisfied	10	10	14	13	7	18	11	14	14	8	17	16	7	7	10	10	4	13	15	11	16	10	17	13	14	20	9	15	14	7	7	10	14	11	5
No vit A supplement	9	19	10	12	10	12	18	9	11	5	7	12	15	6	14	18	12	18	18	19	11	8	5	8	6	6	10	16	18	19	13	13	9	20	17
No iodized salt	20	17	6	8	3	4	9	18	10	9	9	19	20	18	17	9	20	7	19	16	18	17	15	9	3	8	11	11	17	5	10	18	8	17	8
Unsafe water	19	14	17	16	17	13	17	10	16	16	18	18	8	19	7	17	15	9	17	13	10	13	9	18	10	11	7	10	20	16	14	11	17	14	10
No care seeking for susp pneu	11	20	8	17	16	6	13	8	20	13	5	20	14	20	11	20	18	16	11	6	14	4	11	20	16	16	4	17	13	11	15	20	16	15	14

Label:

Rank 1st   Rank 2nd   Rank 3rd-4th   Rank 5th-7th   Rank 8th-12nd   Rank 13th-20th

© 2020 Li Z et al. *JAMA Network Open.*

E) Wasting, children 12-23 months old, total n=76,862

	BJ	BF	BI	CM	CI	CD	ET	GM	GH	GN	HT	IN	KE	KG	KM	LS	LR	MW	ML	MZ	MM	NA	NP	NE	PE	ST	SL	SZ	TD	TJ	TZ	TG	UG	ZM	ZW
Short maternal stature	15	4	18	6	9	2	4	12	11	9	14	2	15	9	2	2	12	1	12	1	9	8	18	3	9	11	19	4	8	14	9	1	3	8	2
Lack of maternal education	17	3	20	4	1	9	20	11	12	6	13	6	3	2	18	12	18	12	1	5	20	1	5	19	14	4	17	6	11	7	3	6	4	13	10
Poorest HH wealth	20	16	7	18	5	19	3	3	3	5	12	3	5	17	6	15	20	14	14	2	1	10	19	1	18	18	18	11	16	11	15	3	1	1	1
Low maternal BMI	1	2	11	2	2	1	7	6	5	3	3	1	10	14	5	9	11	7	4	7	3	4	9	2	1	3	1	12	1	2	7	2	9	6	5
Poor dietary diversity	5	1	13	3	7	11	5	2	18	1	19	4	4	19	1	11	16	6	5	8	7	14	2	17	4	1	4	2	6	12	1	18	16	10	4
No SBA	7	5	1	1	15	7	9	18	13	14	7	9	6	8	15	18	5	4	9	19	4	6	13	16	6	14	12	8	19	9	14	4	13	3	15
Less than four ANC visits	19	20	12	20	20	10	2	1	4	18	5	13	1	15	17	16	6	20	20	20	8	2	4	20	11	2	16	14	13	1	13	20	6	17	3
Unimproved sanitation	11	15	5	5	11	16	19	9	8	12	16	5	16	1	7	5	15	19	6	18	13	20	14	12	7	9	2	20	7	16	20	9	17	11	6
Had diarrhea, but not used ORT	8	10	3	11	12	4	11	17	1	13	18	8	2	11	9	1	1	2	16	11	19	12	3	4	17	5	3	13	20	4	19	12	18	2	9
Unsafe stool disposal	18	6	9	15	10	20	10	7	16	17	10	15	12	16	10	3	14	18	8	12	12	16	6	8	12	16	5	9	4	8	6	17	20	18	13
High indoor pollution	2	18	19	7	3	13	1	20	20	2	15	16	11	18	8	4	10	16	2	3	17	5	11	9	2	13	13	19	2	20	11	19	10	20	18
Not fully vaccinated	6	12	14	8	19	8	18	10	9	11	6	10	7	3	4	10	7	9	15	10	2	11	15	10	5	19	6	16	5	10	5	11	12	9	17
Infectious disease	9	7	16	13	4	5	6	8	19	4	20	11	18	20	11	13	19	15	3	6	5	18	10	5	3	20	10	1	3	19	2	8	5	19	16
Child marriage	12	17	10	14	8	12	8	14	6	7	17	17	19	12	20	20	17	13	11	16	11	13	17	7	10	10	7	15	14	6	17	5	15	7	7
Delayed breastfeeding	10	8	8	9	18	15	15	16	17	20	4	14	13	6	19	14	4	17	13	15	14	9	16	15	13	8	9	5	9	5	4	16	11	4	12
FP need unsatisfied	16	11	17	12	6	17	14	15	14	19	8	19	17	7	3	7	13	5	18	17	16	17	7	13	16	15	15	7	15	17	10	10	8	12	20
No vit A supplement	4	9	15	17	16	3	17	4	2	16	9	12	9	4	14	8	8	8	17	4	18	15	20	6	19	7	20	3	18	13	8	13	14	16	19
No iodized salt	3	19	2	19	17	6	16	19	15	10	1	18	20	13	13	17	2	3	10	13	10	7	1	18	20	6	14	17	10	3	18	7	19	5	11
Unsafe water	14	13	4	16	14	14	12	13	10	15	11	7	8	5	12	19	9	10	19	14	6	19	12	11	8	17	11	18	17	18	16	14	2	15	8
No care seeking for susp pneu	13	14	6	10	13	18	13	5	7	8	2	20	14	10	16	6	3	11	7	9	15	3	8	14	15	12	8	10	12	15	12	15	7	14	14

Label:

Rank 1st   Rank 2nd   Rank 3rd-4th   Rank 5th-7th   Rank 8th-12nd   Rank 13th-20th

© 2020 Li Z et al. *JAMA Network Open.*

F) Wasting, children 24-59 months old, total n=222,491

	BJ	BF	BI	CM	CI	CD	ET	GM	GH	GN	HT	IN	KE	KG	KM	LS	LR	MW	ML	MZ	MM	NA	NP	NE	PE	ST	SL	SZ	TD	TJ	TZ	TG	UG	ZM	ZW
Short maternal stature	2	3	17	13	11	4	13	5	10	6	5	5	12	9	15	11	20	1	20	6	16	12	20	1	2	18	20	5	1	8	2	11	5	1	1
Lack of maternal education	16	9	19	18	1	7	3	9	17	12	7	2	1	19	1	10	2	20	3	5	19	1	6	19	12	9	4	6	6	7	15	2	19	20	8
Poorest HH wealth	19	7	2	1	18	1	8	19	20	20	3	6	2	5	2	17	1	17	5	3	12	4	18	7	15	17	18	18	16	20	18	3	2	13	20
Low maternal BMI	1	2	1	10	2	11	1	2	1	7	1	1	4	1	12	2	4	3	1	2	2	20	3	3	1	13	7	9	2	1	1	1	3	12	3
Poor dietary diversity	18	16	20	4	17	5	18	11	4	2	20	4	20	4	7	7	6	15	19	4	1	19	7	17	20	19	1	8	14	16	6	10	15	8	5
No SBA	8	14	15	6	16	2	7	8	6	3	12	16	8	8	17	5	7	7	11	18	7	5	12	16	14	11	10	20	7	18	5	16	7	3	15
Less than four ANC visits	20	20	18	20	20	20	4	1	2	1	13	20	5	18	16	12	18	11	2	1	6	15	19	11	19	3	2	10	3	3	19	4	20	19	11
Unimproved sanitation	14	11	16	14	13	17	16	16	5	9	4	7	18	10	5	8	9	10	9	12	11	2	2	8	9	4	11	4	4	9	11	19	8	6	10
Had diarrhea, but not used ORT	5	4	5	5	19	19	2	18	9	4	6	17	6	11	4	9	15	18	7	20	13	13	13	18	5	20	17	12	5	2	20	20	11	18	7
Unsafe stool disposal	17	1	14	17	4	6	6	10	16	11	17	10	3	13	3	19	12	6	12	9	4	8	8	4	10	8	13	19	8	11	10	13	4	16	9
High indoor pollution	4	19	13	19	8	8	5	4	11	14	15	9	10	6	19	1	11	9	4	19	10	9	4	2	6	16	14	7	12	4	7	9	18	7	2
Not fully vaccinated	7	17	4	8	12	13	10	17	19	18	10	12	9	7	8	4	13	12	10	8	18	11	16	10	4	5	16	17	11	17	4	6	17	15	18
Infectious disease	10	13	9	11	3	3	20	20	18	19	8	14	17	20	18	6	17	4	15	10	14	17	5	20	18	10	12	3	19	13	3	15	13	4	4
Child marriage	9	10	3	16	9	16	15	14	7	16	18	15	15	3	11	14	10	5	17	14	9	3	15	15	13	7	8	15	10	14	16	12	9	10	12
Delayed breastfeeding	15	15	12	2	15	9	14	12	15	17	9	18	16	16	13	13	14	13	6	11	5	7	10	14	7	6	15	1	9	5	14	5	10	17	17
FP need unsatisfied	13	8	11	7	14	15	19	15	8	15	11	13	13	14	10	20	5	14	13	16	17	18	17	9	8	15	5	2	18	12	12	17	16	9	19
No vit A supplement	12	6	10	9	7	12	17	7	14	5	2	8	7	2	9	15	8	19	16	7	20	14	1	5	17	2	9	16	15	19	17	7	12	5	16
No iodized salt	11	18	6	3	6	18	9	13	12	8	19	11	19	15	20	18	19	2	14	13	15	16	11	13	16	1	19	11	20	15	9	18	1	2	14
Unsafe water	6	12	8	15	5	14	11	6	13	13	14	3	14	17	14	3	16	8	18	17	8	10	14	12	3	14	6	13	17	6	8	8	14	11	13
No care seeking for susp pneu	3	5	7	12	10	10	12	3	3	10	16	19	11	12	6	16	3	16	8	15	3	6	9	6	11	12	3	14	13	10	13	14	6	14	6

Label:
Rank 1st   Rank 2nd   Rank 3rd-4th   Rank 5th-7th   Rank 8th-12nd   Rank 13th-20th

© 2020 Li Z et al. *JAMA Network Open.*

eFigure 12. Country-Specific Odds Ratios for 20 Factors Associated With Child Anthropometric Failures From Fully Adjusted Models, Stratified by Children's Age
A) stunting among children 12-23 months old, B) stunting among children 24-59 months old, C) underweight among children 12-23 months old, D) underweight among children 24-59 months old, E) wasting among children 12-23 months old, and F) wasting among children 24-59 months old

A) Stunting, children 12-23 months old, total n=76,862

	BJ	BF	BI	CM	CI	CD	ET	GM	GH	GN	HT	IN	KE	KG	KM	LS	LR	MW	ML	MZ	MM	NA	NP	NE	PE	ST	SL	SZ	TD	TJ	TZ	TG	UG	ZM	ZW
Short maternal stature	11.1	1.5	33.0	1.0	2.3	3.5	4.0	6.1	11.8	1.2	3.8	4.0	3.2	1.6	0.9	3.6	5.2	13.0	1.0	5.0	2.6	1.0	17.8	2.6	10.0	12.7	1.5	3.8	3.5	1.9	4.8	1.0	3.4	3.1	20.1
Lack of maternal education	2.6	3.4	0.8	12.2	1.5	3.6	3.6	0.4	0.8	1.5	1.5	1.9	1.6	0.8	2.1	2.1	0.7	3.7	3.4	9.1	1.3	0.0	1.4	1.9	2.9	1.4	0.9	2.8	0.9	2.8	0.7	0.5	21.3	1.3	52.5
Poorest HH wealth	1.2	1.1	3.7	2.7	0.6	1.8	2.0	0.7	2.1	1.8	7.8	1.2	1.9	1.1	3.2	31.1	6.1	1.2	5.5	1.4	0.3	168.8	6.6	0.6	5.4	6.7	1.1	9.2	0.7	0.7	2.1	1.4	0.8	1.0	3.6
Low maternal BMI	1.3	1.7	2.0	1.8	3.3	1.6	1.9	2.6	0.2	1.5	1.4	1.4	1.0	5.3	3.4	21.2	0.8	7.1	2.1	0.9	2.3	2.5	0.5	3.3	1.6	2.8	0.8	1.0	1.1	1.4	1.7	3.5	1.4	2.1	2.7
Poor dietary diversity	0.9	1.7	1.4	1.1	0.6	0.8	1.6	1.1	0.8	1.1	2.2	1.0	0.7	2.0	0.7	2.6	0.6	0.8	1.5	1.1	0.5	1.1	1.7	2.2	1.5	10.1	0.5	0.3	1.3	1.6	2.4	1.3	0.6	1.1	0.4
No SBA	1.2	1.2	1.6	1.3	0.6	1.1	1.3	0.9	2.0	0.9	1.2	1.1	1.2	1.0	0.6	1.9	1.0	1.4	1.1	1.4	1.6	0.3	1.0	0.8	1.8	0.9	1.3	0.9	1.2	0.7	0.9	1.2	0.9	2.1	1.1
Less than four ANC visits	4.1	0.4	0.0	1.8	3.3	1.9	0.4	1.6	3.5	8.3	0.8	1.2	1.2	0.4	1.3	4.0	1.1	0.2	0.8	1.3	1.8	81.1	1.3	1.2	1.4	0.5	1.1	0.3	0.2	1.3	2.0	6.5	5.0	1.2	2.5
Unimproved sanitation	1.1	1.2	0.9	0.8	0.8	1.0	1.7	1.6	0.5	1.4	1.2	1.2	1.1	1.1	1.3	0.4	1.0	1.0	0.7	1.1	0.7	0.8	1.1	0.8	0.8	0.9	1.3	0.8	1.1	1.3	1.0	0.8	1.2	1.2	1.3
Had diarrhea, but not used ORT	1.2	0.5	1.1	1.1	2.0	0.8	1.1	1.0	0.5	0.6	0.9	1.0	1.2	1.0	1.5	7.5	0.8	2.5	0.8	1.2	1.0	14.9	0.6	0.9	1.2	3.5	0.3	1.0	1.3	2.5	1.6	0.6	0.9	0.8	0.6
Unsafe stool disposal	1.0	1.2	1.6	1.3	1.5	0.9	0.8	1.3	1.3	0.8	1.2	1.0	1.0	0.7	0.9	0.5	1.0	1.3	1.1	0.9	0.8	57.3	1.7	1.2	0.7	1.0	1.2	0.8	1.1	2.1	1.0	1.2	1.4	1.1	0.9
High indoor pollution	1.7	0.8	1.0	2.2	1.9	0.7	0.4	0.6	1.5	0.6	0.7	1.1	1.2	1.2	0.6	0.4	1.0	0.4	1.2	2.7	1.7	0.4	0.8	0.6	0.8	0.5	1.0	0.2	1.2	0.7	1.1	1.0	0.2	1.8	1.0
Not fully vaccinated	1.1	1.0	0.9	1.1	0.7	0.9	0.9	0.8	1.2	0.8	1.0	1.0	1.1	1.0	2.1	0.9	1.3	0.9	0.9	0.9	0.9	11.8	0.8	1.0	0.9	3.6	1.2	2.1	1.1	0.8	1.0	1.3	0.8	0.9	0.9
Infectious disease	1.2	1.7	1.1	1.0	0.5	1.1	1.1	1.1	0.7	1.4	1.0	1.0	1.0	0.2	1.3	0.6	0.7	0.7	1.4	1.1	0.8	3.5	1.3	1.2	0.6	1.5	1.6	0.6	0.7	0.3	0.8	0.7	1.1	1.1	1.8
Child marriage	1.1	0.9	1.1	0.6	1.1	1.1	0.6	0.7	0.8	0.8	0.4	1.1	1.0	0.8	1.1	0.8	1.1	0.8	0.8	1.0	1.3	0.3	0.9	1.2	0.9	1.3	0.7	1.6	1.0	0.7	0.7	0.9	0.8	1.0	1.0
Delayed breastfeeding	1.0	0.8	1.0	0.9	1.0	1.4	1.2	1.0	1.0	1.0	1.7	1.0	1.1	1.1	1.0	1.5	1.2	1.1	1.0	1.1	1.6	0.9	0.7	1.1	0.8	0.8	1.8	1.0	0.8	1.1	0.9	1.0	1.2	1.1	1.1
FP need unsatisfied	1.2	1.1	1.1	0.9	0.9	0.8	1.2	1.3	0.7	0.9	1.5	1.0	0.9	1.3	1.3	0.2	1.1	1.0	1.0	0.9	1.0	0.5	1.6	1.1	1.5	0.6	1.1	0.8	1.0	1.0	1.0	1.6	0.8	0.8	1.0
No vit A supplement	0.8	1.0	1.0	1.0	1.9	1.5	0.7	3.7	1.0	1.2	0.8	1.0	0.8	0.8	1.3	0.3	1.0	1.1	1.0	0.9	2.0	0.1	1.2	1.5	1.5	6.4	1.6	16.2	1.1	0.7	0.7	1.4	0.9	0.9	1.0
No iodized salt	1.0	0.8	0.8	1.5	1.8	0.6	1.1	0.6	1.8	0.9	1.0	0.9	1.0	0.4	0.7	1.1	0.9	1.1	1.1	1.0	1.1	0.7	1.3	0.7	1.0	0.6	0.9	0.4	0.9	0.6	1.2	1.1	0.9	0.7	0.9
Unsafe water	1.0	1.0	1.0	1.2	1.1	0.9	1.0	0.7	0.8	1.1	1.0	0.9	1.2	1.0	0.4	1.2	1.0	0.6	1.0	1.3	1.3	0.6	1.3	1.1	0.8	0.9	1.2	1.1	1.0	1.1	1.0	1.5	1.3	0.9	0.6
No care seeking for susp pneu	0.8	1.1	0.9	1.0	1.1	1.0	1.0	0.6	1.8	1.0	1.1	0.9	1.0	1.2	0.7	0.7	1.3	1.1	1.7	0.8	0.9	1.8	0.5	0.6	1.0	1.0	0.5	1.2	1.0	1.0	1.0	2.4	0.7	1.0	0.9

Label: odds ratios

>=4.0 | [2.0, 4.0) | [1.3, 2.0) | [1.1, 1.3) | [1.0, 1.1) | <1.0

Note:

1. BJ= Benin, BF= Burkina Faso, BI= Burundi, CM=Cameroon, CI=Côte d'Ivoire, CD=The Democratic Republic of the Congo, ET=Ethiopia, GM=Gambia, GH=Ghana, GN=Guinea, HT=Haiti, IN=India, KE=Kenya, KG=Kyrgyzstan, KM=Comoros, LS=Lesotho, LR=Liberia, MW=Malawi, ML=Mali, MZ=Mozambique, MM=Myanmar, NA=Namibia, NP=Nepal, NE=Niger, PE=Peru, ST= São Tomé and Príncipe, SL=Sierra Leone, SZ=Swaziland, TD=Chad, TJ=Tajikistan, TZ=Tanzania, TG=Togo, UG=Uganda, ZM=Zambia, ZW=Zimbabwe.
2. Short maternal statue: maternal height <145cm; low maternal BMI: maternal BMI <18.5 kg/m2; child marriage: mother's age at marriage <18 years old; delayed breastfeeding: child was not initially breastfed within one hour after born; infectious disease: child was caught by infectious diseases two weeks prior to the survey.
3. Abbreviations - HH: household, BMI: body mass index; SBA: skilled birth attendant; ANC: antenatal care; ORT: oral rehydration therapy; FP: family planning; vit: vitamin; susp pneu: suspected pneumonia.

© 2020 Li Z et al. *JAMA Network Open.*

© 2020 Li Z et al. *JAMA Network Open.*

B) Stunting, children 24-59 months old, total n=222,491

	BJ	BF	BI	CM	CI	CD	ET	GM	GH	GN	HT	IN	KE	KG	KM	LS	LR
Short maternal stature	7.6	17.7	7.6	9.2	3.3	3.4	2.6	0.7	3.7	0.7	30.5	4.6	4.3	4.4	1.3	2.0	4.5
Lack of maternal education	1.9	4.4	5.9	2.1	7.5	1.1	1.7	1.2	12.6	1.2	1.5	2.0	1.1	0.8	2.6	9.8	2.0
Poorest HH wealth	2.9	2.7	2.3	5.0	1.7	3.0	2.3	2.1	1.9	1.8	3.4	1.6	3.1	1.2	1.3	25.7	2.2
Low maternal BMI	1.9	2.0	2.2	1.9	2.6	1.6	1.6	0.7	2.2	0.7	2.0	1.7	1.5	2.3	1.7	2.5	2.9
Poor dietary diversity	1.0	1.3	1.0	2.3	0.8	1.5	2.5	1.3	1.0	3.9	0.7	1.3	1.3	1.1	0.6	0.5	1.4
No SBA	1.4	1.4	1.1	1.0	0.9	0.9	0.9	1.3	1.8	1.4	1.1	1.1	1.4	0.3	1.2	1.4	0.9
Less than four ANC visits	2.1	0.9	1.0	1.4	0.8	0.4	1.3	1.0	0.8	1.0	1.7	1.2	1.2	0.9	1.1	1.6	1.0
Unimproved sanitation	1.0	0.9	1.2	0.9	1.5	0.9	1.2	1.3	0.9	1.0	1.2	1.1	1.2	1.3	0.9	0.7	1.1
Had diarrhea, but not used ORT	1.3	1.8	0.9	1.1	0.6	1.2	1.0	0.6	0.5	2.3	1.2	1.2	1.0	0.9	1.6	7.1	1.2
Unsafe stool disposal	1.1	1.1	1.2	1.2	1.3	1.2	0.7	1.2	1.4	1.3	1.1	1.1	0.9	1.3	0.9	1.1	0.9
High indoor pollution	1.4	0.9	1.0	1.2	1.6	6.9	1.1	0.5	0.7	0.6	1.7	1.1	0.9	1.3	0.7	0.5	1.0
Not fully vaccinated	0.9	1.2	1.8	1.0	1.6	0.9	1.0	1.5	1.7	1.2	1.1	1.0	1.0	1.2	1.2	0.7	0.9
Infectious disease	1.0	0.8	1.1	1.2	1.4	0.9	1.2	0.9	1.4	1.0	0.9	1.1	1.0	2.0	0.8	0.4	1.3
Child marriage	1.0	1.0	1.1	1.0	1.1	1.2	1.1	1.2	1.2	0.9	1.1	1.1	1.1	1.1	1.0	1.0	0.8
Delayed breastfeeding	1.0	1.0	1.1	1.0	0.9	1.1	1.1	1.0	0.8	0.8	1.0	1.1	1.1	1.5	1.0	1.1	1.3
FP need unsatisfied	1.2	1.0	0.9	1.2	1.0	1.1	1.1	1.2	0.8	1.0	1.0	1.0	0.9	1.3	1.3	0.9	1.1
No vit A supplement	1.0	0.8	1.3	1.2	1.3	1.3	0.9	1.0	1.0	1.1	1.0	1.0	0.9	1.1	0.9	0.8	1.2
No iodized salt	0.9	0.8	0.9	1.4	1.5	1.1	0.9	1.0	1.0	1.1	1.1	1.0	1.0	0.9	1.8	1.5	0.5
Unsafe water	0.9	0.9	0.9	1.2	0.8	1.1	1.0	0.8	0.6	1.0	0.9	0.9	1.0	1.3	1.3	0.7	0.9
No care seeking for susp pneu	1.0	1.3	1.0	1.0	1.1	1.5	1.0	0.9	0.4	1.0	1.2	1.0	1.0	0.5	1.4	2.3	0.7

	MW	ML	MZ	MM	NA	NP	NE	PE	ST	SL	SZ	TD	TJ	TZ	TG	UG	ZM	ZW
Short maternal stature	6.5	2.9	4.5	6.5	2.3	9.0	1.5	9.3	2.6	3.6	14.1	1.2	5.5	6.6	11.8	15.0	6.2	4.4
Lack of maternal education	4.7	3.9	1.8	1.2	1.0	1.4	4.0	3.6	0.8	15.9	4.0	4.0	1.0	4.7	1.2	2.9	2.0	3.3
Poorest HH wealth	2.1	3.0	3.1	1.9	4.0	3.0	1.1	8.6	12.3	1.8	1.8	1.1	1.3	2.1	3.2	3.3	2.1	1.5
Low maternal BMI	2.0	2.1	2.3	1.7	3.0	2.0	1.3	2.4	1.4	1.8	6.5	1.6	1.6	1.9	2.5	1.5	2.0	1.8
Poor dietary diversity	1.0	2.5	1.6	1.2	0.6	0.5	1.3	1.1	1.6	1.7	0.4	1.3	1.6	1.4	1.3	2.3	0.9	1.1
No SBA	1.1	1.2	1.0	1.5	1.6	1.0	1.1	1.5	1.1	0.9	2.7	1.2	1.3	1.1	1.0	1.1	0.9	1.3
Less than four ANC visits	0.6	2.2	1.8	1.2	0.7	0.7	5.0	0.7	0.5	1.1	1.8	2.3	1.1	0.5	0.7	2.4	0.8	1.0
Unimproved sanitation	1.0	1.0	1.1	1.2	1.6	1.8	1.0	1.3	0.4	0.9	0.8	0.8	1.0	1.2	0.9	0.8	1.0	1.2
Had diarrhea, but not used ORT	0.9	1.5	1.1	0.9	2.9	0.6	0.8	0.9	0.4	0.5	0.8	1.3	1.5	1.3	1.0	0.8	1.2	0.7
Unsafe stool disposal	0.9	1.4	0.8	1.3	1.3	1.2	1.4	1.2	2.5	0.8	1.0	1.2	1.0	1.0	1.6	1.1	0.9	1.1
High indoor pollution	2.3	0.3	1.9	0.8	2.5	1.1	2.0	1.0	1.0	0.4	0.9	1.5	1.2	1.5	1.9	1.0	1.2	1.8
Not fully vaccinated	1.1	1.1	0.9	1.1	1.4	0.8	1.1	1.0	1.6	1.0	1.7	1.0	0.6	1.0	1.4	0.8	1.0	1.4
Infectious disease	0.9	0.8	1.3	1.1	1.3	1.1	1.4	1.1	1.8	0.7	0.8	1.2	1.0	0.9	1.2	0.7	1.0	1.2
Child marriage	1.0	1.1	1.0	1.1	0.7	0.9	1.0	1.0	1.4	1.1	1.2	0.9	0.8	0.9	1.2	0.8	1.2	1.2
Delayed breastfeeding	1.5	1.0	0.8	0.9	1.2	1.0	1.0	1.0	1.2	1.1	1.2	0.9	0.8	0.9	1.1	0.9	1.0	1.0
FP need unsatisfied	1.3	1.2	1.0	1.1	1.3	0.8	0.9	0.9	0.6	1.2	1.6	1.1	1.0	0.9	0.9	1.0	1.1	1.0
No vit A supplement	0.9	1.4	0.8	1.0	2.4	1.0	1.4	1.3	0.6	1.0	1.4	0.9	0.5	1.2	1.1	0.8	0.9	1.3
No iodized salt	0.8	1.1	0.9	1.0	0.6	0.5	1.0	0.9	0.7	1.7	2.3	1.1	0.9	1.1	0.8	1.0	0.9	0.8
Unsafe water	1.2	1.2	1.0	1.3	0.9	1.3	0.9	0.8	1.1	1.0	1.2	0.9	1.0	1.1	0.9	0.9	1.1	1.1
No care seeking for susp pneu	1.1	0.9	0.8	1.0	1.3	1.0	0.7	1.0	1.0	1.1	1.0	1.0	1.0	1.0	0.7	0.6	1.0	0.9

Label: odds ratios
>=4.0 | [2.0, 4.0) | [1.3, 2.0) | [1.1, 1.3) | [1.0, 1.1) | <1.0

© 2020 Li Z et al. *JAMA Network Open.*

C) Underweight, children 12-23 months old, total n=76,862

	BJ	BF	BI	CM	CI	CD	ET	GM	GH	GN	HT	IN	KE	KG	KM	LS	LR	MW	ML	MZ	MM	NA	NP	NE	PE	ST	SL	SZ	TD	TJ	TZ	TG	UG	ZM	ZW
Short maternal stature	4.3	2.5	5.6	2.1	1.0	1.8	8.3	1.0	5.6	4.5	17.2	3.6	7.6	1.0	2.6	1.5	26.9	8.1	0.0	7.3	5.7	1.0	3.7	0.3	2.5	1.0	1.8	0.3	0.0	1.0	5.0	9.6	3.8	6.3	1.0
Lack of maternal education	1.3	1.6	0.2	2.3	2.8	11.0	4.9	0.5	1.0	2.0	0.5	2.0	2.5	1.5	1.0	1.0	3.4	1.1	8.0	3.4	1.7	18.6	1.1	1.2	1.1	3.8	0.0	17.5	1.3	3.1	1.0	1.4	3.9	1.9	2.9
Poorest HH wealth	0.7	0.8	3.1	0.8	0.8	0.8	3.2	0.6	3.2	2.3	9.8	1.6	2.7	1.5	5.1	1.5	1.5	1.0	1.7	1.1	0.6	1.0	1.2	0.8	1.0	5.6	2.5	50.0	1.0	1.0	1.5	8.0	2.9	1.5	2.5
Low maternal BMI	6.0	4.2	7.6	4.6	6.4	3.1	2.0	2.5	6.3	4.1	11.3	2.6	1.6	2.1	5.3	1.0	8.7	12.6	3.5	2.6	3.8	0.5	1.9	3.8	14.4	34.3	1.2	1.0	1.9	4.8	2.0	3.0	6.5	4.4	4.5
Poor dietary diversity	1.3	1.2	1.7	3.1	1.3	1.9	2.7	5.2	1.4	2.2	1.8	1.5	0.8	8.0	4.9	1.0	3.3	0.8	2.3	1.8	0.4	0.5	3.5	1.8	2.2	1.0	1.3	1.8	1.3	4.4	1.4	1.1	2.6	1.0	0.3
No SBA	1.3	1.8	1.9	1.7	0.9	1.5	1.1	1.3	1.6	1.2	0.4	1.2	1.4	1.0	0.5	1.6	1.3	2.0	1.1	0.9	1.6	0.4	1.0	1.1	1.2	1.5	1.5	0.2	1.2	1.2	0.8	2.1	0.9	1.3	1.7
Less than four ANC visits	0.8	0.1	0.8	1.5	1.3	1.0	1.1	2.6	1.0	1.4	1.2	1.1	2.5	0.6	1.4	0.7	1.1	0.2	0.6	1.2	2.6	20.9	4.2	0.7	0.7	1.0	1.2	0.0	0.7	1.5	1.0	3.3	2.0	3.6	3.0
Unimproved sanitation	1.0	1.5	1.2	1.3	1.1	1.1	2.3	1.7	0.6	0.9	1.6	1.3	0.8	21.2	1.7	0.2	1.4	0.8	1.1	0.9	0.6	0.0	1.6	1.1	1.4	0.6	1.5	0.1	0.8	1.5	0.9	0.7	0.6	1.3	1.2
Had diarrhea, but not used ORT	1.2	0.8	1.7	1.0	1.8	1.0	2.4	1.4	1.3	1.3	0.5	1.1	1.7	1.0	1.7	11.5	1.5	6.7	0.6	2.2	0.6	5.9	0.7	1.8	1.2	3.8	2.3	1.0	1.3	5.2	0.7	0.7	1.3	0.8	0.6
Unsafe stool disposal	0.9	1.2	1.4	1.0	1.6	0.8	1.3	2.2	1.6	0.9	1.7	1.1	1.2	1.5	0.7	2.6	0.9	0.6	1.2	1.2	0.8	1.5	0.9	1.7	0.8	0.4	1.1	6.6	1.4	2.3	1.4	0.9	1.4	1.1	0.8
High indoor pollution	2.3	2.2	0.3	4.7	3.7	1.0	1.1	0.1	1.0	6.1	1.0	1.1	2.5	2.2	0.7	0.3	1.0	1.0	0.3	1.4	2.2	0.7	2.4	0.9	0.8	0.3	1.0	0.0	1.1	0.9	2.1	2.0	1.0	3.5	0.7
Not fully vaccinated	1.2	0.8	1.0	1.8	1.0	0.9	0.8	0.4	0.8	1.0	1.3	1.0	1.4	0.3	2.9	0.2	1.0	1.5	1.1	1.2	1.1	3.7	1.0	1.1	1.2	1.0	1.4	12.5	1.5	0.8	1.2	1.2	0.8	1.0	1.0
Infectious disease	1.2	1.4	0.8	1.1	0.7	0.7	1.0	0.9	1.0	1.2	1.3	1.0	0.9	0.0	1.4	0.1	1.5	0.6	2.4	1.2	1.4	0.1	0.8	1.2	1.2	0.3	1.7	0.0	1.1	0.2	1.0	1.7	0.6	1.0	1.0
Child marriage	1.1	1.0	1.0	0.9	0.6	1.2	0.8	0.8	0.5	1.5	0.4	1.0	0.7	1.0	0.5	1.5	1.3	1.2	1.0	1.1	1.2	0.0	1.0	1.5	0.9	1.0	0.7	1.9	1.0	1.5	0.6	1.2	0.5	1.1	1.3
Delayed breastfeeding	1.0	0.8	1.3	1.6	1.0	1.0	0.9	0.6	0.5	1.1	1.6	1.0	0.8	1.1	1.5	5.7	2.7	0.5	0.9	0.9	1.3	21.4	0.9	0.9	1.3	2.5	1.7	5.4	0.8	1.4	1.1	0.7	1.2	1.4	1.2
FP need unsatisfied	1.3	1.1	0.8	1.1	0.8	1.1	0.9	0.9	0.6	0.6	2.4	0.9	0.8	1.1	3.0	0.3	1.4	0.7	0.9	0.9	0.7	0.3	1.0	1.0	0.8	2.2	0.8	23.5	0.8	0.7	1.2	1.6	0.7	0.8	1.2
No vit A supplement	1.0	0.9	1.2	1.3	1.1	1.5	0.6	7.4	0.5	1.2	0.7	1.1	0.8	1.7	1.2	0.4	0.9	1.1	1.0	1.5	1.0	0.1	0.9	1.3	1.4	20.9	0.6	1.0	0.9	1.2	0.8	0.8	1.1	1.1	0.8
No iodized salt	0.9	1.0	2.7	0.7	0.4	2.3	1.1	0.5	1.4	1.2	2.1	0.9	0.8	1.9	0.6	2.6	0.9	0.6	1.3	0.9	0.8	2.2	1.8	0.7	0.5	0.9	1.3	26.2	1.0	1.2	1.3	1.1	0.8	0.8	3.0
Unsafe water	1.0	1.1	1.3	1.3	0.9	1.4	0.7	1.2	1.4	1.1	0.8	1.0	1.0	1.0	0.5	0.7	0.9	0.9	0.9	1.4	1.3	0.3	1.0	0.9	0.6	1.2	1.7	1.1	1.0	0.9	0.9	0.7	1.7	0.9	0.9
No care seeking for susp pneu	0.9	0.9	1.2	1.0	2.0	1.2	1.0	1.0	3.0	1.0	0.9	0.9	1.0	1.0	0.2	2.4	1.3	1.0	1.7	0.9	0.4	1.0	0.8	0.8	1.0	1.0	1.2	6.1	1.0	1.0	1.0	0.7	0.4	1.0	1.1

Label: odds ratios

>=4.0 [2.0, 4.0) [1.3, 2.0) [1.1, 1.3) [1.0, 1.1) <1.0

© 2020 Li Z et al. *JAMA Network Open.*

D) Underweight, children 24-59 months old, total n=222,491

	BJ	BF	BI	CM	CI	CD	ET	GM	GH	GN	HT	IN	KE	KG	KM	LS	LR	MW	ML	MZ	MM	NA	NP	NE	PE	ST	SL	SZ	TD	TJ	TZ	TG	UG	ZM	ZW
Short maternal stature	4.2	7.6	3.4	3.4	3.9	4.0	3.3	3.3	1.8	1.2	6.7	3.4	3.6	1.0	1.5	7.6	4.6	6.4	2.7	3.4	4.4	1.0	2.2	3.1	19.3	6.5	3.0	12.0	1.3	2.5	6.5	5.0	7.1	4.0	5.1
Lack of maternal education	4.4	1.8	1.7	2.4	7.2	0.7	2.3	1.1	6.2	1.7	2.2	1.7	1.9	1.0	3.5	4.6	0.6	0.7	2.5	2.3	0.6	1.0	1.2	1.5	3.9	0.5	1.8	4.3	1.6	0.5	1.4	1.3	8.9	1.3	2.9
Poorest HH wealth	1.7	1.5	2.8	5.0	1.4	4.0	1.7	1.4	0.6	1.1	1.0	1.6	2.6	0.7	1.3	5.1	1.7	2.3	1.5	3.1	1.0	5.0	1.4	1.4	5.5	13.3	0.9	5.8	1.3	1.1	2.2	0.8	2.8	2.0	0.5
Low maternal BMI	3.0	3.4	6.7	6.8	9.2	2.4	2.8	1.8	3.4	2.6	3.9	2.6	4.1	7.0	3.3	8.2	6.0	2.6	2.3	7.8	3.7	1.7	4.5	3.2	5.2	3.8	2.5	3.4	3.1	2.8	4.1	6.4	3.2	2.3	7.5
Poor dietary diversity	1.0	1.0	1.0	3.0	0.9	1.2	1.7	0.6	1.1	2.4	0.7	1.3	0.9	1.0	0.6	0.4	0.8	1.8	1.4	1.6	3.3	1.1	0.8	1.0	0.5	1.1	1.0	0.4	1.2	0.9	1.1	1.2	2.1	0.8	0.3
No SBA	1.7	1.3	1.4	1.2	0.7	1.1	1.0	1.3	1.6	2.2	1.4	1.1	1.3	1.0	1.3	1.1	1.1	1.2	1.3	0.7	1.6	1.7	1.0	1.0	1.0	1.2	0.9	2.5	1.4	1.1	1.0	1.1	1.4	1.2	1.0
Less than four ANC visits	1.5	1.3	0.8	2.9	0.2	0.3	3.3	1.8	1.0	0.8	1.6	1.1	1.0	0.9	1.0	0.7	0.6	0.9	1.8	0.8	1.5	0.3	0.5	1.0	1.0	1.2	1.6	0.3	1.9	1.4	0.7	0.9	0.8	1.4	0.7
Unimproved sanitation	0.9	1.1	1.1	0.9	1.3	1.0	1.1	1.0	1.3	1.0	1.5	1.1	1.1	1.5	0.7	0.7	1.2	0.7	1.1	1.1	1.2	0.9	2.0	1.0	1.3	1.0	1.5	0.9	1.2	1.1	1.0	1.0	0.8	1.1	1.4
Had diarrhea, but not used ORT	1.2	1.4	1.3	1.7	0.4	1.3	1.5	1.3	0.7	2.2	1.7	1.2	1.1	1.0	1.4	1.1	1.5	0.4	2.0	1.2	0.4	6.1	1.2	0.7	1.3	1.2	1.1	1.0	1.5	1.4	1.7	1.5	0.9	1.0	0.5
Unsafe stool disposal	1.0	1.1	1.2	1.3	1.0	1.3	1.0	0.8	1.3	1.3	1.0	1.1	1.0	1.4	1.5	1.2	1.2	1.8	1.3	1.1	1.6	3.2	0.9	1.5	1.1	2.2	0.7	0.8	1.1	0.6	1.3	1.3	1.6	0.8	1.1
High indoor pollution	1.0	1.3	0.3	0.6	1.3	1.0	0.6	4.2	1.5	1.8	6.0	1.0	1.6	1.4	0.7	0.7	0.3	1.0	2.0	1.4	0.9	9.1	1.3	1.4	1.1	0.5	1.0	0.3	1.2	0.9	2.3	6.7	1.0	1.1	2.1
Not fully vaccinated	0.9	1.2	2.0	1.2	1.3	1.0	1.3	1.1	1.3	1.0	1.4	1.0	0.9	1.0	1.5	0.7	1.0	1.2	1.1	0.9	1.0	1.1	0.7	1.0	0.8	2.0	1.3	1.4	1.4	0.7	1.2	1.4	1.0	1.3	0.7
Infectious disease	1.3	1.1	0.8	1.1	3.2	1.3	0.9	0.5	1.6	0.9	0.5	1.0	1.0	3.9	0.7	0.8	1.2	1.2	0.7	1.2	1.4	0.2	1.8	1.7	1.3	0.9	0.6	2.2	0.9	1.1	1.2	1.0	0.7	1.4	1.5
Child marriage	0.9	1.0	1.1	1.0	1.0	1.1	1.0	1.0	0.6	0.8	1.0	1.0	0.9	0.9	1.2	0.9	0.8	1.1	1.0	0.9	1.3	0.5	0.9	1.0	1.0	1.5	1.1	0.7	0.8	0.8	1.0	1.3	1.0	1.0	1.3
Delayed breastfeeding	1.1	1.0	1.1	0.9	0.5	0.9	1.0	0.9	0.8	0.8	1.1	1.0	0.9	1.1	1.2	1.0	1.0	1.5	1.1	0.8	0.9	1.3	0.9	1.0	1.0	1.3	1.0	0.6	0.9	0.9	0.9	1.0	1.2	1.1	1.0
FP need unsatisfied	1.1	1.2	1.1	1.2	1.4	0.8	1.1	1.0	0.9	1.4	0.8	1.0	1.1	1.2	1.2	0.9	1.5	1.1	1.0	1.1	0.9	1.2	0.8	1.0	1.0	0.5	1.3	0.6	1.0	1.1	1.4	1.1	1.0	1.1	1.5
No vit A supplement	1.2	0.9	1.3	1.2	1.3	1.1	0.9	1.2	1.2	1.9	1.6	1.0	1.0	1.3	1.0	0.6	0.9	0.7	1.0	0.7	1.0	1.7	1.7	1.3	1.6	1.8	1.3	0.4	0.8	0.5	1.1	1.0	1.2	0.8	0.7
No iodized salt	0.8	1.0	1.5	1.6	4.8	1.6	1.2	0.8	1.2	1.4	1.5	0.9	0.7	0.7	0.7	1.0	0.2	1.3	0.9	0.8	0.8	0.6	0.9	1.0	5.4	1.4	1.2	0.8	0.8	1.2	1.2	0.8	1.2	0.9	1.4
Unsafe water	0.9	1.0	1.0	1.0	0.6	1.1	1.0	1.1	0.8	0.9	0.8	1.0	1.1	0.5	1.4	0.6	0.7	1.2	1.0	1.0	1.2	1.1	1.2	0.9	1.2	1.2	1.4	0.8	0.8	0.7	1.0	1.1	0.9	1.0	1.1
No care seeking for susp pneu	1.1	0.9	1.4	1.0	0.6	1.3	1.0	1.2	0.4	1.0	2.1	0.9	1.0	0.4	1.2	0.3	0.6	0.8	1.3	1.6	0.9	4.0	1.1	0.6	1.0	1.0	1.7	0.4	1.0	1.0	1.0	0.6	0.9	1.0	0.8

Label: odds ratios

>=4.0 [2.0, 4.0) [1.3, 2.0) [1.1, 1.3) [1.0, 1.1) <1.0

© 2020 Li Z et al. *JAMA Network Open.*

E) Wasting, children 12-23 months old, total n=76,862

	BJ	BF	BI	CM	CI	CD	ET	GM	GH	GN	HT	IN	KE	KG	KM	LS	LR	MW	ML	MZ	MM	NA	NP	NE	PE	ST	SL	SZ	TD	TJ	TZ	TG	UG	ZM	ZW
Short maternal stature	1.0	2.4	0.5	2.4	1.0	3.2	3.9	1.0	1.0	1.0	1.0	1.5	1.0	1.0	4.5	1.0	1.0	6.5	1.0	3.7	0.9	1.0	0.6	2.1	1.4	1.0	0.4	1.0	1.3	1.0	1.2	21.8	3.0	1.2	5.7
Lack of maternal education	0.8	2.9	0.0	2.9	7.9	1.1	0.7	1.0	1.0	1.2	1.1	1.1	1.9	6.2	0.7	1.0	0.3	1.0	4.8	1.6	0.5	26.6	1.9	0.6	1.0	8.1	0.8	1.0	1.0	1.6	2.0	1.9	1.3	1.0	1.0
Poorest HH wealth	0.5	0.9	1.2	0.6	1.9	0.6	6.2	2.4	3.3	1.3	1.1	1.4	1.8	0.4	1.7	0.0	0.3	0.8	1.0	2.4	3.0	1.0	0.5	3.3	0.2	0.4	0.5	1.0	0.9	1.3	0.8	3.3	5.3	5.9	32.0
Low maternal BMI	10.4	3.1	1.0	4.2	6.5	4.2	1.7	1.9	3.3	2.8	4.0	2.1	1.2	0.8	1.7	1.0	1.0	2.5	2.1	1.2	1.5	18.2	1.5	3.2	28.3	9.0	4.7	1.0	5.2	2.8	1.3	7.4	1.0	1.3	2.9
Poor dietary diversity	1.5	4.8	1.0	3.5	1.1	1.1	3.0	2.6	0.3	9.1	0.3	1.4	1.8	0.0	14.0	1.0	0.4	2.5	1.4	1.2	1.0	0.2	6.2	0.8	2.6	12.5	1.8	1.0	1.4	1.2	6.1	0.5	0.5	1.1	2.9
No SBA	1.4	1.6	2.6	4.9	0.6	1.4	1.4	0.6	0.9	0.8	1.6	1.1	1.7	1.0	0.9	0.0	2.0	2.9	1.1	0.7	1.4	2.2	1.0	0.8	2.5	0.8	1.1	1.0	0.8	1.4	0.9	2.6	0.8	2.3	0.7
Less than four ANC visits	0.6	0.3	1.0	0.4	0.4	1.1	7.9	19.0	3.3	0.5	1.9	1.0	7.9	0.6	0.8	0.0	1.8	0.3	0.6	0.2	1.0	26.4	2.2	0.4	1.2	10.9	0.8	0.0	1.0	2.8	0.9	0.1	1.2	0.8	4.0
Unimproved sanitation	1.3	0.9	1.3	2.4	0.8	0.8	0.7	1.2	1.1	0.8	0.9	1.2	0.7	18.8	1.5	1.0	0.9	0.3	1.4	0.8	0.8	0.0	0.9	0.9	1.9	1.0	4.0	0.0	1.4	1.0	0.3	1.3	0.5	1.1	1.4
Had diarrhea, but not used ORT	1.3	1.1	2.2	1.0	0.8	1.8	1.2	0.7	43.3	0.8	0.3	1.1	2.6	1.0	1.1	1.0	13.5	6.2	0.8	1.1	0.6	0.4	2.8	1.8	0.4	2.6	3.0	1.0	0.8	2.1	0.5	1.1	0.5	2.9	1.0
Unsafe stool disposal	0.7	1.5	1.0	0.7	0.9	0.4	1.2	1.5	0.5	0.5	1.3	1.0	1.0	0.5	1.1	1.0	0.9	0.4	1.2	1.1	0.8	0.1	1.8	1.2	1.1	0.6	1.7	1.0	1.6	1.5	1.5	0.6	0.3	0.8	0.8
High indoor pollution	2.4	0.7	0.2	1.8	5.0	1.0	10.8	0.1	0.1	6.2	1.0	1.0	1.0	0.4	1.5	1.0	1.0	0.5	2.7	1.8	0.7	7.5	1.3	1.1	7.0	0.8	1.0	0.0	2.3	0.6	1.0	0.4	1.0	0.4	0.5
Not fully vaccinated	1.4	1.1	0.9	1.4	0.5	1.2	0.7	1.1	1.1	1.0	1.8	1.0	1.3	4.0	1.8	1.0	1.5	1.6	0.9	1.1	2.3	0.6	0.9	1.1	2.5	0.4	1.7	0.0	1.5	1.3	1.6	1.1	0.9	1.1	0.6
Infectious disease	1.3	1.5	0.8	0.8	2.4	1.6	1.7	1.5	0.1	2.3	0.1	1.0	0.7	0.0	1.1	0.0	0.3	0.7	2.2	1.4	1.4	0.0	1.4	1.5	6.3	0.1	1.2	1.0	1.7	0.6	2.3	1.5	1.2	0.5	0.6
Child marriage	1.2	0.8	1.0	0.7	1.1	1.1	1.4	0.8	1.2	1.1	0.7	1.0	0.7	1.0	0.3	0.0	0.4	1.0	1.1	0.8	0.9	0.2	0.8	1.2	1.4	1.0	1.7	0.0	0.9	1.7	0.7	2.6	0.5	1.3	1.2
Delayed breastfeeding	1.3	1.2	1.1	1.2	0.5	0.9	0.8	0.7	0.5	0.4	2.0	1.0	1.0	1.4	0.5	0.0	3.3	0.4	1.0	0.9	0.8	1.0	0.8	0.9	1.1	1.1	1.4	1.0	1.3	1.8	1.7	0.8	0.9	1.6	0.8
FP need unsatisfied	1.0	1.1	0.8	0.9	1.2	0.8	0.9	0.8	0.8	0.5	1.5	1.0	0.7	1.3	2.4	1.0	0.9	2.7	0.7	0.8	0.7	0.1	1.6	0.9	0.8	0.7	0.8	1.0	0.9	0.8	1.0	1.2	1.0	1.0	0.3
No vit A supplement	1.5	1.2	0.9	0.6	0.6	2.4	0.8	2.2	5.9	0.7	1.3	1.0	1.2	3.0	0.9	1.0	1.3	2.1	0.7	1.7	0.7	0.1	0.3	1.3	0.1	2.2	0.3	1.0	0.9	1.0	1.2	1.0	0.7	0.9	0.4
No iodized salt	1.7	0.6	2.6	0.4	0.5	1.4	0.8	0.4	0.7	1.0	5.6	1.0	0.6	1.0	0.9	0.0	6.1	5.0	1.1	1.0	0.9	1.0	8.9	0.7	0.1	2.6	0.9	0.0	1.0	2.1	0.7	1.7	0.3	1.4	0.9
Unsafe water	1.1	1.0	1.8	0.7	0.7	0.9	1.1	0.9	1.0	0.8	1.1	1.1	1.2	1.7	1.1	0.0	1.1	1.6	0.7	0.9	1.3	0.0	1.1	1.0	1.7	0.5	1.2	0.0	0.9	0.7	0.7	1.0	3.7	1.0	1.2
No care seeking for susp pneu	1.1	1.0	1.3	1.0	0.7	0.8	1.0	2.0	1.2	1.0	4.4	0.8	1.0	1.0	0.9	1.0	4.6	1.4	1.3	1.2	0.8	22.7	1.5	0.9	1.0	1.0	1.6	1.0	1.0	1.0	1.0	0.9	1.1	1.0	0.7

Label: odds ratios
>=4.0 [2.0, 4.0) [1.3, 2.0) [1.1, 1.3) [1.0, 1.1) <1.0

© 2020 Li Z et al. *JAMA Network Open.*

F) Wasting, children 24-59 months old, total n=222,491

	BJ	BF	BI	CM	CI	CD	ET	GM	GH	GN	HT	IN	KE	KG	KM	LS	LR	MW	ML	MZ	MM	NA	NP	NE	PE	ST	SL	SZ	TD	TJ	TZ	TG	UG	ZM	ZW
Short maternal stature	2.7	2.0	0.4	1.0	1.0	1.2	1.0	1.8	1.0	2.0	1.3	1.1	1.0	1.0	0.8	1.0	0.0	10.3	0.0	2.0	0.7	1.0	0.3	3.8	7.5	0.8	0.2	1.0	6.0	1.0	2.4	1.0	1.5	1.7	5.5
Lack of maternal education	0.7	1.2	0.3	0.5	3.4	1.0	1.7	1.4	0.6	0.9	1.3	1.2	7.3	0.2	2.0	1.0	1.9	0.1	1.5	2.3	0.5	17.6	1.8	0.6	1.0	1.5	1.6	1.0	1.6	1.0	0.8	4.8	0.1	0.5	1.0
Poorest HH wealth	0.5	1.3	3.4	27.1	0.5	3.6	1.2	0.4	0.2	0.5	1.7	1.1	3.8	1.6	1.8	0.3	5.1	0.7	1.4	2.8	0.8	2.2	0.5	1.3	0.7	0.8	0.7	0.2	0.9	0.2	0.7	4.0	6.7	1.1	0.2
Low maternal BMI	3.8	2.5	9.7	1.3	2.4	0.9	4.7	3.6	6.3	1.7	3.3	2.4	1.6	5.3	0.9	31.0	1.7	2.9	3.8	3.7	2.4	0.2	2.8	2.8	22.1	0.8	1.3	1.0	4.3	5.4	3.8	5.2	4.7	1.1	3.8
Poor dietary diversity	0.5	0.7	0.3	2.1	0.5	1.1	0.7	1.2	3.4	2.5	0.0	1.1	0.5	1.8	1.3	1.0	1.2	0.7	0.5	2.7	7.8	0.2	1.7	0.8	0.4	0.7	4.6	1.0	0.9	0.7	1.5	1.0	0.5	1.2	1.5
No SBA	1.1	0.8	1.0	1.6	0.6	2.0	1.2	1.4	2.1	2.4	0.8	0.9	1.1	1.0	0.6	2.1	1.1	1.2	1.0	0.5	1.4	2.0	1.0	0.8	0.7	1.1	1.1	0.1	1.2	0.6	1.6	0.6	1.3	1.4	0.7
Less than four ANC visits	0.4	0.0	0.3	0.4	0.1	0.5	1.4	16.5	5.2	4.6	0.8	0.9	1.3	0.2	0.7	1.0	0.2	1.0	2.3	4.2	1.4	1.0	0.4	1.0	0.4	2.9	1.7	1.0	2.6	2.0	0.5	1.6	0.1	0.6	0.9
Unimproved sanitation	0.9	0.8	0.9	0.8	0.9	0.7	0.9	0.8	2.9	1.2	1.6	1.1	0.7	1.0	1.5	1.0	1.1	1.1	1.2	1.1	0.9	17.5	3.3	1.2	1.1	2.1	1.1	1.0	1.8	1.0	1.2	0.5	1.3	1.3	0.9
Had diarrhea, but not used ORT	2.2	1.6	1.5	1.9	0.3	0.5	2.0	0.5	1.0	2.2	1.3	0.9	1.3	1.0	1.5	1.0	0.6	0.6	1.3	0.3	0.8	1.0	1.0	0.7	1.5	0.4	0.7	1.0	1.7	2.6	0.3	0.4	0.8	0.6	1.0
Unsafe stool disposal	0.6	2.5	1.0	0.7	1.7	1.0	1.3	1.3	0.6	0.9	0.5	1.0	1.8	1.0	1.5	0.1	0.9	1.3	1.0	1.5	1.5	1.4	1.5	1.4	1.0	1.6	1.0	0.1	1.2	1.0	1.3	0.8	2.1	0.8	0.9
High indoor pollution	2.5	0.5	1.0	0.5	1.2	1.0	1.3	3.0	1.0	0.9	0.5	1.0	1.0	1.1	0.5	42.1	1.0	1.1	1.4	0.5	0.9	1.3	2.3	3.0	1.3	0.8	1.0	1.0	1.0	1.3	1.4	1.1	0.2	1.2	4.9
Not fully vaccinated	1.1	0.7	1.7	1.5	1.0	0.9	1.1	0.6	0.2	0.6	0.9	1.0	1.0	1.0	1.3	2.2	0.7	1.0	1.1	1.6	0.6	1.1	0.6	1.1	1.5	2.0	0.7	0.2	1.1	0.6	1.7	1.5	0.4	1.0	0.4
Infectious disease	1.1	0.8	1.2	1.1	2.0	1.2	0.3	0.3	0.5	0.5	1.2	1.0	0.8	0.0	0.6	1.2	0.5	2.6	0.8	1.2	0.7	0.3	2.1	0.6	0.6	1.4	1.1	2.2	0.6	0.9	1.8	0.8	0.7	1.3	2.0
Child marriage	1.1	1.1	1.9	0.8	1.2	0.8	1.0	1.1	1.1	0.7	0.5	1.0	0.9	2.8	1.1	0.5	1.0	1.4	0.8	0.7	1.1	2.3	1.0	0.8	0.9	1.7	1.1	0.2	1.2	0.9	0.8	0.9	1.2	1.1	0.9
Delayed breastfeeding	0.8	0.7	1.2	2.6	0.7	1.0	1.0	1.2	0.8	0.6	1.1	0.9	0.9	0.5	0.9	0.9	0.7	0.9	1.4	1.1	1.5	1.5	1.2	0.9	1.2	1.9	0.8	12.6	1.2	1.3	1.0	1.5	1.1	0.7	0.6
FP need unsatisfied	0.9	1.3	1.2	1.5	0.7	0.8	0.7	1.0	1.1	0.8	0.9	1.0	0.9	0.7	1.2	0.0	1.3	0.8	0.9	0.6	0.6	0.2	0.6	1.1	1.1	0.8	1.4	4.0	0.8	0.9	1.0	0.6	0.5	1.1	0.4
No vit A supplement	1.0	1.4	1.2	1.3	1.3	0.9	0.8	1.5	0.9	2.2	2.2	1.1	1.1	4.2	1.3	0.5	1.1	0.5	0.8	1.6	0.4	1.0	3.4	1.3	0.6	7.3	1.1	0.2	0.9	0.5	0.7	1.4	0.8	1.3	0.6
No iodized salt	1.1	0.7	1.5	2.4	1.3	0.6	1.2	1.1	0.9	1.3	0.4	1.0	0.5	0.6	0.3	0.2	0.0	3.4	0.9	1.0	0.7	0.6	1.1	0.9	0.7	12.0	0.6	1.0	0.6	0.9	1.4	0.6	11.5	1.5	0.8
Unsafe water	1.2	0.8	1.3	0.8	1.5	0.9	1.0	1.7	0.9	0.9	0.7	1.1	0.9	0.3	0.8	3.7	0.6	1.2	0.6	0.6	1.1	1.2	1.0	0.9	2.4	0.8	1.3	0.7	0.8	1.1	1.4	1.2	0.5	1.1	0.8
No care seeking for susp pneu	2.5	1.5	1.5	1.0	1.1	0.9	1.0	3.2	4.2	1.0	0.5	0.9	1.0	1.0	1.4	0.4	1.8	0.7	1.2	0.6	1.6	1.5	1.4	1.3	1.0	1.0	1.6	0.3	1.0	1.0	1.0	0.8	1.4	1.0	1.2

Label: odds ratios

>=4.0 [2.0, 4.0) [1.3, 2.0) [1.1, 1.3) [1.0, 1.1) <1.0

© 2020 Li Z et al. *JAMA Network Open.*

eFigure 13. Relative Ranking of 20 Factors Associated With Child Anthropometric Failures From Fully Adjusted Models for Pooled Sample, Stratified by Place of Residence
A) stunting, B) underweight, and C) wasting, odds ratio (OR) and 95% confidence interval (CI)

A) Stunting

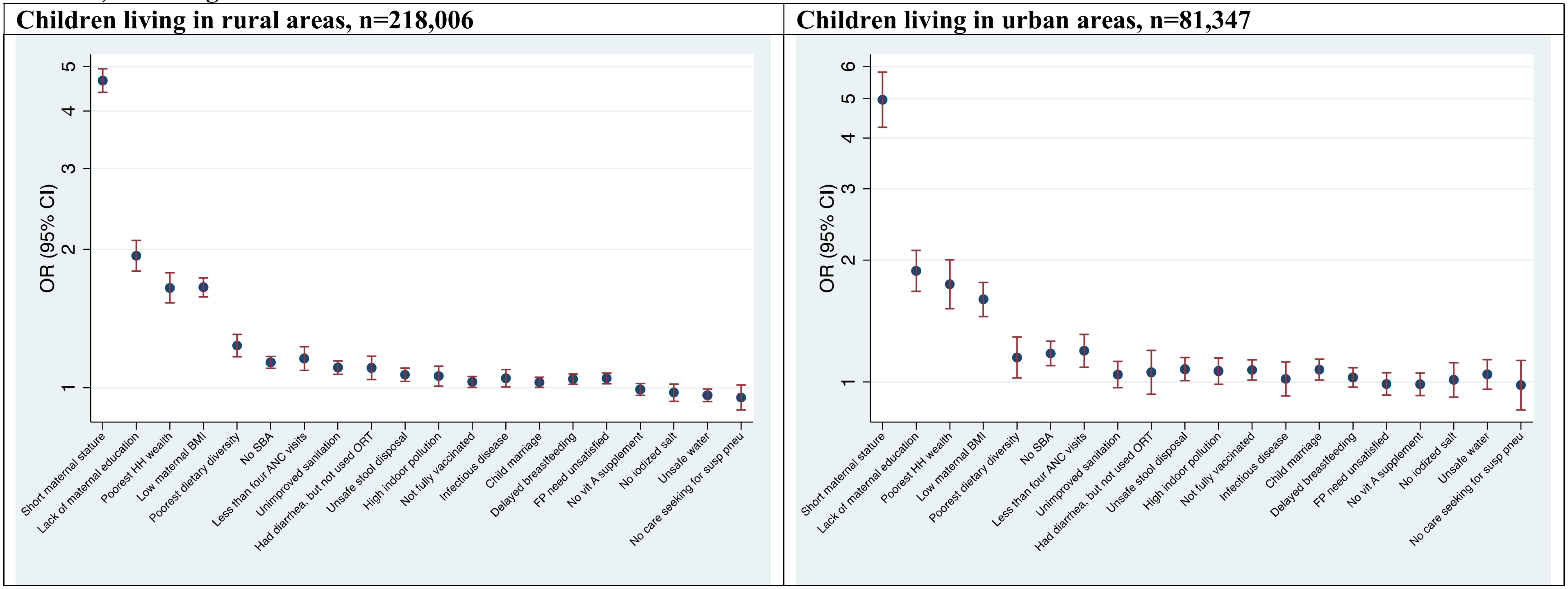

Note:

1. We use logarithmic scale for the y axis.
2. Short maternal statue: maternal height <145cm; low maternal BMI: maternal BMI <18.5 kg/m2; child marriage: mother's age at marriage <18 years old; delayed breastfeeding: child was not initially breastfed within one hour after born; infectious disease: child was caught by infectious diseases two weeks prior to the survey.
3. Abbreviations - HH: household, BMI: body mass index; SBA: skilled birth attendant; ANC: antenatal care; ORT: oral rehydration therapy; FP: family planning; vit: vitamin; susp pneu: suspected pneumonia.

© 2020 Li Z et al. *JAMA Network Open.*

B) Underweight

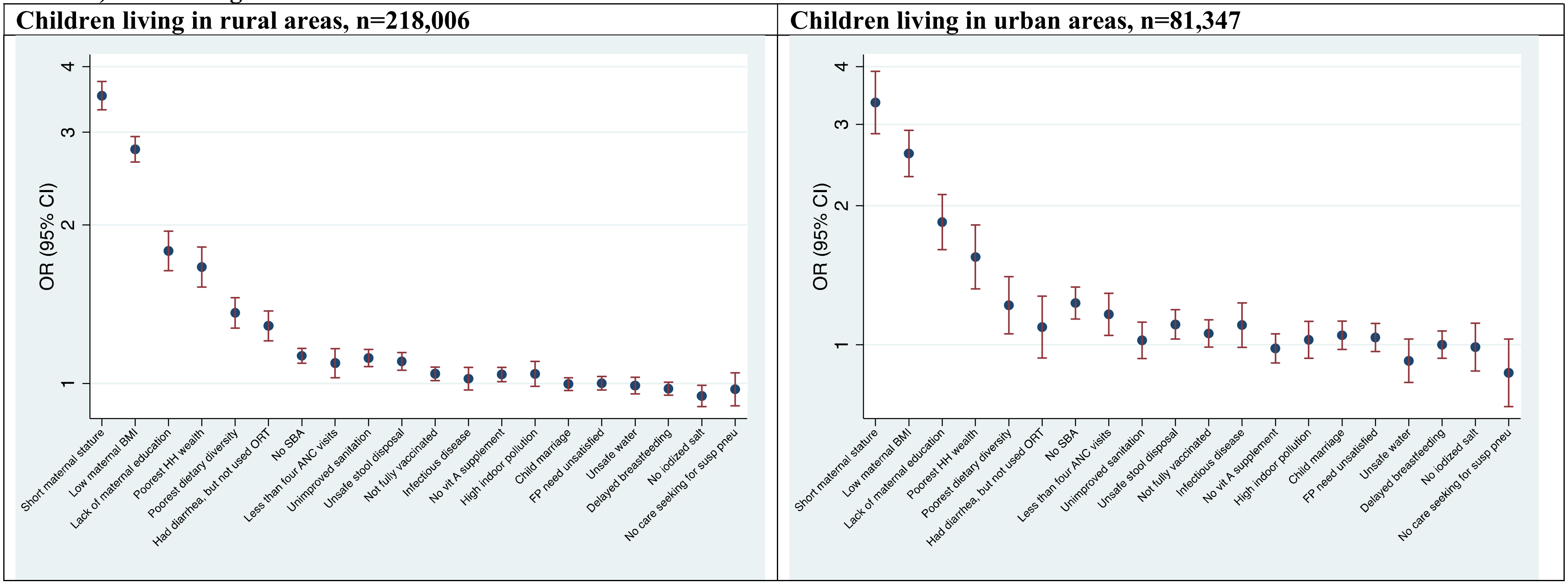

© 2020 Li Z et al. *JAMA Network Open.*

C) Wasting

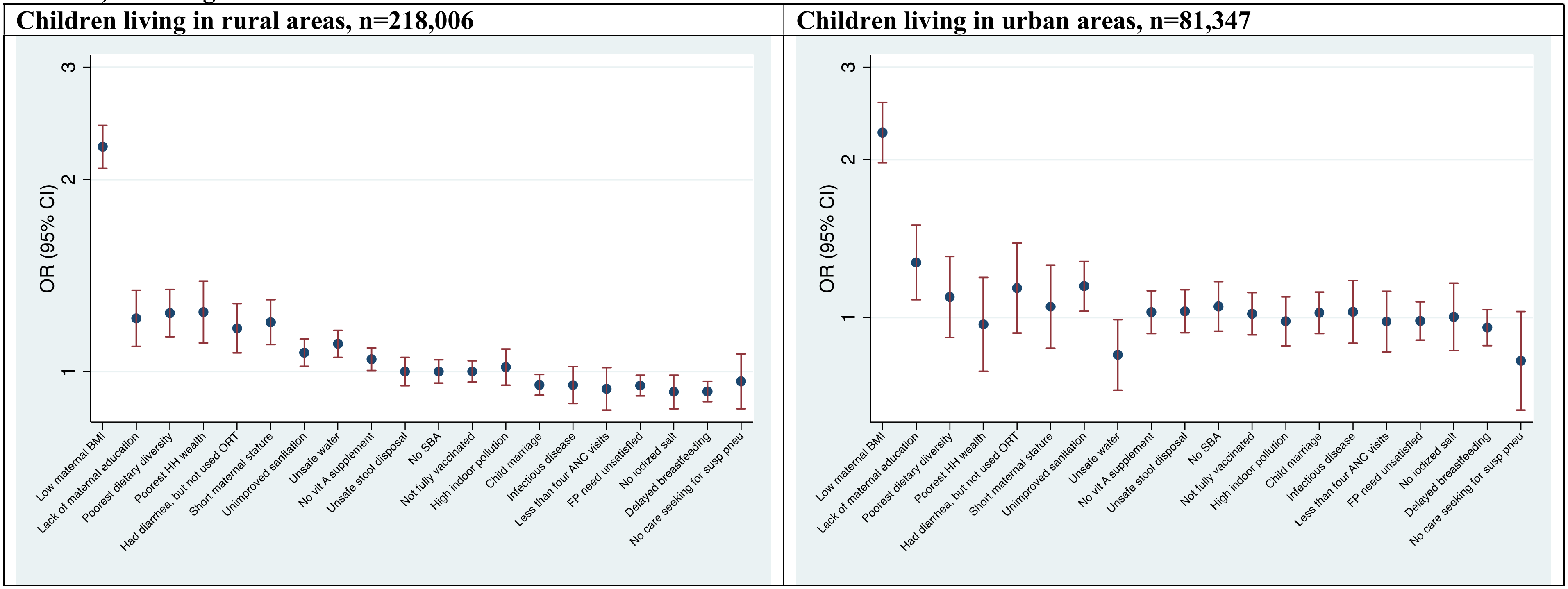

© 2020 Li Z et al. *JAMA Network Open.*

eFigure 14. Country-Specific Ranking of 20 Factors Associated With Child Anthropometric Failures From Fully Adjusted Models, Stratified by Place of Residence
A) stunting among children living in rural areas, B) stunting among children living in urban areas, C) underweight among children living in rural areas, D) underweight among children living in urban areas, E) wasting among children living in rural areas, and F) wasting among children living in urban areas

A) Stunting, children living in rural areas, total n=218,006

	BJ	BF	BI	CM	CI	CD	ET	GM	GH	GN	HT	IN	KE	KG	KM	LS	LR	MW	ML	MZ	MM	NA	NP	NE	PE	ST	SL	SZ	TD	TJ	TZ	TG	UG	ZM	ZW
Short maternal stature	1	1	1	2	5	2	1	3	1	20	1	1	1	1	7	3	2	1	7	1	1	2	1	8	1	1	1	3	8	1	1	2	1	1	1
Lack of maternal education	5	2	3	7	3	1	4	4	2	7	8	2	8	17	1	6	9	2	1	14	5	17	19	5	2	15	7	14	20	7	2	6	3	2	2
Poorest HH wealth	4	3	2	1	4	3	3	12	18	1	2	4	2	12	3	1	1	4	2	3	7	6	2	11	3	2	2	1	18	19	3	4	4	4	20
Low maternal BMI	6	4	4	4	2	4	6	9	5	19	4	3	4	2	2	4	3	3	4	4	2	8	6	1	20	8	4	2	2	3	4	3	6	3	3
Poor dietary diversity	17	5	17	3	6	8	2	20	11	3	9	5	18	11	18	13	4	10	3	5	20	11	18	2	5	3	6	19	3	2	7	9	5	9	17
No SBA	8	6	5	12	19	14	11	10	3	12	12	10	5	3	9	8	10	11	12	9	3	12	14	13	4	13	13	5	4	8	6	17	10	12	10
Less than four ANC visits	2	20	20	6	20	20	19	2	10	2	10	6	7	19	15	5	17	20	5	2	6	4	4	3	19	20	14	16	5	6	20	1	2	5	5
Unimproved sanitation	16	15	9	18	13	16	10	7	14	8	6	7	10	16	16	16	8	14	17	6	16	20	3	10	7	18	16	17	13	14	13	13	14	18	7
Had diarrhea, but not used ORT	7	10	12	9	18	9	5	17	15	14	5	8	16	20	4	2	18	7	8	10	19	7	8	18	16	10	19	4	6	5	5	10	13	19	19
Unsafe stool disposal	13	8	6	8	11	7	18	8	4	10	16	9	17	7	19	9	12	12	6	20	8	14	5	7	17	9	17	15	9	4	8	7	7	14	13
High indoor pollution	3	19	13	20	1	15	20	6	17	5	3	13	3	8	20	19	11	5	20	11	14	1	12	20	8	6	20	20	1	9	19	16	11	6	4
Not fully vaccinated	12	11	7	13	9	19	14	5	6	9	15	16	12	5	6	14	14	18	19	16	17	3	10	14	13	4	12	6	10	17	17	5	17	8	9
Infectious disease	10	9	11	17	15	18	9	16	7	4	14	11	19	9	12	20	6	19	18	7	13	13	11	6	11	5	18	18	7	18	16	18	19	7	6
Child marriage	11	16	10	19	10	11	16	18	9	18	19	12	11	14	10	12	13	16	15	17	9	19	17	12	14	11	11	10	15	16	18	8	15	10	11
Delayed breastfeeding	14	13	15	16	16	12	7	14	13	17	7	14	6	4	14	11	5	6	16	13	15	16	15	9	9	19	5	13	19	13	15	11	16	15	14
FP need unsatisfied	9	12	14	10	14	17	8	11	16	13	13	15	14	6	5	18	7	9	13	15	12	9	13	16	10	17	8	9	11	10	11	14	9	13	8
No vit A supplement	19	18	8	15	7	5	17	1	8	6	20	17	20	13	17	17	15	15	9	18	11	10	7	4	6	7	10	8	17	20	14	12	12	17	16
No iodized salt	18	17	19	5	8	10	15	15	12	11	17	19	13	15	8	10	16	17	14	12	10	18	20	15	15	16	3	12	12	15	10	15	20	20	12
Unsafe water	15	14	18	11	17	13	13	19	19	16	18	20	9	10	13	15	19	13	11	8	4	15	9	17	18	12	9	11	16	12	9	20	8	11	15
No care seeking for susp pneu	20	7	16	14	12	6	12	13	20	15	11	18	15	18	11	7	20	8	10	19	18	5	16	19	12	14	15	7	14	11	12	19	18	16	18

Label:
Rank 1st | Rank 2nd | Rank 3rd-4th | Rank 5th-7th | Rank 8th-12nd | Rank 13th-20th

Note:

1. BJ= Benin, BF= Burkina Faso, BI= Burundi, CM=Cameroon, CI=Côte d'Ivoire, CD=The Democratic Republic of the Congo, ET=Ethiopia, GM=Gambia, GH=Ghana, GN=Guinea, HT=Haiti, IN=India, KE=Kenya, KG=Kyrgyzstan, KM=Comoros, LS=Lesotho, LR=Liberia, MW=Malawi, ML=Mali, MZ=Mozambique, MM=Myanmar, NA=Namibia, NP=Nepal, NE=Niger, PE=Peru, ST= São Tomé and Príncipe, SL=Sierra Leone, SZ=Swaziland, TD=Chad, TJ=Tajikistan, TZ=Tanzania, TG=Togo, UG=Uganda, ZM=Zambia, ZW=Zimbabwe.
2. Short maternal statue: maternal height <145cm; low maternal BMI: maternal BMI <18.5 kg/m2; child marriage: mother's age at marriage <18 years old; delayed breastfeeding: child was not initially breastfed within one hour after born; infectious disease: child was caught by infectious diseases two weeks prior to the survey.
3. Abbreviations - HH: household, BMI: body mass index; SBA: skilled birth attendant; ANC: antenatal care; ORT: oral rehydration therapy; FP: family planning; vit: vitamin; susp pneu: suspected pneumonia.

© 2020 Li Z et al. *JAMA Network Open.*

B) Stunting, children living in urban areas, total n=81,347

	BJ	BF	BI	CM	CI	CD	ET	GM	GH	GN	HT	IN	KE	KG	KM	LS	LR	MW	ML	MZ	MM	NA	NP	NE	PE	ST	SL	SZ	TD	TJ	TZ	TG	UG	ZM	ZW
Short maternal stature	1	14	1	16	2	1	18	8	1	18	15	1	20	2	17	18	2	1	20	1	1	2	1	10	1	8	11	17	1	1	1	14	2	1	2
Lack of maternal education	5	2	2	1	1	4	6	15	2	1	6	2	13	10	1	9	1	7	1	4	20	4	2	1	4	7	1	2	2	19	6	6	3	3	1
Poorest HH wealth	3	4	4	15	15	3	2	4	17	7	1	4	1	20	6	10	6	8	6	2	18	17	3	19	2	1	14	14	6	17	3	3	8	5	13
Low maternal BMI	4	3	6	4	4	6	1	14	11	9	2	3	8	1	2	11	3	4	11	3	3	1	4	4	3	2	2	13	3	7	5	1	5	2	3
Poor dietary diversity	20	5	3	8	20	5	12	5	15	6	17	7	2	4	20	7	10	19	2	6	10	19	18	7	17	14	20	20	7	4	2	2	14	16	4
No SBA	9	9	13	10	13	17	11	6	3	3	12	8	4	13	19	5	17	6	4	10	6	13	11	11	5	12	16	19	10	6	16	7	10	10	6
Less than four ANC visits	2	1	19	3	3	20	8	18	4	8	4	5	7	12	3	4	9	20	10	9	12	20	15	2	7	15	4	8	4	12	12	8	4	20	14
Unimproved sanitation	12	13	12	19	6	18	5	3	18	11	8	18	3	3	5	20	14	14	18	16	5	5	5	16	9	20	5	15	20	8	9	19	11	6	17
Had diarrhea, but not used ORT	14	12	15	13	11	16	20	17	20	2	20	11	5	14	7	1	4	2	5	15	13	3	20	14	12	13	18	16	11	5	13	20	15	4	16
Unsafe stool disposal	16	7	16	18	9	15	15	7	14	10	3	9	17	15	13	16	11	5	17	7	9	11	8	3	8	3	3	18	8	13	17	10	7	15	18
High indoor pollution	6	15	11	6	7	2	7	20	9	19	10	12	14	8	11	19	13	11	19	5	14	6	10	9	16	18	19	7	5	3	8	4	19	7	7
Not fully vaccinated	13	8	14	12	14	13	17	11	5	17	13	10	15	16	4	15	7	3	3	12	4	9	16	6	11	6	8	12	19	20	7	11	13	18	12
Infectious disease	18	16	9	7	16	8	9	16	10	14	16	16	11	6	8	17	18	18	14	13	15	7	7	8	19	4	7	10	18	10	15	5	6	17	11
Child marriage	11	17	10	9	18	7	14	2	6	16	9	14	6	7	9	8	19	12	12	8	2	10	6	5	13	9	15	11	15	14	18	17	16	8	10
Delayed breastfeeding	10	18	7	20	19	10	10	10	19	20	18	13	12	19	12	3	8	17	7	19	8	8	14	18	20	5	13	5	13	16	14	15	9	13	8
FP need unsatisfied	7	10	20	11	12	12	4	1	13	15	7	20	19	9	18	12	16	9	15	14	11	12	13	13	10	16	6	9	9	18	20	12	18	11	20
No vit A supplement	15	11	17	5	10	9	19	19	8	4	14	17	16	11	10	13	5	10	13	20	7	15	12	12	6	11	9	6	17	9	19	13	17	19	9
No iodized salt	17	6	18	17	8	19	3	13	7	12	11	6	18	18	14	6	20	13	16	18	19	14	17	17	15	17	12	1	14	15	4	18	1	9	19
Unsafe water	19	19	5	2	17	14	16	9	12	5	19	15	9	5	15	2	12	15	9	17	17	16	9	15	18	19	10	4	16	2	10	9	12	12	15
No care seeking for susp pneu	8	20	8	14	5	11	13	12	16	13	5	19	10	17	16	14	15	16	8	11	16	18	19	20	14	10	17	3	12	11	11	16	20	14	5

Label:

Rank 1st   Rank 2nd   Rank 3rd-4th   Rank 5th-7th   Rank 8th-12nd   Rank 13th-20th

© 2020 Li Z et al. *JAMA Network Open.*

C) Underweight, children living in rural areas, total n=218,006

	BJ	BF	BI	CM	CI	CD	ET	GM	GH	GN	HT	IN	KE	KG	KM	LS	LR	MW	ML	MZ	MM	NA	NP	NE	PE	ST	SL	SZ	TD	TJ	TZ	TG	UG	ZM	ZW
Short maternal stature	1	1	2	6	4	1	2	1	2	2	1	1	1	14	3	2	2	1	7	2	1	14	2	7	2	4	1	1	10	3	1	1	2	1	2
Lack of maternal education	11	4	19	5	3	3	1	20	5	7	3	3	4	20	6	3	12	20	4	7	19	16	7	3	3	19	6	6	2	18	5	3	1	8	15
Poorest HH wealth	8	3	3	4	6	2	4	13	20	20	16	4	2	4	2	5	18	3	2	3	11	4	5	9	1	5	17	4	15	1	3	19	3	4	20
Low maternal BMI	2	2	1	2	1	5	5	3	1	1	2	2	3	1	1	4	1	2	1	1	2	5	1	1	4	1	2	2	1	2	2	2	4	2	1
Poor dietary diversity	15	16	16	3	13	6	3	14	6	8	7	5	19	6	9	15	3	4	3	5	6	15	8	6	20	8	5	17	7	5	15	18	5	18	19
No SBA	4	5	5	8	18	10	11	7	4	3	17	10	6	13	13	8	5	8	8	19	4	8	15	16	14	10	14	8	6	8	7	9	9	5	9
Less than four ANC visits	10	20	17	1	16	20	6	2	16	4	14	9	7	15	18	11	19	19	19	20	3	9	6	13	19	3	4	20	4	6	20	16	10	3	4
Unimproved sanitation	19	7	12	18	7	11	8	11	10	19	4	7	15	3	17	10	4	18	10	14	15	20	4	14	6	12	8	18	9	7	18	6	19	7	7
Had diarrhea, but not used ORT	5	11	6	7	5	7	7	4	19	9	5	6	9	12	5	1	11	10	6	4	20	12	10	10	12	2	18	3	5	4	10	5	11	15	17
Unsafe stool disposal	16	9	9	11	9	9	10	8	8	10	18	8	13	5	11	9	9	5	9	12	5	3	20	4	10	9	20	9	8	9	6	4	7	17	16
High indoor pollution	3	19	20	20	10	16	20	18	3	14	13	12	5	7	4	20	13	14	20	13	10	1	3	2	17	18	15	19	20	13	19	15	14	20	3
Not fully vaccinated	9	10	7	13	17	17	16	9	9	12	6	15	14	8	8	17	8	7	14	9	14	10	12	15	9	6	9	7	3	16	8	7	15	6	12
Infectious disease	7	6	18	16	15	15	15	19	12	11	15	13	10	2	16	19	10	13	12	11	7	19	14	5	15	20	19	12	12	20	11	17	20	9	11
Child marriage	14	15	13	19	11	13	17	16	18	18	19	14	18	16	14	7	14	11	15	16	9	18	19	12	11	17	16	11	18	11	17	10	17	10	8
Delayed breastfeeding	17	17	11	12	20	18	12	17	14	17	11	17	16	17	12	12	6	6	11	17	13	13	17	11	8	11	11	14	17	14	16	11	8	11	13
FP need unsatisfied	6	8	15	9	14	19	14	12	13	13	12	18	11	10	7	18	7	15	13	15	16	7	18	19	16	13	10	5	14	15	4	8	16	12	10
No vit A supplement	12	18	10	14	8	12	19	5	11	5	10	11	17	9	15	16	15	17	17	10	12	6	13	8	7	7	13	16	16	19	13	14	6	16	18
No iodized salt	20	14	4	10	2	4	9	15	7	6	9	19	20	11	20	14	20	12	16	18	17	17	9	17	5	15	12	13	13	10	9	13	13	19	5
Unsafe water	18	12	14	17	19	14	18	10	15	16	20	16	8	18	10	13	16	9	18	8	8	11	11	18	18	16	3	10	19	17	12	12	12	13	14
No care seeking for susp pneu	13	13	8	15	12	8	13	6	17	15	8	20	12	19	19	6	17	16	5	6	18	2	16	20	13	14	7	15	11	12	14	20	18	14	6

Label:
Rank 1st   Rank 2nd   Rank 3rd-4th   Rank 5th-7th   Rank 8th-12nd   Rank 13th-20th

© 2020 Li Z et al. *JAMA Network Open.*

D) Underweight, children living in urban areas, total n=81,347

	BJ	BF	BI	CM	CI	CD	ET	GM	GH	GN	HT	IN	KE	KG	KM	LS	LR	MW	ML	MZ	MM	NA	NP	NE	PE	ST	SL	SZ	TD	TJ	TZ	TG	UG	ZM	ZW
Short maternal stature	2	13	1	13	1	17	20	10	10	15	1	1	20	12	19	9	1	10	20	2	2	14	2	1	3	3	9	14	2	11	7	3	2	1	13
Lack of maternal education	3	7	19	3	9	5	7	8	1	9	14	3	13	4	1	14	17	16	1	4	19	7	16	6	5	8	12	8	5	13	4	7	1	5	10
Poorest HH wealth	4	5	5	15	10	2	10	19	4	2	18	4	2	9	20	12	12	13	18	11	18	12	12	20	2	1	17	15	3	20	6	4	4	2	11
Low maternal BMI	1	1	2	1	2	1	1	1	6	10	2	2	1	1	2	10	2	1	3	1	1	2	1	3	1	2	4	7	1	4	2	1	5	3	1
Poor dietary diversity	5	3	4	2	13	13	15	2	15	3	20	5	6	3	18	13	3	19	19	5	14	5	18	14	19	18	20	9	4	5	5	8	20	18	19
No SBA	8	6	6	11	11	7	13	6	14	8	13	6	15	13	4	4	7	5	13	16	9	6	13	9	7	15	16	18	8	10	20	10	6	12	8
Less than four ANC visits	6	20	14	20	18	20	6	3	3	5	7	7	12	11	5	1	5	20	9	3	4	19	10	12	10	19	6	10	6	2	8	2	10	4	16
Unimproved sanitation	15	12	16	16	7	18	12	4	13	11	9	13	11	15	14	18	9	15	10	13	5	9	4	13	12	16	5	13	14	1	10	14	19	13	17
Had diarrhea, but not used ORT	18	9	3	8	17	19	2	17	12	1	17	18	4	10	10	20	4	7	14	10	20	13	20	19	4	7	1	1	7	6	17	19	17	19	20
Unsafe stool disposal	19	8	9	17	15	16	5	14	5	18	5	8	5	8	12	6	11	2	5	6	15	11	6	4	16	10	2	6	11	8	14	16	7	17	14
High indoor pollution	13	2	12	12	8	12	11	11	11	4	3	17	3	16	16	3	14	11	2	15	11	4	5	10	9	20	14	3	9	14	1	5	9	6	7
Not fully vaccinated	14	14	8	4	4	15	8	20	16	13	12	9	7	19	6	17	16	14	6	19	7	10	17	11	20	13	7	20	16	19	9	12	15	16	15
Infectious disease	7	15	18	6	3	3	19	16	2	19	19	11	18	20	8	2	15	6	8	7	3	18	3	2	11	14	8	19	12	16	11	6	3	7	9
Child marriage	16	11	13	10	19	8	9	9	17	12	16	10	9	18	15	19	18	17	12	14	6	16	11	7	18	4	15	11	15	18	16	9	13	10	5
Delayed breastfeeding	9	17	17	19	16	10	17	5	18	20	8	14	17	7	7	5	8	8	11	18	13	8	15	15	17	9	11	2	17	9	15	17	14	8	6
FP need unsatisfied	12	16	11	18	5	14	4	12	20	7	11	16	8	6	9	8	6	4	17	8	16	17	14	5	13	17	10	17	10	7	12	11	18	14	2
No vit A supplement	11	10	7	7	12	6	18	15	8	6	15	15	10	5	13	15	13	18	7	20	10	15	9	16	8	6	13	4	18	17	19	15	16	20	18
No iodized salt	10	4	20	9	20	9	3	18	9	16	6	12	14	17	3	16	20	3	15	17	12	3	19	17	14	11	3	12	19	3	3	13	11	9	4
Unsafe water	20	18	10	5	14	4	16	7	7	17	10	20	19	2	11	7	19	9	4	9	8	20	7	8	6	5	18	5	20	15	18	18	12	15	3
No care seeking for susp pneu	17	19	15	14	6	11	14	13	19	14	4	19	16	14	17	11	10	12	16	12	17	1	8	18	15	12	19	16	13	12	13	20	8	11	12

Label:

Rank 1st　Rank 2nd　Rank 3rd-4th　Rank 5th-7th　Rank 8th-12nd　Rank 13th-20th

© 2020 Li Z et al. *JAMA Network Open.*

E) Wasting, children living in rural areas, total n=218,006

	BJ	BF	BI	CM	CI	CD	ET	GM	GH	GN	HT	IN	KE	KG	KM	LS	LR	MW	ML	MZ	MM	NA	NP	NE	PE	ST	SL	SZ	TD	TJ	TZ	TG	UG	ZM	ZW
Short maternal stature	15	4	20	6	12	2	2	3	11	5	3	4	10	9	4	12	5	2	13	4	19	11	20	1	7	19	20	12	2	12	1	12	9	4	1
Lack of maternal education	8	5	19	9	7	4	20	10	12	3	1	5	2	19	18	8	19	20	10	6	11	5	3	6	11	16	3	18	8	20	8	2	19	18	12
Poorest HH wealth	20	11	3	1	13	1	3	14	17	20	20	3	1	2	1	14	9	10	8	1	7	1	19	3	17	14	18	2	6	4	9	20	1	1	11
Low maternal BMI	1	2	1	7	1	12	1	6	1	2	2	1	4	3	9	1	2	1	1	5	1	6	5	2	12	4	2	11	3	1	3	1	2	11	3
Poor dietary diversity	14	1	17	2	16	9	4	13	20	1	13	2	19	7	2	6	18	4	16	2	8	3	2	16	20	8	1	7	12	8	5	19	13	16	4
No SBA	6	17	6	3	17	3	6	7	8	4	5	15	9	10	17	11	8	8	11	18	4	9	12	19	14	17	8	20	13	15	4	7	8	2	17
Less than four ANC visits	12	19	13	5	20	20	5	1	3	10	16	19	3	18	16	19	13	19	19	19	2	2	7	20	3	6	10	19	1	7	20	14	20	19	5
Unimproved sanitation	19	15	11	10	3	16	19	12	5	8	4	7	18	4	7	7	6	14	4	14	14	20	10	9	16	7	4	10	4	11	16	4	11	6	7
Had diarrhea, but not used ORT	2	6	4	8	8	6	7	9	7	6	8	10	7	8	3	9	3	11	7	15	18	13	4	4	18	3	17	13	7	2	19	18	18	5	9
Unsafe stool disposal	17	3	15	17	10	19	8	4	16	15	18	12	6	17	5	18	17	18	9	9	3	8	18	8	6	9	13	9	5	6	7	9	15	17	16
High indoor pollution	3	20	18	13	11	10	14	20	18	11	12	9	5	13	12	15	10	12	2	20	10	15	6	11	1	12	12	5	20	14	13	13	10	20	2
Not fully vaccinated	9	16	8	12	18	15	17	15	19	18	7	11	12	12	6	4	12	6	12	7	15	12	14	7	2	10	15	14	10	16	6	17	16	12	19
Infectious disease	16	10	10	20	5	5	16	19	15	7	14	14	14	20	19	16	20	5	6	8	12	16	16	17	5	20	9	8	17	19	2	8	7	14	14
Child marriage	7	12	7	18	4	14	11	11	13	13	19	17	15	6	14	13	16	9	14	16	13	10	9	13	9	11	7	17	11	3	18	5	12	9	8
Delayed breastfeeding	13	13	12	4	19	11	12	18	14	19	9	18	17	15	15	10	14	16	5	10	5	7	11	15	13	5	14	3	9	9	12	6	6	15	13
FP need unsatisfied	18	9	16	11	14	17	18	16	2	17	6	16	16	16	8	20	11	17	17	17	16	19	13	14	4	13	11	6	16	18	11	16	17	7	20
No vit A supplement	5	8	14	15	9	7	15	8	4	16	10	8	13	5	11	2	7	13	18	3	20	18	17	5	19	1	16	1	15	10	17	3	14	10	18
No iodized salt	11	18	2	16	2	8	9	17	10	9	11	13	20	11	20	17	1	3	15	12	17	17	8	18	15	2	19	4	19	5	10	11	3	3	6
Unsafe water	10	14	5	19	15	18	10	5	9	14	17	6	8	14	13	5	15	7	20	13	6	14	15	12	8	18	6	15	18	17	15	10	4	8	10
No care seeking for susp pneu	4	7	9	14	6	13	13	2	6	12	15	20	11	1	10	3	4	15	3	11	9	4	1	10	10	15	5	16	14	13	14	15	5	13	15

Label:
Rank 1st Rank 2nd Rank 3rd-4th Rank 5th-7th Rank 8th-12nd Rank 13th-20th

© 2020 Li Z et al. *JAMA Network Open.*

F) Wasting, children living in urban areas, total n=81,347

	BJ	BF	BI	CM	CI	CD	ET	GM	GH	GN	HT	IN	KE	KG	KM	LS	LR	MW	ML	MZ	MM	NA	NP	NE	PE	ST	SL	SZ	TD	TJ	TZ	TG	UG	ZM	ZW
Short maternal stature	1	11	3	11	10	4	20	8	12	14	13	5	16	11	20	NA	10	13	20	1	13	11	18	1	3	13	16	NA	20	15	12	1	8	20	10
Lack of maternal education	17	7	20	4	2	18	10	6	1	8	20	2	2	6	1	NA	15	10	5	5	18	14	7	16	15	3	3	NA	2	14	1	3	6	18	12
Poorest HH wealth	20	4	13	9	9	12	6	18	17	13	1	13	9	7	19	NA	2	3	1	3	6	15	8	14	18	18	18	NA	5	10	18	10	20	17	11
Low maternal BMI	2	6	1	3	5	1	1	2	18	9	12	1	5	2	12	NA	9	7	7	2	2	6	3	3	1	19	5	NA	1	4	8	2	11	2	1
Poor dietary diversity	9	2	10	5	15	16	3	3	11	2	14	4	6	19	3	NA	12	4	18	7	1	7	13	6	2	17	19	NA	8	20	19	16	12	3	9
No SBA	14	3	6	7	14	2	11	20	5	7	16	12	7	9	7	NA	7	16	19	6	19	8	10	8	6	4	15	NA	12	8	14	8	3	6	13
Less than four ANC visits	19	20	17	20	20	14	4	1	2	1	6	17	10	17	13	NA	3	9	12	20	15	3	16	13	14	1	7	NA	11	1	10	20	1	1	14
Unimproved sanitation	8	13	19	6	16	17	13	17	8	17	8	3	13	14	8	NA	19	18	3	13	14	5	2	9	7	7	11	NA	4	13	15	9	4	16	18
Had diarrhea, but not used ORT	13	8	2	14	19	20	2	19	4	4	18	6	1	13	2	NA	16	5	16	10	10	9	14	15	5	20	1	NA	7	18	20	6	7	19	8
Unsafe stool disposal	18	5	5	17	12	9	5	12	6	19	2	10	4	15	11	NA	8	14	11	4	8	12	5	2	20	8	2	NA	16	5	7	19	5	5	17
High indoor pollution	3	19	11	19	4	10	7	16	14	11	17	16	12	10	17	NA	11	19	2	18	16	17	9	4	12	16	12	NA	3	12	4	18	16	12	6
Not fully vaccinated	6	14	15	1	7	5	12	9	15	5	10	14	11	3	10	NA	14	15	14	8	4	19	15	18	10	11	8	NA	9	7	6	5	19	10	2
Infectious disease	4	9	14	2	1	6	17	4	20	20	9	11	20	20	9	NA	13	2	4	11	11	20	1	10	17	6	9	NA	6	19	17	11	18	8	7
Child marriage	16	12	18	16	8	7	8	13	10	10	11	8	18	16	16	NA	17	20	13	12	9	18	17	17	11	10	17	NA	18	16	16	4	14	4	5
Delayed breastfeeding	15	15	7	15	13	15	15	5	16	18	7	18	8	18	18	NA	4	17	9	15	7	2	11	20	8	9	13	NA	15	3	3	13	15	11	19
FP need unsatisfied	7	17	4	8	11	11	16	15	19	12	15	15	14	4	4	NA	5	11	6	16	20	16	12	5	13	15	6	NA	13	6	9	17	13	15	20
No vit A supplement	12	10	12	12	17	8	19	10	7	3	3	9	3	5	6	NA	6	6	8	14	17	13	19	7	9	5	10	NA	14	17	2	14	2	7	16
No iodized salt	5	1	9	13	18	19	9	14	9	6	4	7	19	12	14	NA	20	1	10	9	5	10	4	11	19	2	4	NA	19	2	11	12	9	9	3
Unsafe water	11	16	8	18	3	3	18	7	13	16	5	19	17	1	5	NA	18	12	17	19	12	1	6	12	4	12	20	NA	17	11	5	7	17	14	15
No care seeking for susp pneu	10	18	16	10	6	13	14	11	3	15	19	20	15	8	15	NA	1	8	15	17	3	4	20	19	16	14	14	NA	10	9	13	15	10	13	4

Label:

Rank 1st   Rank 2nd   Rank 3rd-4th   Rank 5th-7th   Rank 8th-12nd   Rank 13th-20th

© 2020 Li Z et al. *JAMA Network Open.*

eFigure 15. Country-Specific Odds Ratios for 20 Factors Associated With Child Anthropometric Failures From Fully Adjusted Models, Stratified by Place of Residence
A) stunting among children 12-23 months old, B) stunting among children 24-59 months old, C) underweight among children 12-23 months old, D) underweight among children 24-59 months old, E) wasting among children 12-23 months old, and F) wasting among children 24-59 months old

A) Stunting, children living in rural areas, total n=218,006

	BJ	BF	BI	CM	CI	CD	ET	GM	GH	GN	HT	IN	KE	KG	KM	LS	LR	MW	ML	MZ	MM	NA	NP	NE	PE	ST	SL	SZ	TD	TJ	TZ	TG	UG	ZM	ZW
Short maternal stature	8.8	6.6	9.4	3.9	1.8	3.4	3.0	2.3	3.6	0.7	5.6	4.4	6.0	3.5	1.4	4.7	3.7	7.3	1.3	4.2	4.5	2.7	28.9	1.2	11.3	18.4	2.7	9.4	1.1	4.1	5.6	5.3	9.9	5.2	9.5
Lack of maternal education	2.1	3.1	2.3	1.4	2.3	13.2	2.1	2.1	1.9	1.1	1.2	1.9	1.1	0.6	6.1	2.5	1.1	2.8	5.8	1.0	1.3	0.6	0.6	1.4	8.2	0.8	1.2	0.9	0.2	1.2	2.6	1.4	3.6	3.4	3.1
Poorest HH wealth	2.2	3.1	2.5	5.9	2.0	3.0	2.2	1.0	0.6	5.1	2.1	1.5	2.1	1.1	1.9	33.2	7.6	2.0	3.6	2.1	1.1	1.8	6.4	1.0	2.1	4.1	2.2	23.1	0.9	0.7	2.2	1.6	2.7	1.7	0.6
Low maternal BMI	1.8	1.8	2.0	1.6	2.9	1.7	1.2	1.4	1.7	0.8	1.7	1.7	1.5	2.6	2.2	4.3	1.5	2.2	2.1	1.5	1.8	1.5	1.4	1.6	0.5	1.9	1.4	10.1	1.5	1.6	1.8	2.1	1.4	1.9	2.0
Poor dietary diversity	1.0	1.4	1.0	2.4	1.6	1.2	2.5	0.7	1.0	1.3	1.2	1.2	1.0	1.1	0.9	0.9	1.4	1.1	2.1	1.4	0.8	1.0	0.6	1.6	1.4	3.5	1.3	0.5	1.3	1.8	1.1	1.1	1.4	1.1	0.9
No SBA	1.4	1.3	1.4	1.0	0.8	1.0	1.0	1.2	1.7	1.0	1.1	1.1	1.3	1.8	1.1	1.8	1.1	1.1	1.2	1.2	1.5	1.0	1.0	1.0	1.6	1.3	1.0	2.0	1.3	1.1	1.2	1.0	1.0	1.0	1.2
Less than four ANC visits	3.2	0.3	0.0	1.6	0.6	0.7	0.6	2.3	1.1	1.5	1.2	1.2	1.2	0.6	1.0	3.0	0.8	0.5	1.6	2.5	1.2	2.4	1.7	1.4	0.8	0.5	0.9	0.9	1.2	1.3	0.5	6.3	3.6	1.5	1.7
Unimproved sanitation	1.0	1.0	1.1	0.8	1.1	0.9	1.1	1.4	0.9	1.1	1.3	1.1	1.1	0.8	0.9	0.7	1.2	1.0	1.0	1.3	0.9	0.5	1.8	1.0	1.2	0.6	0.9	0.8	1.0	0.8	1.0	1.1	0.9	0.9	1.3
Had diarrhea, but not used ORT	1.4	1.1	1.0	1.2	0.8	1.2	1.2	0.9	0.8	1.0	1.3	1.1	1.0	0.5	1.5	11.0	0.8	1.3	1.2	1.1	0.9	1.6	1.4	0.8	0.9	1.8	0.5	6.1	1.2	1.5	1.4	1.1	1.0	0.9	0.7
Unsafe stool disposal	1.0	1.1	1.4	1.3	1.1	1.2	0.8	1.4	1.7	1.1	1.0	1.1	1.0	1.3	0.8	1.2	1.0	1.1	1.3	0.7	1.1	0.8	1.4	1.3	0.9	1.8	0.9	0.9	1.1	1.5	1.1	1.3	1.2	1.0	1.0
High indoor pollution	2.4	0.6	1.0	0.3	5.7	1.0	0.4	1.4	0.6	1.2	2.0	1.1	2.1	1.3	0.7	0.3	1.0	1.8	0.5	1.1	1.0	3.6	1.1	0.3	1.2	2.0	0.4	0.5	17.3	1.1	0.7	1.0	1.0	1.4	1.8
Not fully vaccinated	1.0	1.1	1.3	1.0	1.3	0.9	1.0	1.4	1.4	1.1	1.0	1.0	1.0	1.6	1.5	0.8	1.0	0.9	0.9	0.9	0.9	2.7	1.1	1.0	1.0	2.7	1.0	1.6	1.0	0.8	0.9	1.4	0.9	1.1	1.2
Infectious disease	1.1	1.1	1.0	0.9	1.0	0.9	1.1	0.9	1.2	1.3	1.0	1.1	0.9	1.3	1.1	0.3	1.3	0.8	0.9	1.3	1.0	0.9	1.1	1.4	1.0	2.2	0.8	0.7	1.1	0.7	0.9	0.9	0.7	1.1	1.3
Child marriage	1.1	0.9	1.1	0.8	1.3	1.1	0.9	0.9	1.1	1.0	0.9	1.1	1.1	0.9	1.1	1.0	1.0	0.9	1.0	0.9	1.1	0.5	0.7	1.0	1.0	1.4	1.0	1.2	1.0	0.8	0.8	1.2	0.9	1.1	1.1
Delayed breastfeeding	1.0	1.0	1.0	1.0	1.0	1.1	1.1	1.0	0.9	1.0	1.3	1.1	1.2	1.7	1.0	1.0	1.3	1.5	1.0	1.0	1.0	0.8	1.0	1.1	1.1	0.5	1.3	0.9	0.9	0.9	0.9	1.1	0.9	1.0	0.9
FP need unsatisfied	1.2	1.0	1.0	1.1	1.1	0.9	1.1	1.1	0.7	1.0	1.1	1.0	1.0	1.4	1.5	0.5	1.2	1.2	1.1	1.0	1.0	1.3	1.0	1.0	1.0	0.7	1.2	1.3	1.0	1.0	1.0	1.0	1.0	1.0	1.2
No vit A supplement	0.8	0.8	1.2	1.0	1.5	1.4	0.8	2.3	1.1	1.1	0.8	1.0	0.9	1.0	0.9	0.5	0.9	1.0	1.2	0.8	1.1	1.1	1.4	1.4	1.4	2.0	1.1	1.3	0.9	0.5	1.0	1.1	1.0	1.0	0.9
No iodized salt	0.9	0.8	0.8	1.6	1.5	1.2	0.9	0.9	0.9	1.1	1.0	0.9	1.0	0.9	1.3	1.1	0.8	0.9	1.1	1.0	1.1	0.6	0.6	1.0	1.0	0.8	1.4	1.0	1.0	0.8	1.0	1.0	0.7	0.8	1.0
Unsafe water	1.0	1.0	0.9	1.1	0.9	1.1	1.0	0.8	0.5	1.0	0.9	0.9	1.1	1.1	1.0	0.7	0.7	1.0	1.2	1.2	1.4	0.8	1.2	0.9	0.8	1.4	1.1	1.0	0.9	0.9	1.1	0.9	1.0	1.1	0.9
No care seeking for susp pneu	0.8	1.2	1.0	1.0	1.1	1.3	1.0	1.0	0.5	1.0	1.1	0.9	1.0	0.6	1.1	2.5	0.7	1.2	1.2	0.7	0.9	2.2	0.9	0.7	1.0	1.0	0.9	1.4	1.0	1.0	1.0	0.9	0.8	1.0	0.8

Label: odds ratios

>=4.0   [2.0, 4.0)   [1.3, 2.0)   [1.1, 1.3)   [1.0, 1.1)   <1.0

Note:

1. BJ= Benin, BF= Burkina Faso, BI= Burundi, CM=Cameroon, CI=Côte d'Ivoire, CD=The Democratic Republic of the Congo, ET=Ethiopia, GM=Gambia, GH=Ghana, GN=Guinea, HT=Haiti, IN=India, KE=Kenya, KG=Kyrgyzstan, KM=Comoros, LS=Lesotho, LR=Liberia, MW=Malawi, ML=Mali, MZ=Mozambique, MM=Myanmar, NA=Namibia, NP=Nepal, NE=Niger, PE=Peru, ST= São Tomé and Príncipe, SL=Sierra Leone, SZ=Swaziland, TD=Chad, TJ=Tajikistan, TZ=Tanzania, TG=Togo, UG=Uganda, ZM=Zambia, ZW=Zimbabwe.
2. Short maternal statue: maternal height <145cm; low maternal BMI: maternal BMI <18.5 kg/m2; child marriage: mother's age at marriage <18 years old; delayed breastfeeding: child was not initially breastfed within one hour after born; infectious disease: child was caught by infectious diseases two weeks prior to the survey.
3. Abbreviations - HH: household, BMI: body mass index; SBA: skilled birth attendant; ANC: antenatal care; ORT: oral rehydration therapy; FP: family planning; vit: vitamin; susp pneu: suspected pneumonia.

© 2020 Li Z et al. *JAMA Network Open.*

B) Stunting, children living in urban areas, total n=81,347

	BJ	BF	BI	CM	CI	CD	ET	GM	GH	GN	HT	IN	KE	KG	KM	LS	LR	MW	ML	MZ	MM	NA	NP	NE	PE	ST	SL	SZ	TD	TJ	TZ	TG	UG	ZM	ZW
Short maternal stature	7.5	1.0	16.4	1.0	30.2	6.5	0.6	1.0	114.3	0.6	1.0	4.6	0.2	3.4	0.6	0.3	3.4	33.1	0.1	7.3	50.1	13.1	5.3	1.0	8.7	1.6	1.1	1.0	3.2	6.3	7.3	1.0	92.6	4.4	4.2
Lack of maternal education	2.1	5.1	10.3	2.0	75.5	1.9	1.7	0.6	3.1	8.3	1.3	2.0	0.9	1.2	2.3	1.0	3.7	1.6	3.9	2.0	0.5	5.4	2.1	7.9	2.3	1.7	5.7	13.9	2.6	0.7	1.7	2.4	10.7	2.0	115.5
Poorest HH wealth	2.4	2.2	4.0	1.0	1.0	3.1	4.0	1.4	0.7	1.7	3.1	1.5	2.6	0.5	1.3	1.0	1.4	1.6	1.4	2.6	0.8	0.3	2.0	0.6	7.2	17.7	0.9	1.0	1.4	0.8	3.0	3.3	1.8	1.3	1.0
Low maternal BMI	2.2	2.5	3.1	1.6	2.2	1.4	8.1	0.7	0.9	1.5	2.2	1.5	1.1	4.8	1.8	1.0	3.2	3.0	1.2	2.1	2.2	14.7	1.5	1.6	4.3	5.4	1.9	1.0	2.1	1.5	1.8	4.0	3.9	2.2	2.9
Poor dietary diversity	0.9	2.2	6.5	1.3	0.5	1.5	1.0	1.3	0.7	2.2	0.7	1.1	1.6	2.5	0.3	1.2	1.1	0.2	1.7	1.6	1.4	0.2	0.6	1.2	0.9	0.8	0.2	0.4	1.3	1.7	3.2	3.6	0.6	0.9	1.9
No SBA	1.2	1.2	0.8	1.2	1.1	0.8	1.0	1.1	2.4	2.9	1.1	1.1	1.3	1.0	0.4	2.2	0.8	1.7	1.6	1.0	1.8	1.1	1.0	0.9	2.0	0.9	0.7	0.6	1.2	1.5	0.8	2.1	1.5	1.1	1.4
Less than four ANC visits	2.6	5.3	0.4	1.6	5.3	0.7	1.3	0.5	1.6	1.6	1.5	1.2	1.2	1.0	1.8	3.0	1.2	0.0	1.2	1.1	1.2	0.2	0.9	3.1	1.2	0.6	1.7	1.8	1.6	1.0	1.0	1.8	8.0	0.8	0.9
Unimproved sanitation	1.1	1.0	0.8	0.9	1.9	0.8	1.7	1.5	0.6	1.1	1.3	1.0	1.4	3.1	1.3	0.2	1.0	0.7	0.5	0.8	1.9	3.8	1.4	0.7	1.2	0.2	1.7	1.0	0.7	1.3	1.3	0.6	0.9	1.3	0.7
Had diarrhea, but not used ORT	1.0	1.0	0.6	1.0	1.1	0.9	0.2	0.5	0.2	3.5	0.5	1.1	1.3	0.9	1.2	5.9	1.8	3.0	1.5	0.9	1.0	10.1	0.3	0.8	1.1	0.8	0.3	1.0	1.1	1.7	0.9	0.5	0.5	1.5	0.7
Unsafe stool disposal	0.9	1.6	0.6	1.0	1.2	0.9	0.8	1.0	0.8	1.3	1.8	1.1	0.9	0.7	0.8	0.5	1.0	2.1	0.8	1.4	1.5	1.3	1.2	1.8	1.2	3.4	1.7	0.6	1.2	1.0	0.8	1.5	1.8	0.9	0.6
High indoor pollution	1.3	1.0	1.0	1.5	1.8	4.2	1.4	0.2	1.0	0.6	1.2	1.1	0.9	1.3	0.9	0.3	1.0	1.2	0.4	1.6	1.0	3.4	1.0	1.1	0.9	0.5	0.3	1.8	1.4	1.9	1.4	2.4	0.4	1.3	1.4
Not fully vaccinated	1.0	1.5	0.6	1.1	1.0	1.1	0.8	0.8	1.5	0.7	1.0	1.1	0.9	0.6	1.5	0.7	1.3	3.0	1.6	1.0	2.0	2.1	0.6	1.3	1.1	1.7	1.2	1.1	0.8	0.6	1.4	1.3	0.7	0.8	1.1
Infectious disease	0.9	0.9	1.4	1.4	0.9	1.3	1.2	0.6	1.0	0.9	0.8	1.0	1.0	1.5	1.2	0.5	0.8	0.5	1.0	1.0	0.9	2.8	1.4	1.2	0.8	3.2	1.3	1.1	0.9	1.2	0.8	2.4	2.0	0.8	1.2
Child marriage	1.1	0.9	1.2	1.3	0.6	1.3	0.8	1.5	1.4	0.7	1.3	1.0	1.2	1.4	1.1	1.0	0.5	1.1	1.2	1.2	2.5	1.4	1.4	1.4	1.1	1.3	0.8	1.1	0.9	0.9	0.8	0.7	0.5	1.2	1.2
Delayed breastfeeding	1.1	0.7	1.7	0.9	0.5	1.2	1.2	0.8	0.6	0.6	0.7	1.1	1.0	0.5	0.8	3.9	1.3	0.5	1.3	0.7	1.7	2.5	0.9	0.6	0.8	1.8	1.0	5.3	1.0	0.8	0.8	0.9	1.5	1.0	1.4
FP need unsatisfied	1.3	1.1	0.3	1.1	1.1	1.1	2.2	1.5	0.9	0.7	1.3	0.9	0.8	1.3	0.6	1.0	0.8	1.6	1.0	1.0	1.4	1.3	0.9	0.8	1.1	0.5	1.6	1.6	1.2	0.8	0.7	1.2	0.4	1.0	0.4
No vit A supplement	0.9	1.1	0.5	1.6	1.2	1.3	0.5	0.4	1.2	2.6	1.0	1.0	0.9	1.1	1.0	0.8	1.5	1.4	1.1	0.6	1.7	0.9	0.9	0.9	1.3	0.9	1.1	3.5	0.9	1.3	0.7	1.1	0.4	0.8	1.2
No iodized salt	0.9	1.7	0.4	1.0	1.6	0.7	2.3	0.7	1.3	1.1	1.2	1.1	0.8	0.5	0.8	1.5	0.4	0.7	0.9	0.7	0.8	1.1	0.6	0.6	0.9	0.5	1.0	24.6	0.9	0.9	2.2	0.6	94.2	1.2	0.5
Unsafe water	0.9	0.5	3.9	1.9	0.6	1.0	0.8	0.8	0.9	2.5	0.6	1.0	1.1	1.8	0.8	4.7	1.0	0.6	1.2	0.8	0.8	0.5	1.1	0.7	0.9	0.4	1.1	10.5	0.9	2.1	1.1	1.6	0.8	1.0	0.8
No care seeking for susp pneu	1.2	0.4	1.5	1.0	2.2	1.2	1.0	0.8	0.7	1.0	1.4	0.9	1.0	0.5	0.7	0.8	0.9	0.5	1.3	1.0	0.9	0.3	0.6	0.5	1.0	1.0	0.3	11.2	1.0	1.0	1.0	0.7	0.1	1.0	1.5

Label: odds ratios

- >=4.0
- [2.0, 4.0)
- [1.3, 2.0)
- [1.1, 1.3)
- [1.0, 1.1)
- <1.0

© 2020 Li Z et al. *JAMA Network Open.*

C) Underweight, children living in rural areas, total n=218,006

	BJ	BF	BI	CM	CI	CD	ET	GM	GH	GN	HT	IN	KE	KG	KM	LS	LR	MW	ML	MZ	MM	NA	NP	NE	PE	ST	SL	SZ	TD	TJ	TZ	TG	UG	ZM	ZW
Short maternal stature	4.8	4.9	3.7	2.2	2.1	4.0	4.2	6.5	3.9	2.3	5.1	3.4	6.4	1.0	2.0	5.9	2.8	6.0	1.3	3.4	4.4	1.0	2.4	1.4	4.3	3.4	2.2	6.2	1.1	2.2	8.1	10.9	7.2	3.9	2.8
Lack of maternal education	1.1	1.5	0.7	2.5	2.5	3.0	36.3	0.4	1.6	1.4	3.3	1.7	2.4	0.3	1.7	5.8	1.1	0.5	1.5	1.3	0.6	0.6	1.6	1.5	3.5	0.3	1.5	1.6	1.6	0.8	1.3	1.9	9.8	1.2	0.9
Poorest HH wealth	1.2	1.8	2.8	2.6	1.4	3.3	2.0	1.0	0.2	0.3	1.0	1.6	4.6	2.2	2.4	2.0	0.6	2.4	2.2	2.6	1.0	2.2	2.0	1.3	8.8	3.2	0.9	2.3	0.9	5.2	3.0	0.8	4.3	2.3	0.4
Low maternal BMI	3.3	4.4	6.6	4.0	7.4	2.0	1.8	2.0	9.0	2.9	4.5	2.6	4.0	6.1	2.8	4.8	4.6	2.9	2.4	4.3	3.3	2.1	3.2	3.1	3.3	8.6	1.7	3.8	2.7	4.0	3.3	4.7	3.5	3.6	5.7
Poor dietary diversity	1.0	0.9	1.0	2.9	1.0	1.6	2.5	0.9	1.4	1.4	1.3	1.4	0.7	1.7	1.4	0.5	1.9	1.5	1.9	1.8	1.4	0.8	1.5	1.4	0.4	2.4	1.6	0.7	1.2	2.1	1.0	0.9	1.9	0.8	0.6
No SBA	1.7	1.3	1.5	1.4	0.7	1.2	1.0	1.4	1.7	1.6	0.9	1.1	1.4	1.0	1.1	1.1	1.3	1.2	1.3	0.8	1.6	1.5	1.0	1.0	1.0	1.6	1.0	1.2	1.4	1.2	1.3	1.2	1.2	1.5	1.2
Less than four ANC visits	1.1	0.2	0.8	4.7	0.9	0.7	1.7	2.5	0.7	1.5	1.0	1.1	1.2	0.9	0.7	0.9	0.6	0.6	0.7	0.4	1.7	1.4	1.8	1.0	0.7	5.5	1.6	0.2	1.5	1.2	0.3	1.0	1.1	2.6	1.7
Unimproved sanitation	0.9	1.2	1.1	0.9	1.3	1.2	1.2	1.1	1.2	0.9	1.8	1.2	0.9	4.3	0.8	0.9	1.3	0.7	1.1	1.0	0.9	0.1	2.1	1.0	1.5	1.0	1.3	0.6	1.1	1.2	0.8	1.4	0.8	1.3	1.3
Had diarrhea, but not used ORT	1.3	1.1	1.4	1.5	1.5	1.4	1.6	1.8	0.5	1.2	1.4	1.2	1.1	1.0	1.7	6.8	1.1	1.1	1.3	1.8	0.5	1.2	1.2	1.2	1.0	6.0	0.9	3.6	1.4	2.1	1.2	1.5	1.1	1.0	0.9
Unsafe stool disposal	1.0	1.1	1.2	1.2	1.2	1.2	1.1	1.2	1.2	1.2	0.9	1.1	1.0	1.8	1.1	1.1	1.2	1.3	1.2	1.0	1.5	2.9	0.7	1.4	1.1	2.2	0.7	1.1	1.1	1.2	1.3	1.8	1.4	0.9	0.9
High indoor pollution	2.7	0.2	0.3	0.4	1.1	1.0	0.4	0.6	3.6	1.0	1.0	1.1	1.7	1.4	2.0	0.2	1.0	1.0	0.6	1.0	1.1	8.3	2.1	1.6	0.8	0.7	1.0	0.4	0.5	1.0	0.5	1.0	1.0	0.8	2.5
Not fully vaccinated	1.1	1.1	1.3	1.1	0.9	1.0	0.9	1.2	1.2	1.1	1.3	1.0	0.9	1.4	1.5	0.4	1.3	1.3	1.0	1.2	0.9	1.3	1.2	1.0	1.2	2.9	1.3	1.4	1.6	0.9	1.2	1.4	1.0	1.3	1.1
Infectious disease	1.2	1.2	0.8	1.0	1.0	1.0	0.9	0.5	0.9	1.1	1.0	1.0	1.1	4.7	0.9	0.3	1.1	1.0	1.0	1.1	1.4	0.2	1.0	1.4	0.9	0.2	0.8	1.0	1.0	0.6	1.2	0.9	0.6	1.2	1.1
Child marriage	1.0	1.0	1.1	0.8	1.1	1.1	0.9	0.9	0.5	0.9	0.8	1.0	0.8	0.7	1.0	1.1	1.0	1.1	1.0	0.9	1.2	0.4	0.7	1.0	1.1	0.8	1.0	1.0	0.9	1.0	0.9	1.2	0.9	1.1	1.2
Delayed breastfeeding	1.0	0.9	1.2	1.1	0.6	0.9	1.0	0.7	0.8	1.0	1.1	1.0	0.9	0.6	1.1	0.8	1.3	1.3	1.0	0.9	0.9	1.2	0.9	1.1	1.2	1.1	1.1	0.8	0.9	0.9	0.9	1.1	1.2	1.1	1.0
FP need unsatisfied	1.2	1.2	1.0	1.3	1.0	0.8	1.0	1.0	0.8	1.1	1.0	1.0	1.0	1.1	1.5	0.4	1.3	0.9	1.0	0.9	0.9	1.7	0.8	0.9	0.9	1.0	1.2	2.1	0.9	0.9	1.4	1.2	0.9	1.1	1.1
No vit A supplement	1.1	0.9	1.2	1.0	1.3	1.1	0.8	1.7	1.1	1.4	1.1	1.1	0.9	1.3	0.9	0.5	0.9	0.8	0.9	1.2	1.0	2.1	1.2	1.4	1.4	2.8	1.1	0.7	0.9	0.7	1.0	1.0	1.4	0.9	0.8
No iodized salt	0.8	1.0	2.2	1.2	2.6	2.3	1.1	0.9	1.3	1.4	1.1	0.9	0.7	1.0	0.4	0.6	0.4	1.0	1.0	0.9	0.8	0.5	1.3	1.0	1.8	1.0	1.1	0.9	0.9	1.0	1.2	1.0	1.0	0.8	1.4
Unsafe water	0.9	1.1	1.1	0.9	0.6	1.0	0.9	1.1	0.8	1.0	0.8	1.0	1.1	0.5	1.2	0.7	0.9	1.2	0.9	1.2	1.2	1.3	1.2	0.9	0.7	0.9	1.7	1.1	0.8	0.8	1.0	1.0	1.1	1.0	1.0
No care seeking for susp pneu	1.0	1.0	1.3	1.0	1.1	1.2	1.0	1.6	0.5	1.0	1.1	0.9	1.0	0.4	0.6	1.7	0.8	0.9	1.5	1.4	0.8	5.8	1.0	0.6	1.0	1.0	1.5	0.7	1.0	1.0	1.0	0.8	0.9	1.0	1.4

Label: odds ratios

>=4.0 [2.0, 4.0) [1.3, 2.0) [1.1, 1.3) [1.0, 1.1) <1.0

© 2020 Li Z et al. *JAMA Network Open.*

D) Underweight, children living in urban areas, total n=81,347

	BJ	BF	BI	CM	CI	CD	ET	GM	GH	GN	HT	IN	KE	KG	KM	LS	LR	MW	ML	MZ	MM	NA	NP	NE	PE	ST	SL	SZ	TD	TJ	TZ	TG	UG	ZM	ZW
Short maternal stature	3.0	1.0	14.5	1.0	10.4	0.8	0.2	1.0	1.0	1.0	35.4	3.4	0.6	1.0	0.3	1.0	40.4	1.0	0.1	6.9	4.5	1.0	2.8	3.5	10.7	6.3	1.5	1.0	2.6	1.0	1.7	5.0	7.0	6.2	1.0
Lack of maternal education	2.1	1.7	0.2	3.5	1.0	1.4	2.0	1.1	8.0	1.2	0.9	1.8	1.1	2.4	13.3	1.0	0.7	0.5	3.8	2.3	0.3	3.4	0.9	1.4	1.7	2.0	1.2	1.0	1.5	1.0	2.3	2.5	12.3	1.7	1.0
Poorest HH wealth	1.6	1.9	3.1	1.0	1.0	2.7	1.2	0.5	1.7	6.6	0.5	1.6	2.9	1.0	0.2	1.0	1.1	0.9	0.6	1.2	0.3	1.0	1.1	0.3	12.9	26.4	0.5	1.0	2.4	0.3	1.8	4.0	4.9	2.8	1.0
Low maternal BMI	4.7	2.7	8.9	13.4	6.4	7.4	22.0	2.1	1.2	1.2	9.9	2.4	3.3	5.1	5.1	1.0	8.5	39.7	2.3	10.9	7.5	8.9	3.6	1.8	14.4	9.5	3.1	1.0	3.5	1.4	2.9	7.8	3.5	2.3	5.4
Poor dietary diversity	1.4	2.5	3.3	8.0	0.7	1.0	0.8	1.8	0.8	5.0	0.0	1.3	1.7	2.8	0.4	1.0	8.4	0.1	0.6	1.8	0.8	5.1	0.7	0.9	0.7	0.4	0.1	1.0	1.7	1.4	2.1	2.4	0.1	0.8	0.2
No SBA	1.3	1.7	2.3	1.3	0.9	1.1	1.1	1.2	0.8	2.0	1.2	1.2	1.0	1.0	2.4	12.2	1.4	7.9	1.0	0.8	1.4	4.3	1.0	1.1	1.6	0.8	0.7	0.0	1.2	1.1	0.5	1.2	3.1	1.0	1.2
Less than four ANC visits	1.3	0.1	0.8	0.6	0.4	0.2	2.2	1.5	1.8	2.8	2.6	1.2	1.1	1.0	2.3	29.5	2.4	0.1	1.3	5.0	2.1	0.0	1.1	1.0	1.4	0.3	1.9	1.0	1.4	2.3	1.6	5.7	1.0	1.8	0.4
Unimproved sanitation	1.0	1.1	0.6	1.0	1.6	0.7	1.2	1.4	0.9	1.1	1.7	1.0	1.1	1.0	1.1	0.1	1.3	0.6	1.1	1.0	1.9	1.6	1.5	0.9	1.3	0.5	2.5	1.0	1.0	8.8	1.2	0.7	0.2	1.0	0.3
Had diarrhea, but not used ORT	0.9	1.4	6.2	1.8	0.5	0.6	8.8	0.6	0.9	8.7	0.5	1.0	1.9	1.0	1.5	0.0	3.3	1.3	0.9	1.2	0.1	1.0	0.5	0.4	1.8	2.0	13.7	1.0	1.3	1.3	0.6	0.5	0.2	0.7	0.2
Unsafe stool disposal	0.8	1.6	1.8	0.9	0.6	0.9	3.0	0.8	1.7	0.6	5.0	1.1	1.9	1.4	1.4	7.0	1.2	16.7	2.0	1.7	0.7	1.3	1.3	1.6	1.0	1.3	5.1	1.0	1.1	1.2	0.9	0.6	1.2	0.8	0.6
High indoor pollution	1.0	2.6	1.0	1.1	1.5	1.0	1.2	0.9	0.9	4.1	8.2	1.0	2.1	0.8	0.6	17.0	1.0	1.0	3.3	0.9	1.2	5.2	1.3	1.1	1.4	0.2	1.0	1.0	1.2	1.0	2.9	3.6	1.0	1.7	1.3
Not fully vaccinated	1.0	1.0	2.0	2.6	2.4	1.0	1.9	0.5	0.7	1.1	1.2	1.0	1.5	0.1	2.2	0.1	0.9	0.7	1.5	0.6	1.6	1.3	0.8	1.0	0.7	0.9	1.8	0.0	0.9	0.5	1.3	1.1	0.4	0.9	0.5
Infectious disease	1.3	1.0	0.4	2.1	3.4	2.1	0.2	0.6	2.4	0.4	0.2	1.0	0.9	0.0	1.6	17.8	0.9	4.5	1.3	1.6	2.2	0.1	1.8	2.2	1.4	0.8	1.7	0.0	1.0	0.9	1.1	3.0	5.6	1.5	1.1
Child marriage	0.9	1.2	1.0	1.7	0.4	1.1	1.5	1.0	0.6	1.1	0.7	1.0	1.2	0.2	0.8	0.0	0.7	0.4	1.0	0.9	1.8	0.8	1.1	1.4	0.8	3.3	0.8	1.0	0.9	0.6	0.8	1.8	0.5	1.0	1.9
Delayed breastfeeding	1.1	0.7	0.5	0.7	0.5	1.0	0.5	1.3	0.6	0.4	1.8	1.0	1.0	1.4	1.9	10.4	1.4	1.1	1.1	0.7	1.0	3.0	0.9	0.9	0.9	1.9	1.4	1.0	0.8	1.2	0.9	0.5	0.4	1.3	1.3
FP need unsatisfied	1.1	0.8	1.0	0.8	1.9	1.0	3.5	0.9	0.6	2.1	1.3	1.0	1.2	1.8	1.5	1.3	1.8	12.0	0.8	1.4	0.5	0.2	1.0	1.5	1.2	0.4	1.5	0.0	1.2	1.3	1.1	1.2	0.2	0.9	2.9
No vit A supplement	1.1	1.3	2.3	2.0	0.7	1.2	0.3	0.6	1.0	2.6	0.9	1.0	1.1	1.8	1.4	0.5	1.0	0.3	1.4	0.3	1.2	0.9	1.2	0.8	1.5	2.2	1.1	1.0	0.8	0.8	0.6	0.7	0.2	0.6	0.3
No iodized salt	1.1	2.1	0.2	1.7	0.3	1.1	3.9	0.5	1.0	0.9	3.4	1.0	1.0	0.5	3.8	0.4	0.2	16.1	0.9	0.7	1.2	6.3	0.7	0.7	1.0	1.3	3.3	1.0	0.6	2.1	2.5	0.7	0.9	1.2	2.4
Unsafe water	0.8	0.4	1.7	2.2	0.6	1.7	0.5	1.1	1.0	0.7	1.4	0.8	0.8	3.0	1.5	2.7	0.4	1.1	2.1	1.2	1.6	0.0	1.2	1.2	1.7	3.1	0.5	1.0	0.5	1.0	0.6	0.5	0.9	0.9	2.8
No care seeking for susp pneu	0.9	0.2	0.8	1.0	1.6	1.0	1.0	0.8	0.6	1.0	7.2	0.9	1.0	1.0	0.6	1.0	1.2	1.0	0.8	1.0	0.3	15.6	1.2	0.5	1.0	1.0	0.2	1.0	1.0	1.0	1.0	0.2	1.0	1.0	1.0

Label: odds ratios

>=4.0 [2.0, 4.0) [1.3, 2.0) [1.1, 1.3) [1.0, 1.1) <1.0

© 2020 Li Z et al. *JAMA Network Open.*

E) Wasting, children living in rural areas, total n=218,006

	BJ	BF	BI	CM	CI	CD	ET	GM	GH	GN	HT	IN	KE	KG	KM	LS	LR	MW	ML	MZ	MM	NA	NP	NE	PE	ST	SL	SZ	TD	TJ	TZ	TG	UG	ZM	ZW
Short maternal stature	1.0	1.7	0.3	1.7	1.0	1.7	1.7	1.9	1.0	1.6	2.2	1.2	1.0	1.0	2.0	0.4	1.6	3.8	1.0	1.8	0.5	1.0	0.2	3.2	1.4	0.6	0.1	1.0	6.0	1.0	2.5	1.0	1.0	1.9	6.0
Lack of maternal education	1.2	1.5	0.4	1.3	1.2	1.4	0.2	1.1	0.9	1.8	6.2	1.2	4.0	0.1	0.6	1.0	0.5	0.1	1.1	1.5	1.0	3.1	3.5	1.2	1.0	0.9	1.9	0.1	1.3	0.6	1.2	2.4	0.4	0.6	1.0
Poorest HH wealth	0.6	1.0	1.7	3.7	1.0	2.1	1.6	0.9	0.4	0.1	0.4	1.2	9.4	3.3	3.7	0.2	1.1	1.3	1.2	2.1	1.1	18.3	0.2	1.7	0.4	1.0	0.7	12.8	1.5	1.6	1.1	0.5	17.6	2.9	1.0
Low maternal BMI	13.0	3.0	14.9	1.7	18.7	1.0	2.2	1.6	5.9	2.3	3.2	2.3	1.7	2.7	1.4	5.9	4.4	4.4	2.6	1.5	2.0	2.7	2.6	2.7	1.0	2.8	2.1	1.0	4.0	4.6	2.4	5.8	14.1	1.0	4.1
Poor dietary diversity	1.1	6.4	0.6	2.5	0.6	1.2	1.5	0.9	0.1	5.6	0.9	1.3	0.5	1.3	3.0	1.0	0.5	1.7	0.9	1.9	1.1	6.0	6.0	0.9	0.1	1.6	3.8	2.2	1.1	1.3	2.0	0.6	0.8	0.8	2.5
No SBA	1.5	0.8	1.5	2.5	0.5	1.6	1.3	1.5	1.6	1.8	1.4	1.0	1.1	1.0	0.6	0.9	1.1	1.4	1.1	0.4	1.3	2.2	1.0	0.8	0.7	0.8	1.2	0.0	1.1	0.9	2.2	1.3	1.1	2.1	0.6
Less than four ANC visits	1.1	0.4	1.0	2.1	0.1	0.6	1.5	6.1	2.7	1.1	0.7	0.9	2.9	0.2	0.8	0.1	0.9	0.7	0.7	0.3	1.9	6.7	2.0	0.7	2.4	2.1	1.1	0.1	8.2	1.3	0.1	1.0	0.3	0.5	2.5
Unimproved sanitation	0.8	0.9	1.1	1.2	1.9	0.8	0.7	1.0	2.6	1.3	1.5	1.1	0.6	2.2	1.5	1.0	1.5	0.9	1.2	0.9	0.9	0.1	1.2	1.1	0.7	1.6	1.6	1.5	2.7	1.0	0.8	1.6	0.9	1.5	1.3
Had diarrhea, but not used ORT	2.0	1.4	1.6	1.6	1.2	1.3	1.2	1.2	2.0	1.4	1.1	1.0	1.1	1.0	2.1	1.0	3.3	1.1	1.2	0.9	0.8	0.9	2.8	1.4	0.3	4.0	0.7	1.0	1.3	4.0	0.5	0.8	0.6	1.6	1.1
Unsafe stool disposal	0.9	1.8	1.0	0.8	1.0	0.7	1.1	1.8	0.5	0.8	0.6	1.0	1.2	0.5	1.7	0.1	0.7	0.7	1.1	1.3	1.4	2.6	0.5	1.1	1.7	1.5	1.0	1.6	1.8	1.3	1.3	1.1	0.8	0.7	0.7
High indoor pollution	2.0	0.2	0.6	1.0	1.0	1.0	1.0	0.3	0.3	1.0	1.0	1.0	1.6	0.9	1.0	0.1	1.0	1.0	1.5	0.3	1.0	0.6	2.4	1.0	3.6	1.1	1.0	2.7	0.2	1.0	1.0	1.0	1.0	0.3	4.8
Not fully vaccinated	1.2	0.8	1.3	1.1	0.4	0.9	0.8	0.8	0.3	0.6	1.2	1.0	1.0	1.0	1.5	1.3	0.9	1.5	1.0	1.5	0.8	0.9	0.9	1.2	2.7	1.4	0.9	1.0	1.2	0.9	1.4	0.8	0.6	1.0	0.3
Infectious disease	0.9	1.0	1.1	0.7	1.5	1.3	0.9	0.4	0.6	1.3	0.9	1.0	0.9	0.0	0.6	0.1	0.4	1.6	1.2	1.4	1.0	0.6	0.7	0.8	1.7	0.1	1.1	2.1	0.8	0.7	2.5	1.2	1.1	0.9	0.8
Child marriage	1.3	0.9	1.4	0.8	1.8	0.9	1.0	1.0	0.8	0.8	0.4	0.9	0.9	1.3	0.8	0.3	0.7	1.4	0.9	0.7	1.0	1.3	1.2	1.0	1.1	1.2	1.5	0.1	1.2	1.7	0.7	1.4	0.9	1.0	1.1
Delayed breastfeeding	1.1	0.9	1.0	2.2	0.4	1.0	1.0	0.8	0.7	0.5	1.1	0.9	0.6	0.7	0.8	0.9	0.9	0.8	1.2	1.1	1.3	2.7	1.0	0.9	0.9	2.6	1.0	3.5	1.3	1.2	1.0	1.3	1.1	0.9	0.9
FP need unsatisfied	0.8	1.3	0.9	1.1	0.9	0.8	0.8	0.8	3.0	0.7	1.2	1.0	0.8	0.6	1.4	0.0	1.0	0.8	0.8	0.7	0.8	0.2	1.0	0.9	2.0	1.0	1.1	2.3	0.8	0.8	1.0	0.9	0.6	1.2	0.3
No vit A supplement	1.5	1.3	1.0	0.9	1.0	1.2	0.9	1.3	2.6	0.8	1.0	1.1	0.9	1.9	1.1	3.9	1.3	0.9	0.8	1.9	0.5	0.3	0.6	1.2	0.2	7.4	0.7	14.2	0.9	1.1	0.8	1.7	0.8	1.0	0.6
No iodized salt	1.2	0.5	2.3	0.8	2.3	1.2	1.1	0.8	1.0	1.2	1.0	1.0	0.5	1.0	0.4	0.1	4.6	3.6	0.9	0.9	0.8	0.4	1.6	0.8	0.7	5.2	0.6	3.4	0.7	1.4	1.1	1.0	2.4	2.1	1.5
Unsafe water	1.2	0.9	1.6	0.7	0.8	0.8	1.1	1.8	1.3	0.8	0.7	1.2	1.1	0.7	1.0	1.1	0.8	1.5	0.7	0.9	1.2	0.8	0.8	1.0	1.4	0.7	1.5	0.6	0.8	0.9	1.0	1.1	1.5	1.1	1.1
No care seeking for susp pneu	2.0	1.4	1.2	1.0	1.3	0.9	1.0	2.4	2.2	1.0	0.9	0.9	1.0	26.7	1.3	1.5	2.5	0.9	1.4	1.0	1.1	5.6	6.6	1.0	1.0	1.0	1.6	0.5	1.0	1.0	1.0	1.0	1.2	1.0	0.8

Label: odds ratios

>=4.0 [2.0, 4.0) [1.3, 2.0) [1.1, 1.3) [1.0, 1.1) <1.0

© 2020 Li Z et al. *JAMA Network Open.*

F) Wasting, children living in urban areas, total n=81,347

	BJ	BF	BI	CM	CI	CD	ET	GM	GH	GN	HT	IN	KE	KG	KM	LS	LR	MW	ML	MZ	MM	NA	NP	NE	PE	ST	SL	SZ	TD	TJ	TZ	TG	UG	ZM	ZW
Short maternal stature	8.2	1.0	13.4	1.0	1.0	1.5	0.0	1.0	1.0	1.0	1.0	1.1	1.0	1.0	0.4	NA	1.0	1.0	0.3	8.8	0.8	1.0	0.5	4.6	6.9	1.0	0.7	NA	0.0	1.0	1.0	7.2	1.0	0.3	1.0
Lack of maternal education	0.7	1.5	0.1	3.3	2.4	0.4	2.2	1.3	49.0	1.2	0.1	1.2	3.3	2.3	4.5	NA	0.7	1.0	1.7	1.2	0.4	1.0	1.5	0.9	1.0	3.9	3.9	NA	2.2	1.0	4.3	2.6	1.0	0.6	1.0
Poorest HH wealth	0.2	2.5	1.0	1.0	1.0	1.0	2.8	0.4	0.5	1.0	45.9	1.0	1.4	1.0	0.4	NA	7.2	37.5	16.3	2.7	1.6	1.0	1.4	1.0	0.7	0.6	0.3	NA	1.4	1.0	0.5	1.0	0.0	0.7	1.0
Low maternal BMI	4.6	1.8	35.1	3.6	2.0	4.6	10.9	10.5	0.4	1.1	1.1	2.2	2.2	5.0	0.8	NA	1.0	1.6	1.2	6.3	4.1	1.0	2.4	2.6	16.8	0.3	3.2	NA	6.4	1.9	1.4	2.9	0.4	1.9	9.9
Poor dietary diversity	1.1	3.0	1.0	3.0	0.5	0.7	8.0	5.0	1.0	3.7	1.0	1.1	2.1	0.3	2.1	NA	1.0	17.6	0.5	1.1	4.2	1.0	0.9	1.8	9.3	0.7	0.3	NA	1.4	0.5	0.3	0.5	0.1	1.6	1.0
No SBA	0.9	2.9	1.9	1.9	0.6	2.3	1.5	0.1	1.8	1.3	0.6	1.0	1.8	1.0	1.4	NA	1.5	0.3	0.4	1.2	0.3	1.0	1.0	1.8	2.2	2.7	0.7	NA	1.0	1.1	0.9	1.1	6.7	1.4	0.6
Less than four ANC visits	0.5	0.0	0.2	0.2	0.1	0.9	5.4	46.6	45.5	5.6	2.3	1.0	1.3	0.8	0.8	NA	3.9	1.0	0.8	0.3	0.7	1.0	0.7	1.2	1.0	6.9	2.2	NA	1.0	5.2	1.3	0.2	49.0	2.3	0.5
Unimproved sanitation	1.4	0.9	0.1	2.3	0.5	0.6	1.1	0.6	1.2	0.5	1.3	1.2	1.1	1.0	1.3	NA	0.2	0.1	2.2	0.8	0.7	1.0	2.4	1.5	2.0	1.8	1.1	NA	1.5	1.0	0.8	1.0	4.7	0.8	0.2
Had diarrhea, but not used ORT	0.9	1.3	14.8	0.7	0.2	0.2	10.0	0.3	5.7	1.7	0.3	1.1	5.0	1.0	2.1	NA	0.7	2.6	0.5	1.0	1.0	1.0	0.8	0.9	2.8	0.3	13.8	NA	1.4	0.6	0.2	1.5	1.0	0.5	1.0
Unsafe stool disposal	0.6	1.8	9.8	0.6	0.9	1.0	4.8	0.8	1.7	0.3	9.3	1.0	2.6	0.9	0.9	NA	1.2	1.0	1.0	1.3	1.2	1.0	2.3	3.0	0.3	1.4	6.5	NA	0.8	1.4	1.5	0.2	1.4	1.5	0.2
High indoor pollution	2.6	0.7	1.0	0.4	2.1	1.0	2.8	0.7	0.7	1.0	0.4	1.0	1.1	1.0	0.6	NA	1.0	0.0	15.9	0.5	0.7	0.0	1.0	2.2	1.2	0.7	1.0	NA	1.7	1.0	2.8	0.5	0.0	1.0	1.4
Not fully vaccinated	1.5	0.8	0.6	6.8	1.3	1.3	1.4	1.0	0.7	1.4	1.2	1.0	1.3	3.7	1.1	NA	0.8	0.9	0.6	1.1	2.0	0.0	0.8	0.5	1.5	1.2	1.5	NA	1.1	1.2	2.4	2.1	0.0	1.1	4.4
Infectious disease	2.1	1.2	0.8	4.0	3.9	1.3	0.4	2.2	0.0	0.2	1.3	1.0	0.4	0.0	1.3	NA	1.0	42.4	2.1	0.9	1.0	0.0	2.9	1.5	1.0	2.1	1.2	NA	1.4	0.5	0.5	0.8	0.0	1.2	1.2
Child marriage	0.8	1.0	0.1	0.7	1.0	1.1	2.5	0.8	1.1	1.0	1.2	1.0	0.7	0.8	0.7	NA	0.5	0.0	0.7	0.8	1.1	0.0	0.6	0.7	1.3	1.2	0.7	NA	0.7	0.7	0.6	2.5	0.0	1.5	1.6
Delayed breastfeeding	0.9	0.8	1.2	0.7	0.7	0.7	0.5	1.9	0.6	0.5	2.2	1.0	1.7	0.4	0.6	NA	2.6	0.1	1.0	0.7	1.2	1.0	1.0	0.5	1.8	1.3	0.7	NA	0.9	2.1	3.1	0.8	0.0	1.1	0.2
FP need unsatisfied	1.4	0.8	10.9	1.2	0.9	1.0	0.5	0.7	0.1	1.0	0.9	1.0	1.0	3.0	1.5	NA	2.0	1.0	1.3	0.6	0.3	1.0	0.9	2.0	1.0	1.0	2.2	NA	0.9	1.2	1.4	0.5	0.0	0.9	0.2
No vit A supplement	0.9	1.0	1.0	1.0	0.4	1.0	0.1	1.0	1.3	2.4	6.3	1.0	2.9	3.0	1.4	NA	1.7	2.3	1.1	0.7	0.5	1.0	0.4	1.8	1.5	2.1	1.1	NA	0.9	0.6	3.6	0.5	31.1	1.4	0.3
No iodized salt	1.6	3.9	1.0	0.9	0.3	0.4	2.3	0.7	1.1	1.3	3.4	1.0	0.6	1.0	0.8	NA	0.0	44.8	1.0	1.1	1.6	1.0	2.4	1.4	0.5	4.0	3.9	NA	0.7	3.7	1.3	0.8	1.0	1.1	3.2
Unsafe water	1.0	0.8	1.0	0.6	2.3	2.1	0.3	1.0	0.8	0.9	2.5	0.8	0.7	24.2	1.4	NA	0.4	1.0	0.5	0.4	0.9	1.0	1.6	1.3	3.5	1.0	0.3	NA	0.8	1.0	2.6	1.3	0.0	0.9	0.5
No care seeking for susp pneu	1.0	0.7	0.3	1.0	1.7	0.9	1.0	0.9	10.9	1.0	0.3	0.8	1.0	1.0	0.7	NA	10.3	1.0	0.6	0.6	2.1	1.0	0.4	0.5	1.0	1.0	0.7	NA	1.0	1.0	1.0	0.5	1.0	1.0	2.9

Label: odds ratios

>=4.0 [2.0, 4.0) [1.3, 2.0) [1.1, 1.3) [1.0, 1.1) <1.0

© 2020 Li Z et al. *JAMA Network Open.*

eFigure 16. Relative Ranking of 17 Factors Associated With Child Anthropometric Failures From Fully Adjusted Models for the Pooled Sample, Excluding Source of Drinking Water, Sanitation Facility, and Household Air Quality
A) stunting, B) underweight, and C) wasting, odds ratio (OR) and 95% confidence interval (CI)

A) Stunting

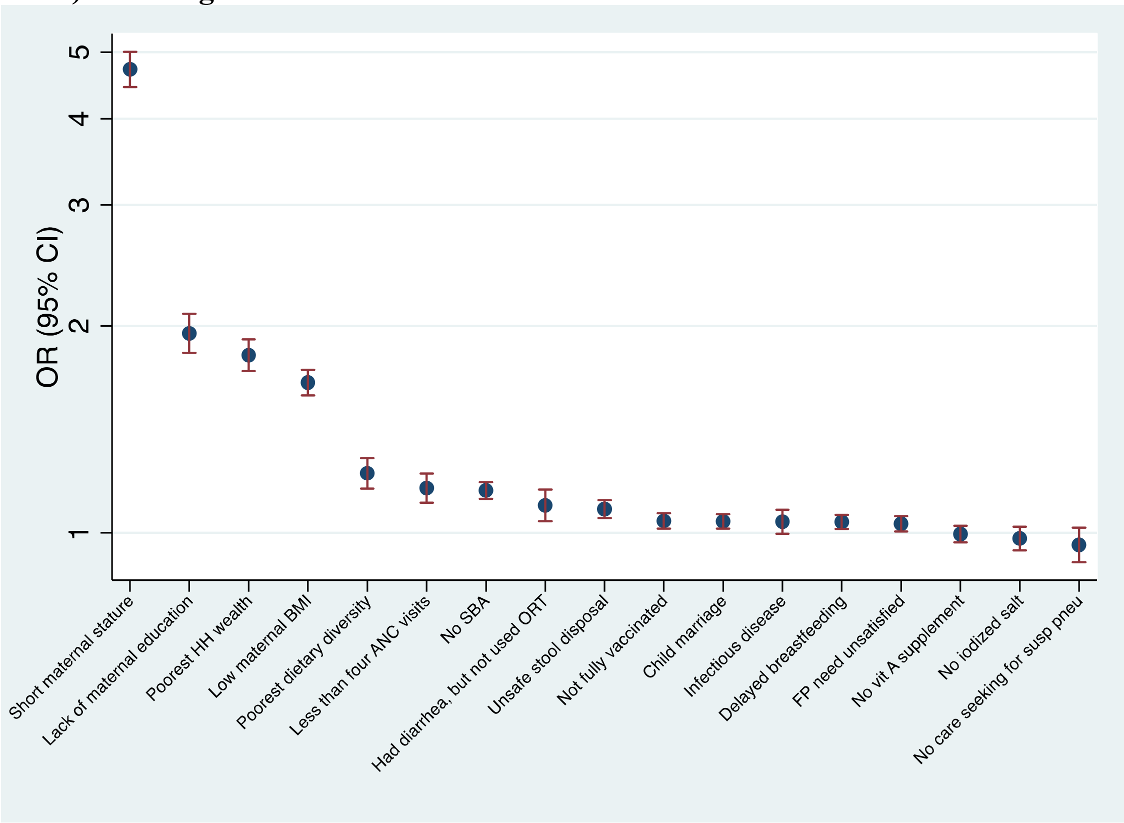

Note:

1. We use logarithmic scale for the y axis.
2. Short maternal statue: maternal height <145cm; low maternal BMI: maternal BMI <18.5 kg/m2; child marriage: mother's age at marriage <18 years old; delayed breastfeeding: child was not initially breastfed within one hour after born; infectious disease: child was caught by infectious diseases two weeks prior to the survey.
3. Abbreviations - HH: household, BMI: body mass index; SBA: skilled birth attendant; ANC: antenatal care; ORT: oral rehydration therapy; FP: family planning; vit: vitamin; susp pneu: suspected pneumonia.

© 2020 Li Z et al. *JAMA Network Open.*

B) Underweight

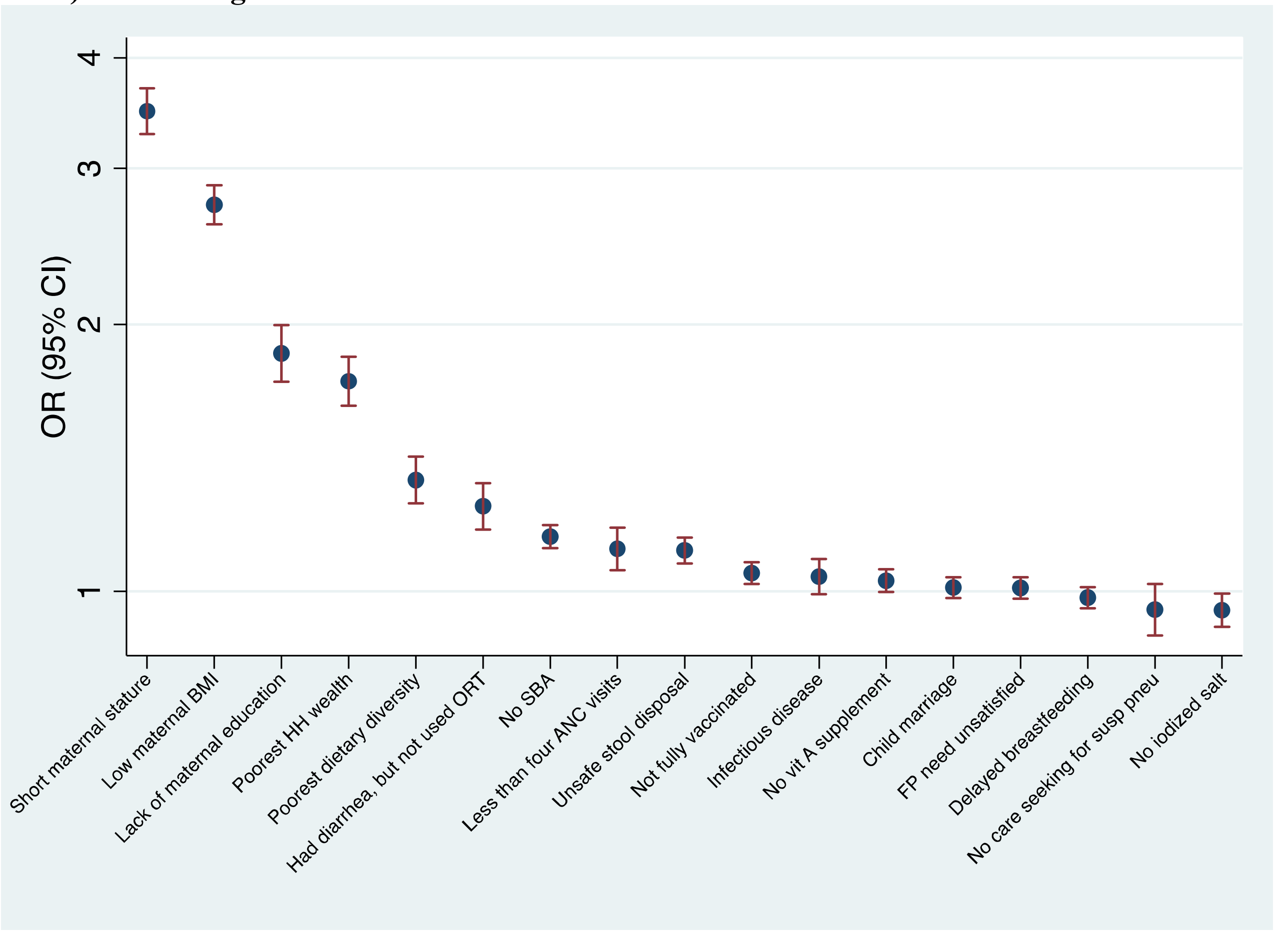

© 2020 Li Z et al. *JAMA Network Open.*

C) Wasting

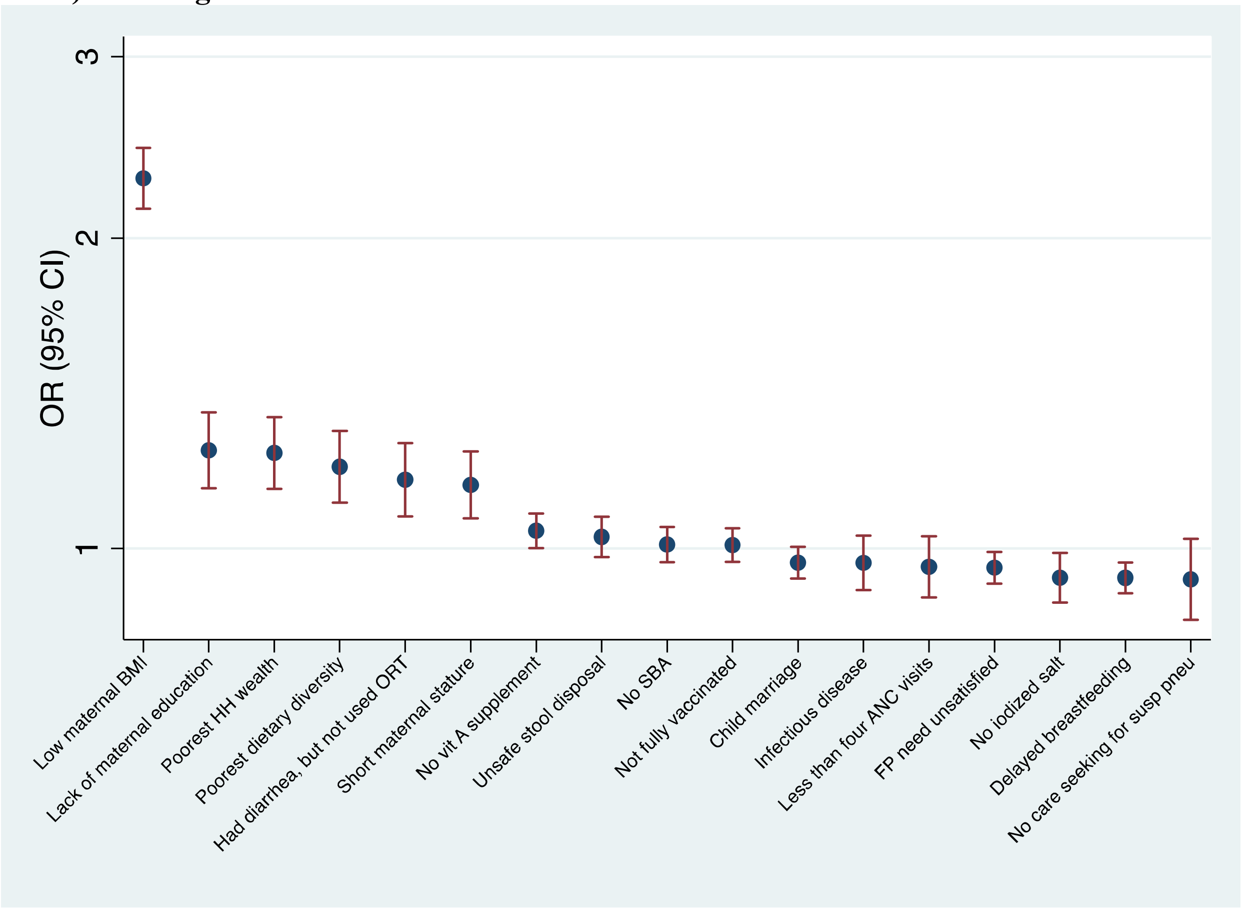

© 2020 Li Z et al. *JAMA Network Open.*

eFigure 17. Country-Specific Ranking of 17 Factors Associated With Child Anthropometric Failures From Fully Adjusted Models for the Pooled Sample, Excluding Source of Drinking Water, Sanitation Facility, and Household Air Quality
A) stunting, B) underweight, and C) wasting

A) Stunting, children 12-59 months old, total n=299,353

	BJ	BF	BI	CM	CI	CD	ET	GM	GH	GN	HT	IN	KE	KG	KM	LS	LR	MW	ML	MZ	MM	NA	NP	NE	PE	ST	SL	SZ	TD	TJ	TZ	TG	UG	ZM	ZW
Short maternal stature	1	2	1	3	2	1	1	2	1	17	1	1	1	1	4	5	1	1	8	1	1	2	1	3	1	2	2	1	4	1	1	1	1	1	1
Lack of maternal education	4	1	2	2	1	3	4	17	2	1	4	2	5	13	1	2	4	2	2	2	9	5	4	1	3	12	1	2	1	6	4	2	2	4	2
Poorest HH wealth	3	3	3	1	4	2	2	1	4	2	2	4	2	8	6	1	2	4	1	3	4	1	2	14	2	1	3	4	13	7	2	3	4	2	3
Low maternal BMI	5	4	4	5	3	4	5	8	6	15	3	3	3	2	2	4	3	3	3	4	2	4	3	4	4	6	4	3	2	4	3	4	5	3	4
Poor dietary diversity	16	5	9	4	15	6	3	12	13	4	13	5	10	7	17	12	5	13	4	6	17	15	16	2	7	5	11	17	6	2	5	6	6	11	11
No SBA	6	6	7	14	16	14	10	7	3	7	7	9	4	3	12	7	14	8	11	8	3	10	9	12	5	11	13	6	5	8	8	11	8	6	7
Less than four ANC visits	2	17	17	7	6	17	15	3	8	3	5	6	6	15	9	6	11	17	5	5	5	9	8	5	17	15	10	14	3	9	17	5	3	14	5
Had diarrhea, but not used ORT	7	11	13	10	17	11	9	13	17	5	11	8	9	16	3	3	8	6	6	9	16	3	17	16	13	14	17	5	7	3	6	16	12	8	17
Unsafe stool disposal	13	7	6	8	7	10	17	6	7	9	8	7	15	10	16	13	13	10	7	15	8	7	5	8	8	7	15	15	8	5	9	8	7	15	10
Not fully vaccinated	12	8	5	13	9	16	13	5	5	12	12	13	12	9	5	14	12	11	16	13	12	6	13	11	12	4	8	7	12	16	11	7	16	13	8
Infectious disease	10	9	11	12	12	12	6	14	9	8	16	11	14	4	11	17	10	16	17	7	13	8	6	6	15	3	14	16	10	15	14	14	15	7	6
Child marriage	9	14	10	17	11	8	14	9	10	14	15	10	8	11	10	11	15	14	14	10	6	16	11	9	10	8	12	12	15	14	16	10	14	5	9
Delayed breastfeeding	11	13	12	16	14	9	8	10	14	16	10	12	7	6	14	10	6	5	15	14	14	11	12	10	14	10	7	13	17	12	13	12	11	10	13
FP need unsatisfied	8	12	15	11	13	13	7	4	15	13	9	14	16	5	8	15	9	7	13	11	10	12	10	13	9	16	6	11	9	11	12	13	9	9	12
No vit A supplement	17	16	8	9	8	5	16	11	12	6	17	15	17	12	15	16	7	12	10	16	7	13	7	7	6	9	9	9	16	17	15	9	13	16	14
No iodized salt	15	15	16	6	5	15	12	16	11	10	14	16	13	14	7	9	17	15	12	12	11	17	15	15	16	17	5	8	11	13	7	17	10	17	16
No care seeking for susp pneu	14	10	14	15	10	7	11	15	16	11	6	17	11	17	13	8	16	9	9	17	15	14	14	17	11	13	16	10	14	10	10	15	17	12	15

Label:
Rank 1st
Rank 2nd
Rank 3rd-4th
Rank 5th-7th
Rank 8th-12nd
Rank 13th-17th

Note:

1. BJ= Benin, BF= Burkina Faso, BI= Burundi, CM=Cameroon, CI=Côte d'Ivoire, CD=The Democratic Republic of the Congo, ET=Ethiopia, GM=Gambia, GH=Ghana, GN=Guinea, HT=Haiti, IN=India, KE=Kenya, KG=Kyrgyzstan, KM=Comoros, LS=Lesotho, LR=Liberia, MW=Malawi, ML=Mali, MZ=Mozambique, MM=Myanmar, NA=Namibia, NP=Nepal, NE=Niger, PE=Peru, ST= São Tomé and Príncipe, SL=Sierra Leone, SZ=Swaziland, TD=Chad, TJ=Tajikistan, TZ=Tanzania, TG=Togo, UG=Uganda, ZM=Zambia, ZW=Zimbabwe.
2. Short maternal statue: maternal height <145cm; low maternal BMI: maternal BMI <18.5 kg/m2; child marriage: mother's age at marriage <18 years old; delayed breastfeeding: child was not initially breastfed within one hour after born; infectious disease: child was caught by infectious diseases two weeks prior to the survey.
3. Abbreviations - HH: household, BMI: body mass index; SBA: skilled birth attendant; ANC: antenatal care; ORT: oral rehydration therapy; FP: family planning; vit: vitamin; susp pneu: suspected pneumonia.

© 2020 Li Z et al. *JAMA Network Open.*

B) Underweight, children 12-59 months old, total n=299,353

	BJ	BF	BI	CM	CI	CD	ET	GM	GH	GN	HT	IN	KE	KG	KM	LS	LR	MW	ML	MZ	MM	NA	NP	NE	PE	ST	SL	SZ	TD	TJ	TZ	TG	UG	ZM	ZW
Short maternal stature	1	1	2	5	3	1	1	1	3	8	2	1	1	9	3	2	1	1	9	4	1	17	2	2	3	3	2	1	10	4	1	2	1	1	5
Lack of maternal education	3	3	17	2	1	5	3	13	1	5	1	3	4	10	2	1	11	4	1	1	15	9	5	5	4	17	17	5	2	14	5	4	2	5	2
Poorest HH wealth	5	4	3	4	6	2	7	6	8	9	4	4	3	14	7	3	5	3	3	5	10	1	3	8	1	2	3	4	9	13	3	5	4	3	10
Low maternal BMI	2	2	1	1	2	3	2	3	2	1	3	2	2	1	1	5	2	2	2	2	2	5	1	1	2	1	1	2	1	1	2	3	3	2	1
Poor dietary diversity	10	11	14	3	8	6	5	10	9	4	16	5	16	3	11	17	3	5	4	6	8	12	6	6	17	14	8	16	7	3	10	11	7	17	17
No SBA	4	5	5	8	15	8	13	5	4	2	13	9	5	8	10	7	9	9	8	17	4	6	11	14	9	8	12	8	6	6	15	8	8	7	9
Less than four ANC visits	9	17	15	6	17	17	4	2	12	3	7	8	6	16	14	9	13	17	13	3	3	13	9	15	16	12	4	17	5	5	17	1	5	4	8
Had diarrhea, but not used ORT	8	10	6	7	12	12	6	4	16	6	9	6	7	7	6	4	4	6	6	7	17	3	17	10	7	4	6	3	4	2	11	7	12	13	15
Unsafe stool disposal	16	6	10	11	7	9	9	9	6	13	11	7	8	4	8	10	8	7	7	10	6	4	7	3	11	11	14	11	8	7	7	12	6	15	12
Not fully vaccinated	11	9	7	9	11	14	11	14	10	12	8	12	9	17	4	14	12	8	10	11	11	8	12	12	13	6	7	7	3	15	8	6	13	8	13
Infectious disease	6	7	16	14	5	10	16	17	5	14	17	11	12	2	12	16	10	11	11	8	5	16	4	4	8	16	16	15	13	17	9	13	17	6	7
Child marriage	15	13	12	17	14	13	15	12	17	16	15	13	15	13	15	8	15	12	14	14	7	15	13	9	14	7	15	9	15	12	16	9	15	10	6
Delayed breastfeeding	14	15	11	15	16	15	14	15	14	17	10	14	14	11	9	6	7	10	12	15	12	7	15	11	10	9	11	10	17	9	13	15	10	9	11
FP need unsatisfied	7	8	13	12	10	16	10	11	13	11	12	15	10	6	5	12	6	14	16	12	13	11	16	13	15	15	9	6	12	11	4	10	14	11	4
No vit A supplement	12	14	9	13	9	11	17	8	11	7	14	10	13	5	13	15	14	15	15	13	9	10	10	7	6	5	13	14	16	16	14	14	9	16	16
No iodized salt	17	12	4	10	4	4	8	16	7	10	6	16	17	15	17	11	17	13	17	16	14	14	8	16	5	10	10	13	14	8	6	16	11	14	3
No care seeking for susp pneu	13	16	8	16	13	7	12	7	15	15	5	17	11	12	16	13	16	16	5	9	16	2	14	17	12	13	5	12	11	10	12	17	16	12	14

Label:
Rank 1st; Rank 2nd; Rank 3rd-4th; Rank 5th-7th; Rank 8th-12nd; Rank 13th-17th

© 2020 Li Z et al. *JAMA Network Open.*

C) Wasting, children 12-59 months old, total n=299,353

	BJ	BF	BI	CM	CI	CD	ET	GM	GH	GN	HT	IN	KE	KG	KM	LS	LR	MW	ML	MZ	MM	NA	NP	NE	PE	ST	SL	SZ	TD	TJ	TZ	TG	UG	ZM	ZW
Short maternal stature	2	5	16	9	8	3	6	8	9	11	15	5	11	8	2	13	1	1	17	1	14	11	17	1	3	17	17	11	1	10	2	3	3	5	2
Lack of maternal education	12	4	17	6	1	1	4	6	1	5	1	4	1	6	3	8	11	17	2	4	16	1	4	13	12	7	3	16	4	16	7	5	17	17	9
Poorest HH wealth	17	13	3	1	5	4	3	17	15	16	4	2	2	7	4	6	8	15	5	2	6	3	15	3	2	14	13	2	14	17	15	2	2	2	4
Low maternal BMI	1	2	1	4	2	6	1	2	3	3	2	1	5	2	9	7	3	3	1	3	1	7	1	2	1	10	1	1	2	1	1	1	1	6	1
Poor dietary diversity	11	1	15	2	9	8	5	4	17	2	16	3	16	13	1	2	4	5	15	6	2	17	3	10	16	16	2	7	9	8	4	16	11	11	3
No SBA	8	10	5	3	15	2	8	9	6	4	11	13	7	10	16	5	9	7	10	16	7	10	11	16	7	13	10	15	11	12	9	7	6	3	13
Less than four ANC visits	16	16	12	17	17	17	2	1	2	1	5	15	3	16	15	15	12	11	7	17	4	6	10	17	14	1	9	17	3	3	16	17	16	1	6
Had diarrhea, but not used ORT	4	6	4	7	13	9	7	11	5	6	12	8	4	9	5	9	5	9	9	13	15	8	7	5	6	8	4	10	6	2	17	11	15	7	14
Unsafe stool disposal	15	3	13	16	7	16	9	7	12	15	8	7	6	14	6	12	10	14	8	9	3	5	5	4	13	4	5	6	5	6	6	15	8	16	11
Not fully vaccinated	6	15	7	8	14	11	14	15	14	13	9	9	9	4	8	3	13	6	11	7	11	12	13	7	4	9	12	12	7	11	5	8	14	12	15
Infectious disease	9	11	10	11	3	5	16	16	16	10	14	11	15	17	14	17	16	4	3	8	9	16	6	15	5	15	11	5	13	15	3	10	9	14	8
Child marriage	10	12	6	15	4	12	10	12	8	12	17	14	14	5	12	11	15	8	13	14	10	9	14	11	9	6	8	14	10	7	14	4	10	8	7
Delayed breastfeeding	14	14	9	5	16	10	13	10	13	17	7	16	12	15	13	10	7	12	6	11	5	4	9	12	10	5	14	4	8	4	8	9	7	15	12
FP need unsatisfied	13	9	14	10	10	14	15	13	11	14	10	12	13	11	7	16	14	10	14	15	13	14	12	8	8	11	7	3	16	13	10	13	13	9	17
No vit A supplement	7	7	11	13	12	7	17	5	7	7	6	6	8	3	10	4	6	13	16	5	17	15	16	6	17	3	15	8	15	14	12	6	12	10	16
No iodized salt	5	17	2	14	11	15	11	14	10	8	3	10	17	12	17	14	17	2	12	10	12	13	2	14	15	2	16	9	17	5	11	12	4	4	5
No care seeking for susp pneu	3	8	8	12	6	13	12	3	4	9	13	17	10	1	11	1	2	16	4	12	8	2	8	9	11	12	6	13	12	9	13	14	5	13	10

Label:
Rank 1st — Rank 2nd — Rank 3rd-4th — Rank 5th-7th — Rank 8th-12nd — Rank 13th-17th

© 2020 Li Z et al. *JAMA Network Open.*

eFigure 18. Country-Specific Odds Ratios for 17 Factors Associated With Child Anthropometric Failures From Fully Adjusted Models for the Pooled Sample, Excluding Source of Drinking Water, Sanitation Facility, and Household Air Quality
A) stunting, B) underweight, and C) wasting

A) Stunting, children 12-59 months old, total n=299,353

	BJ	BF	BI	CM	CI	CD	ET	GM	GH	GN	HT	IN	KE	KG	KM	LS	LR	MW	ML	MZ	MM	NA	NP	NE	PE	ST	SL	SZ	TD	TJ	TZ	TG	UG	ZM	ZW
Short maternal stature	8.3	7.4	9.4	2.8	3.0	3.5	2.9	1.6	6.3	0.8	11.1	4.4	4.0	3.2	1.5	2.7	3.9	7.3	1.2	4.5	5.2	5.1	9.7	1.7	9.7	3.1	2.4	9.0	1.3	4.5	6.1	15.1	13.0	5.0	6.0
Lack of maternal education	2.3	7.7	7.1	3.7	31.6	1.8	1.8	0.8	3.0	5.4	1.6	1.9	1.2	0.8	2.3	7.6	2.1	3.8	3.2	3.9	1.1	1.8	1.5	6.9	3.2	1.0	2.9	4.8	2.2	1.3	1.7	3.1	4.6	1.8	5.6
Poorest HH wealth	2.4	2.0	3.0	4.4	1.9	2.8	2.2	1.9	1.8	1.8	4.3	1.6	2.8	1.3	1.3	11.2	2.3	1.9	3.5	2.8	1.4	6.3	3.1	0.9	5.7	4.2	1.6	2.3	1.0	1.2	2.4	2.9	2.0	2.0	2.6
Low maternal BMI	1.7	2.0	2.1	1.8	2.5	1.6	1.7	1.0	1.3	0.9	1.8	1.6	1.4	2.8	2.1	3.5	2.3	2.5	2.1	1.8	1.7	2.1	1.6	1.6	2.2	1.9	1.4	4.7	1.5	1.6	1.8	2.4	1.4	2.0	2.1
Poor dietary diversity	0.9	1.5	1.1	1.9	0.9	1.3	2.1	1.0	1.1	1.7	1.1	1.2	1.1	1.3	0.7	0.8	1.3	1.0	2.0	1.5	0.8	0.9	0.7	1.7	1.2	1.9	1.0	0.5	1.2	1.7	1.4	1.4	1.3	1.0	1.0
No SBA	1.3	1.3	1.2	1.1	0.8	1.0	1.0	1.1	1.8	1.2	1.1	1.1	1.3	1.8	1.0	1.6	0.9	1.1	1.2	1.1	1.5	1.3	1.0	1.0	1.6	1.1	1.0	1.8	1.2	1.2	1.0	1.1	1.1	1.1	1.2
Less than four ANC visits	2.7	0.6	0.0	1.4	1.5	0.7	0.9	1.2	1.2	1.7	1.4	1.2	1.2	0.7	1.2	2.4	1.1	0.3	1.4	1.6	1.2	1.4	1.1	1.4	0.9	0.7	1.1	0.9	1.4	1.2	0.7	1.7	3.8	1.0	1.4
Had diarrhea, but not used ORT	1.2	1.1	1.0	1.1	0.8	1.1	1.1	0.9	0.6	1.3	1.1	1.1	1.1	0.6	1.6	5.9	1.2	1.3	1.3	1.1	0.9	2.6	0.7	0.8	1.0	0.8	0.5	2.1	1.2	1.6	1.3	0.9	0.9	1.0	0.7
Unsafe stool disposal	1.0	1.2	1.3	1.3	1.4	1.2	0.7	1.2	1.3	1.1	1.1	1.1	0.9	1.1	0.9	0.8	1.0	1.1	1.3	0.8	1.1	1.7	1.5	1.3	1.1	1.4	0.9	0.9	1.2	1.3	1.0	1.3	1.2	1.0	1.0
Not fully vaccinated	1.0	1.1	1.3	1.1	1.2	0.9	1.0	1.2	1.4	1.0	1.1	1.0	1.0	1.1	1.4	0.7	1.0	1.1	0.9	0.9	1.0	1.8	0.8	1.0	1.0	2.0	1.1	1.6	1.0	0.7	1.0	1.3	0.8	1.0	1.2
Infectious disease	1.0	1.1	1.1	1.1	1.0	1.0	1.1	0.9	1.1	1.1	0.9	1.1	0.9	1.5	1.1	0.5	1.1	0.8	0.9	1.2	1.0	1.4	1.2	1.3	0.9	2.4	0.9	0.7	1.0	0.8	0.9	1.0	0.8	1.0	1.3
Child marriage	1.1	0.9	1.1	0.9	1.1	1.2	0.9	1.0	1.1	0.9	1.0	1.1	1.1	1.0	1.1	1.0	0.8	1.0	1.0	1.0	1.2	0.6	1.0	1.1	1.0	1.3	1.0	1.3	1.0	0.8	0.8	1.1	0.8	1.1	1.2
Delayed breastfeeding	1.0	1.0	1.0	1.0	0.9	1.2	1.1	1.0	0.8	0.9	1.1	1.1	1.1	1.4	1.0	1.0	1.2	1.4	1.0	0.9	1.0	1.3	0.9	1.0	0.9	1.1	1.2	1.1	0.9	0.9	0.9	1.0	0.9	1.0	1.0
FP need unsatisfied	1.2	1.0	1.0	1.1	1.0	1.0	1.1	1.2	0.7	1.0	1.1	1.0	0.9	1.4	1.2	0.6	1.1	1.2	1.1	1.0	1.1	1.2	1.0	1.0	1.0	0.6	1.2	1.3	1.1	1.0	1.0	1.0	1.0	1.0	1.0
No vit A supplement	0.9	0.8	1.1	1.2	1.4	1.4	0.8	1.0	1.1	1.2	0.9	1.0	0.9	1.0	0.9	0.6	1.2	1.0	1.2	0.8	1.2	1.1	1.1	1.3	1.4	1.2	1.1	1.5	0.9	0.6	0.9	1.2	0.8	0.9	1.0
No iodized salt	0.9	0.8	0.9	1.4	1.5	0.9	1.0	0.8	1.1	1.0	1.0	1.0	1.0	0.8	1.3	1.2	0.6	0.9	1.1	0.9	1.1	0.6	0.7	0.9	0.9	0.6	1.4	1.5	1.0	0.8	1.1	0.9	1.0	0.8	0.8
No care seeking for susp pneu	0.9	1.1	1.0	1.0	1.2	1.3	1.0	0.8	0.6	1.0	1.2	0.9	1.0	0.6	1.0	1.5	0.8	1.1	1.2	0.8	0.9	1.0	0.8	0.6	1.0	1.0	0.8	1.3	1.0	1.0	1.0	1.0	0.7	1.0	0.9

Label: odds ratios

>=4.0   [2.0, 4.0)   [1.3, 2.0)   [1.1, 1.3)   [1.0, 1.1)   <1.0

Note:

1. BJ= Benin, BF= Burkina Faso, BI= Burundi, CM=Cameroon, CI=Côte d'Ivoire, CD=The Democratic Republic of the Congo, ET=Ethiopia, GM=Gambia, GH=Ghana, GN=Guinea, HT=Haiti, IN=India, KE=Kenya, KG=Kyrgyzstan, KM=Comoros, LS=Lesotho, LR=Liberia, MW=Malawi, ML=Mali, MZ=Mozambique, MM=Myanmar, NA=Namibia, NP=Nepal, NE=Niger, PE=Peru, ST= São Tomé and Príncipe, SL=Sierra Leone, SZ=Swaziland, TD=Chad, TJ=Tajikistan, TZ=Tanzania, TG=Togo, UG=Uganda, ZM=Zambia, ZW=Zimbabwe.
2. Short maternal statue: maternal height <145cm; low maternal BMI: maternal BMI <18.5 kg/m2; child marriage: mother’s age at marriage <18 years old; delayed breastfeeding: child was not initially breastfed within one hour after born; infectious disease: child was caught by infectious diseases two weeks prior to the survey.
3. Abbreviations - HH: household, BMI: body mass index; SBA: skilled birth attendant; ANC: antenatal care; ORT: oral rehydration therapy; FP: family planning; vit: vitamin; susp pneu: suspected pneumonia.

© 2020 Li Z et al. *JAMA Network Open.*

B) Underweight, children 12-59 months old, total n=299,353

	BJ	BF	BI	CM	CI	CD	ET	GM	GH	GN	HT	IN	KE	KG	KM	LS	LR	MW	ML	MZ	MM	NA	NP	NE	PE	ST	SL	SZ	TD	TJ	TZ	TG	UG	ZM	ZW
Short maternal stature	4.2	6.1	3.7	2.6	2.4	3.3	4.0	6.3	2.4	1.5	4.9	3.4	4.9	1.0	1.8	5.4	6.4	6.5	1.2	4.1	4.2	0.2	2.7	1.6	6.0	3.0	1.9	4.6	1.1	1.7	5.9	5.4	6.8	4.3	1.4
Lack of maternal education	2.4	1.7	0.7	5.6	20.7	1.7	2.6	0.9	13.2	1.7	8.4	1.8	2.2	1.0	2.9	33.5	1.0	1.8	3.7	5.7	0.7	1.2	1.4	1.5	2.8	0.5	0.8	1.9	1.5	0.9	1.3	2.4	5.5	1.6	2.2
Poorest HH wealth	1.3	1.4	3.1	2.8	1.5	2.9	1.7	1.2	1.2	1.4	2.3	1.7	3.2	0.9	1.3	3.4	1.4	2.1	1.7	2.4	1.0	5.1	1.6	1.2	17.3	4.0	1.6	2.5	1.2	0.9	2.0	1.9	2.2	2.3	1.2
Low maternal BMI	3.5	3.7	6.8	6.0	7.5	2.7	2.8	2.0	4.0	2.7	4.3	2.6	3.3	5.7	3.1	2.6	5.6	3.5	2.7	4.8	3.4	2.0	4.1	3.2	7.1	5.6	2.1	4.3	2.8	3.2	3.2	5.1	3.3	2.7	6.2
Poor dietary diversity	1.1	1.0	1.0	3.1	1.2	1.4	2.0	1.0	1.2	1.7	0.7	1.4	0.8	1.9	1.0	0.4	1.7	1.6	1.6	1.7	1.2	0.9	1.3	1.4	0.6	0.8	1.3	0.5	1.2	1.9	1.1	1.2	1.4	0.9	0.5
No SBA	1.5	1.4	1.5	1.4	0.7	1.2	1.0	1.3	1.6	1.8	1.1	1.1	1.3	1.0	1.2	1.2	1.1	1.3	1.3	0.8	1.5	1.5	1.0	1.0	1.1	1.4	1.1	1.2	1.3	1.2	0.9	1.2	1.2	1.2	1.2
Less than four ANC visits	1.2	0.1	0.8	1.9	0.5	0.4	2.1	2.2	0.9	1.7	1.5	1.1	1.3	0.8	0.9	1.0	0.9	0.6	1.0	4.2	1.6	0.8	1.2	1.0	0.8	1.1	1.5	0.2	1.4	1.5	0.8	7.8	1.6	1.9	1.2
Had diarrhea, but not used ORT	1.2	1.1	1.5	1.5	1.0	1.1	1.9	1.5	0.6	1.6	1.3	1.2	1.2	1.0	1.4	2.7	1.6	1.3	1.3	1.6	0.4	3.6	0.8	1.1	1.2	3.0	1.4	2.9	1.4	1.9	1.1	1.3	1.0	0.9	0.7
Unsafe stool disposal	0.9	1.2	1.2	1.2	1.2	1.2	1.1	1.1	1.4	1.1	1.2	1.1	1.1	1.5	1.2	0.9	1.2	1.3	1.3	1.1	1.3	3.2	1.2	1.6	1.1	1.2	1.0	0.9	1.2	1.1	1.2	1.2	1.4	0.9	1.0
Not fully vaccinated	1.1	1.1	1.4	1.3	1.1	1.0	1.0	0.9	1.0	1.1	1.3	1.0	1.0	0.8	1.6	0.6	0.9	1.3	1.1	1.0	1.0	1.4	0.9	1.0	1.0	1.7	1.3	1.5	1.4	0.8	1.2	1.4	0.9	1.2	0.9
Infectious disease	1.3	1.2	0.8	1.1	1.6	1.2	0.9	0.6	1.5	1.0	0.7	1.0	1.0	2.0	1.0	0.5	1.1	1.1	1.1	1.3	1.5	0.2	1.4	1.5	1.1	0.7	0.9	0.7	1.0	0.7	1.2	1.0	0.7	1.3	1.2
Child marriage	1.0	1.0	1.1	1.0	0.8	1.1	0.9	1.0	0.6	1.0	0.9	1.0	0.9	0.9	0.9	1.0	0.9	1.1	1.0	0.9	1.2	0.5	0.9	1.1	1.0	1.4	1.0	0.9	0.9	0.9	0.9	1.2	0.8	1.1	1.3
Delayed breastfeeding	1.0	0.9	1.2	1.0	0.6	1.0	1.0	0.8	0.7	0.9	1.2	1.0	0.9	1.0	1.2	1.2	1.3	1.2	1.0	0.9	0.9	1.5	0.9	1.0	1.1	1.2	1.1	0.9	0.8	1.0	1.0	0.9	1.1	1.1	1.0
FP need unsatisfied	1.2	1.2	1.0	1.2	1.1	0.8	1.0	1.0	0.8	1.1	1.1	1.0	1.0	1.1	1.5	0.7	1.4	1.0	1.0	1.0	0.8	1.0	0.9	1.0	0.9	0.7	1.2	1.8	1.0	1.0	1.3	1.2	0.9	1.0	1.4
No vit A supplement	1.1	0.9	1.2	1.2	1.2	1.1	0.8	1.2	1.0	1.6	1.1	1.0	0.9	1.3	1.0	0.6	0.9	0.8	1.0	1.0	1.0	1.2	1.1	1.3	1.5	2.7	1.0	0.7	0.8	0.8	0.9	1.0	1.1	0.9	0.7
No iodized salt	0.9	1.0	1.9	1.2	1.6	1.8	1.2	0.7	1.3	1.3	1.5	0.9	0.7	0.9	0.6	0.8	0.3	1.1	1.0	0.9	0.8	0.6	1.2	0.9	1.5	1.2	1.2	0.9	0.9	1.1	1.3	0.9	1.0	0.9	1.6
No care seeking for susp pneu	1.0	0.9	1.3	1.0	0.9	1.2	1.0	1.2	0.7	1.0	1.6	0.9	1.0	0.9	0.7	0.7	0.8	0.8	1.4	1.2	0.7	4.8	0.9	0.6	1.0	1.0	1.4	0.9	1.0	1.0	1.0	0.7	0.8	1.0	0.9

Label: odds ratios

>=4.0 [2.0, 4.0) [1.3, 2.0) [1.1, 1.3) [1.0, 1.1) <1.0

© 2020 Li Z et al. *JAMA Network Open.*

C) Wasting, children 12-59 months old, total n=299,353

	BJ	BF	BI	CM	CI	CD	ET	GM	GH	GN	HT	IN	KE	KG	KM	LS	LR	MW	ML	MZ	MM	NA	NP	NE	PE	ST	SL	SZ	TD	TJ	TZ	TG	UG	ZM	ZW
Short maternal stature	2.0	1.4	0.5	1.3	1.0	1.8	1.7	1.0	1.0	0.9	0.7	1.2	1.0	1.0	1.5	0.3	5.1	8.9	0.0	2.7	0.6	1.0	0.5	3.2	2.2	0.4	0.2	1.0	5.4	1.0	2.0	2.2	2.6	1.4	3.5
Lack of maternal education	1.0	1.7	0.1	1.5	3.6	4.1	1.8	1.3	34.1	1.6	7.0	1.2	3.7	1.1	1.5	1.0	1.0	0.2	2.5	1.9	0.6	5.2	2.0	0.9	1.0	1.3	1.8	0.2	1.4	0.6	1.2	1.5	0.4	0.6	1.0
Poorest HH wealth	0.5	0.9	2.2	4.4	1.1	1.6	1.9	0.6	0.3	0.7	1.5	1.3	2.9	1.0	1.5	1.9	1.0	0.7	1.2	2.1	1.2	4.7	0.7	1.7	2.6	0.8	0.9	12.3	0.9	0.5	0.6	3.7	4.0	1.8	2.4
Low maternal BMI	5.7	2.7	16.6	2.0	3.1	1.3	3.7	3.2	2.5	2.0	2.8	2.3	1.5	2.8	1.3	1.2	2.6	3.0	2.7	1.9	2.1	1.6	2.7	2.9	11.8	1.1	2.2	48.9	4.7	3.4	2.5	4.4	5.8	1.2	3.7
Poor dietary diversity	1.0	4.5	0.6	2.4	0.9	1.1	1.7	1.7	0.2	4.6	0.6	1.2	0.7	0.8	2.2	3.5	2.0	1.9	0.8	1.7	1.4	0.2	2.2	1.0	0.6	0.7	2.0	1.5	1.1	1.0	1.7	0.6	0.8	1.1	2.6
No SBA	1.2	1.0	1.5	2.3	0.6	1.8	1.3	1.0	1.5	1.7	1.0	1.0	1.2	1.0	0.7	2.0	1.0	1.3	1.0	0.5	1.2	1.2	1.0	0.8	1.3	1.0	1.2	0.2	1.0	0.9	1.2	1.2	1.0	1.6	0.6
Less than four ANC visits	0.6	0.6	1.0	0.5	0.3	0.7	2.7	14.0	2.7	6.9	1.4	0.9	2.1	0.3	0.7	0.3	1.0	0.9	1.1	0.4	1.3	2.5	1.0	0.7	0.9	4.8	1.2	0.1	1.5	2.2	0.5	0.3	0.5	2.5	1.1
Had diarrhea, but not used ORT	1.6	1.4	1.7	1.5	0.7	1.0	1.5	1.0	2.1	1.5	0.8	1.0	1.8	1.0	1.4	1.0	1.9	1.1	1.1	0.9	0.6	1.5	1.4	1.4	1.4	1.3	1.8	1.0	1.3	2.7	0.5	0.9	0.5	1.2	0.6
Unsafe stool disposal	0.7	1.8	1.0	0.8	1.0	0.8	1.2	1.3	0.7	0.7	1.4	1.0	1.4	0.7	1.4	0.4	1.0	0.8	1.1	1.2	1.3	3.0	1.7	1.4	0.9	1.8	1.4	1.6	1.4	1.3	1.3	0.7	0.9	0.8	0.7
Not fully vaccinated	1.3	0.8	1.3	1.4	0.7	0.9	0.9	0.8	0.5	0.8	1.3	1.0	1.1	1.6	1.3	2.5	0.9	1.3	1.0	1.4	0.9	0.8	0.9	1.1	1.7	1.1	1.0	0.8	1.3	1.0	1.6	1.2	0.6	1.0	0.6
Infectious disease	1.2	1.0	1.1	1.0	2.0	1.4	0.8	0.7	0.3	0.9	0.7	1.0	0.8	0.0	0.7	0.1	0.6	2.0	1.3	1.4	1.0	0.3	1.6	0.8	1.5	0.7	1.1	1.7	0.9	0.7	1.9	1.0	0.9	1.0	1.1
Child marriage	1.1	1.0	1.4	0.8	1.3	0.9	1.1	0.9	1.0	0.8	0.6	1.0	0.8	1.4	0.8	0.6	0.6	1.3	0.9	0.7	1.0	1.3	0.9	1.0	1.1	1.3	1.2	0.3	1.1	1.2	0.8	1.5	0.9	1.2	1.1
Delayed breastfeeding	1.0	0.9	1.1	1.5	0.6	0.9	0.9	1.0	0.7	0.6	1.4	0.9	0.9	0.5	0.8	0.9	1.2	0.8	1.2	1.0	1.3	3.7	1.1	0.9	1.1	1.4	0.9	2.1	1.2	1.5	1.2	1.1	0.9	0.9	0.7
FP need unsatisfied	1.0	1.2	1.0	1.3	0.8	0.8	0.8	0.9	0.8	0.8	1.2	1.0	0.9	1.0	1.4	0.2	0.9	1.1	0.8	0.7	0.7	0.4	0.9	1.0	1.2	1.0	1.3	2.5	0.8	0.9	1.1	0.9	0.7	1.1	0.3
No vit A supplement	1.3	1.3	1.0	0.9	0.7	1.2	0.7	1.3	1.5	1.2	1.4	1.0	1.1	2.4	1.1	2.2	1.3	0.8	0.7	1.7	0.5	0.4	0.6	1.3	0.5	3.2	0.8	1.3	0.9	0.9	1.0	1.3	0.7	1.1	0.5
No iodized salt	1.3	0.6	2.2	0.9	0.8	0.8	1.1	0.8	1.0	1.2	1.6	1.0	0.5	1.0	0.5	0.3	0.1	3.7	0.9	1.0	0.8	0.5	2.5	0.8	0.6	4.1	0.7	1.0	0.7	1.5	1.1	0.9	1.4	1.5	2.3
No care seeking for susp pneu	1.7	1.2	1.2	1.0	1.1	0.9	1.0	1.9	2.5	1.0	0.8	0.9	1.0	10.9	1.0	3.9	2.7	0.7	1.3	0.9	1.1	5.1	1.3	1.0	1.0	1.0	1.4	0.3	1.0	1.0	1.0	0.8	1.1	1.0	1.0

Label: odds ratios

>=4.0 [2.0, 4.0) [1.3, 2.0) [1.1, 1.3) [1.0, 1.1) <1.0

© 2020 Li Z et al. *JAMA Network Open.*

eFigure 19. Relative Ranking of 23 Factors Associated With Child Anthropometric Failures From Fully Adjusted Models for the Pooled Sample, Adding Women's Empowerment Factors (n=128,902)
A) stunting, B) underweight, and C) wasting, odds ratio (OR) and 95% confidence interval (CI)

A) Stunting

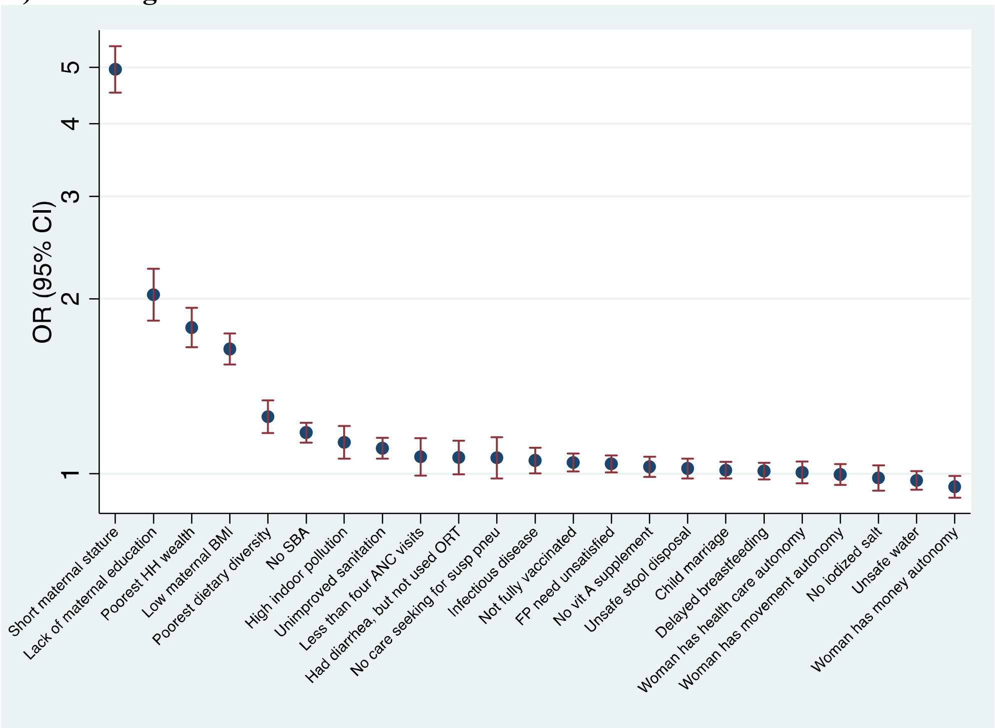

Note:

1. We use logarithmic scale for the y axis.
2. Short maternal statue: maternal height <145cm; low maternal BMI: maternal BMI <18.5 kg/m^2; child marriage: mother's age at marriage <18 years old; delayed breastfeeding: child was not initially breastfed within one hour after born; infectious disease: child was caught by infectious diseases two weeks prior to the survey.
3. Abbreviations - HH: household, BMI: body mass index; SBA: skilled birth attendant; ANC: antenatal care; ORT: oral rehydration therapy; FP: family planning; vit: vitamin; susp pneu: suspected pneumonia.

© 2020 Li Z et al. *JAMA Network Open.*

B) Underweight

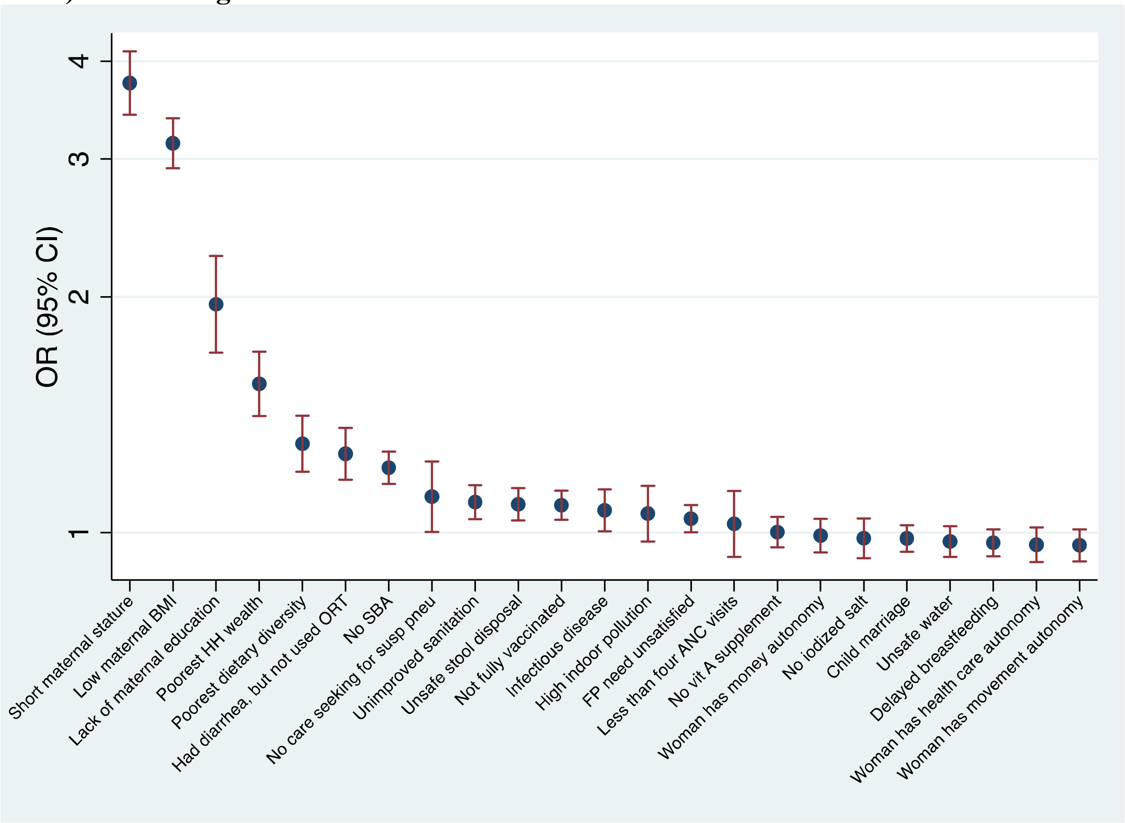

© 2020 Li Z et al. *JAMA Network Open.*

C) Wasting

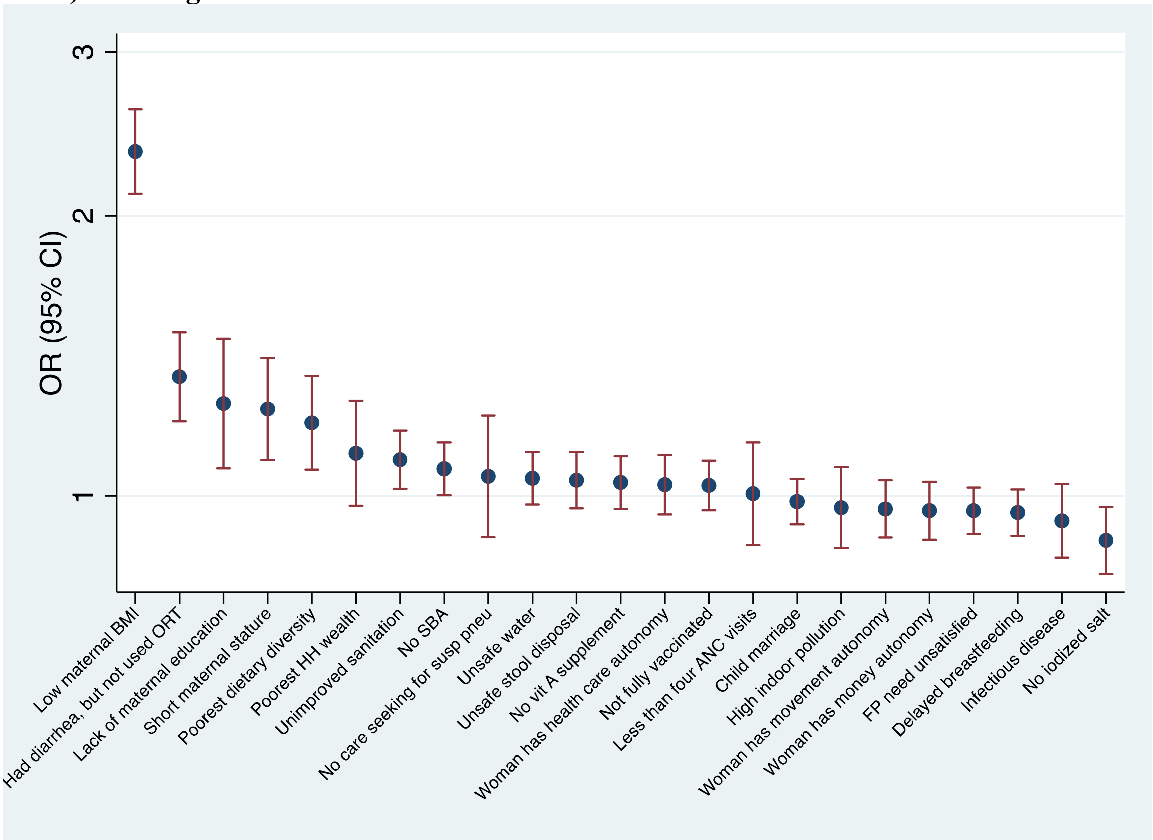

© 2020 Li Z et al. *JAMA Network Open.*
